# Comprehensive insights into the impact of bacterial indole-3-acetic acid on sensory preferences in *Drosophila melanogaster*

**DOI:** 10.1038/s41598-024-58829-7

**Published:** 2024-04-09

**Authors:** Raifa Abdul Aziz, Poornima Ramesh, Kokkarambath Vannadil Suchithra, Paul Stothard, Vanya Kadla Narayana, Shamprasad Varija Raghu, Fo-Ting Shen, Chiu-Chung Young, T. S. Keshava Prasad, Asif Hameed

**Affiliations:** 1 Neurogenetics Lab, Department of Applied Zoology, Mangalore University, Mangalagangothri, Konaje, Mangalore, 574199 India; 2 Center for Systems Biology and Molecular Medicine, Yenepoya Research Centre, Yenepoya (Deemed to be University), Deralakatte, Mangalore, 575018 India; 3 Division of Microbiology and Biotechnology, Yenepoya Research Centre, Yenepoya (Deemed to Be University), Deralakatte, Mangalore, 575018 India; 4 Department of Agricultural, Food and Nutritional Science, University of Alberta, Edmonton, AB T6G 2P5 Canada; 5 Division of Neuroscience, Yenepoya Research Centre, Yenepoya (Deemed to Be University), Deralakatte, Mangalore, 575018 India; 6 Department of Soil & Environmental Sciences, College of Agriculture and Natural Resources, National Chung Hsing University, Taichung, 402 Taiwan; 7 Innovation and Development Center of Sustainable Agriculture, National Chung Hsing University, Taichung, 402 Taiwan

**Keywords:** Host–pathogen interaction, Quantitative proteomics, *Pseudomonas juntendi*, Gut-brain axis, Microbial ecology, Bacterial genomics, Proteomics

## Abstract

Several bacteria of environmental and clinical origins, including some human-associated strains secrete a cross-kingdom signaling molecule indole-3-acetic acid (IAA). IAA is a tryptophan (trp) derivative mainly known for regulating plant growth and development as a hormone. However, the nutritional sources that boost IAA secretion in bacteria and the impact of secreted IAA on non-plant eukaryotic hosts remained less explored. Here, we demonstrate significant trp-dependent IAA production in *Pseudomonas juntendi* NEEL19 when provided with ethanol as a carbon source in liquid cultures. IAA was further characterized to modulate the odor discrimination, motility and survivability in *Drosophila melanogaster.* A detailed analysis of IAA-fed fly brain proteome using high-resolution mass spectrometry showed significant (fold change, ± 2; *p* ≤ 0.05) alteration in the proteins governing neuromuscular features, audio-visual perception and energy metabolism as compared to IAA-unfed controls. Sex-wise variations in differentially regulated proteins were witnessed despite having similar visible changes in chemo perception and psychomotor responses in IAA-fed flies. This study not only revealed ethanol-specific enhancement in trp-dependent IAA production in *P. juntendi,* but also showed marked behavioral alterations in flies for which variations in an array of proteins governing odor discrimination, psychomotor responses, and energy metabolism are held responsible. Our study provided novel insights into disruptive attributes of bacterial IAA that can potentially influence the eukaryotic gut-brain axis having broad environmental and clinical implications.

## Introduction

Tryptophan (trp) is an essential aromatic amino acid for protein synthesis in humans and is considered to be a key player in the microbiota-gut-brain axis^1,2^. Gut microbiota influences the metabolism of trp and the trp catabolites play a major role in microbiota-host crosstalk in health and disease^3^. Formation of indole-3-acetic acid (IAA) is one of the outcomes of three major trp metabolic pathways (the other two pathways respectively lead to the serotonin and kynurenine biosynthesis) in eukaryotic gut occurring under the direct control of microbiota^4^. However, the ecophysiological significance of indoles in general and IAA in particular when associated with non-plant eukaryotic host systems remain poorly explored.

In plants, gene expression analysis validated a concerted regulatory network including IAA secretion in *Pseudomonas savastanoi* among virulence, fitness and drug efflux^5^. Trp-metabolizing gut microbes regulate adult neurogenesis in the mouse hippocampus^6^. Analysis of healthy human fecal samples revealed the high extracellular secretion of indole (0.3‒6 mM) indicating a possible prevalence of indole-producing bacteria in the gut^7–9^. In chronic kidney disease (CKD) patients, significantly higher mortality and cardiovascular events were recorded in higher IAA (> 3.73 µM) versus lower (< 3.73 µM) IAA groups^10^. Depression and/or anxiety and a decline in cognitive functioning have been reported in a large portion of CKD patients^11^. IAA was found to be associated with a higher risk of impaired cognitive function in patients undergoing hemodialysis^12^. Deciphering the factors promoting bacterial IAA formation and the role played by IAA in modulating the behaviour of eukaryotes will pave the way for a better understanding of the microbial pathogenesis of human diseases facilitating targeted therapeutics.

Bacterial strains such as *Thermoanaerobacter ethanolicus* JW200^13^ and *Geobacillus thermodenitrificans* NG80-2 produce distinct alcohol dehydrogenases (ADHs) metabolizing diverse alcohols^13,14^. Strains of *P. putida* reportedly tolerate various organic solvents including alcohols^15–19^. *Pseudomonas* sp. NEEL19, originated from tea (*Camellia sinensis*) phylloplane shared the highest 16 rRNA gene sequence similarity with human-associated *P. juntendi* BML3^T^ (100%) and several solvent-tolerant *P. putida* (> 99.0%). NEEL19 grew in liquid culture supplemented with ethanol (0.5‒5%, v/v), 1-butanol (0.5%, v/v) and 1-octanol (0.5‒5%, v/v) as sole carbons^20^. IAA production on NEEL19 occurred when exposed to 1-octanol vapour, whereas no such response was found when treated with volatile ethanol^20^. This observation implied a differential impact of short- and long-chain alcohols on bacterial hormone secretion.

Here, we sequenced the NEEL19 genome to evaluate its genetic relatedness to *P. juntendi* BML3^T^ and explore comparatively the genetic machinery dedicated to alcohol metabolism and hormone (IAA and dopamine) production. We assessed the impact of direct contact with short- (ethanol) and long-chain (1-octanol) alcohols on bacterial hormone secretion in vitro. We further tested the impact of the major hormone IAA on wild-type *Drosophila melanogaster* gut-brain axis by conducting feeding experiments followed by behavioural studies. Quantitative proteomics was employed to gain a mechanistic understanding of impaired survivability and phenotype of *D. melanogaster*.

## Materials and methods

### Chemicals and reagents

Absolute ethanol and high purity (> 99.0%) 1-octanol were obtained from Fisher Scientific (Leicestershire, UK). IAA, dopamine, L-dopa and trp were purchased from Merck. Phenol red and AB dye were obtained from Invitrogen. HPLC-grade solvents were used for chromatographic analysis.

### Bacterial culturing, DNA extraction, genome sequencing, annotation and phylogeny

Strain NEEL19 is the lab isolate that originated from tea phylloplane^20^. Cells of NEEL19 were revived from − 80 °C and cultivated on nutrient agar (Himedia) for two days at 30 °C. The genomic DNA was extracted and purified using the Wizard DNA purification kit (Promega). The single-molecule real-time (SMRT) sequencing was performed on SMRT 1 M Cell v3 (PacBio, 101-531-000) with chemistry version 3.0 on PacBio Sequel sequencer by Genomics BioSci & Tech Co. Details of tools used for genome annotation are in Supplementary Information (SI). Closely related phylogenetic neighbours and genomic relatedness with established type strains of *Pseudomonas* were traced through TYGS^21^ and Orthologous Average Nucleotide Identity (OrthoANI)^22^, respectively. Sequences of ADHs from NEEL19 and reference strains were respectively retrieved from Rapid Annotation using Subsystem Technology (RAST)^23^ and UniProt^24^ and used for phylogenetic analysis as described in SI. The genome was visualized and manually annotated through the CGView server and Proksee^25,26^. Proteins involved in trp and hormone biosynthetic pathways were identified through RAST using sequences retrieved from UniProt.

### Impact of alcohol and trp inputs on cell density, metabolism, media acidity/alkalinity and hormone production in NEEL19

Cells of NEEL19 were cultivated in full-strength tryptic soy broth (Himedia) without and with 0.1% (w/v) trp input (TSB and TSB^W^, respectively). Cells were also grown in liquid minimal salt medium (M9) with 0.5% (v/v) ethanol and 0.5% 1-octanol (v/v) without trp (M9EtOH ad M9Oct, respectively) and with 0.1% (w/v) trp supplements (M9EtOH^W^ ad M9Oct^W^, respectively); 0.1% (w/v) trp-supplemented M9 (M9^W^) served as a control for M9^W^-based assays. Respective cell-free media were served as negative controls. Cells were cultivated aerobically at 37 °C for 3 days. Cell density, metabolism and media acidity/alkalinity were estimated using a spectrophotometer as specified in SI. Auxin and catecholamine hormones under various treatments were detected through thin-layer chromatography^27,28^ and quantified through calorimetry^29^ as described in SI.

### Detection of IAA production in gut isolates and influence of IAA intake on odor preference and motility of *Drosophila*

A method for qualitative determination of IAA secretion by gut bacteriome inhabiting male and female *D. melanogaster* is given in SI. A 5% yeast supplemented with 10 µg/ml of IAA was fed to male and female flies separately along with blue food dye (Three Leaves, GFC Pvt. Ltd.) as a feeding tracker. In the control group, male and female flies maintained separately were fed exclusively with 5% yeast and food dye. Survival, negative geotaxis, chemotaxis and phototaxis assays for IAA-fed male and female flies of *D. melanogaster* were carried out using standard protocols^30–32^ described in SI. Strains were identified based on full-length 16S rRNA gene sequence analysis as described earlier^33^.

### Fly head protein extraction and quantitative proteomics

The head homogenates (500 µL each) of IAA-fed and -unfed male and female flies of *D. melanogaster* were subjected to protein extraction by acetone precipitation (at − 20 °C overnight). The extracted protein pellet was dried and resuspended in 50 mM triethylammonium bicarbonate buffer and quantified using a bicinchoninic acid assay. The methods used for protein reduction, alkalyzation^34^, trypsin digestion^35^, purification and estimation are described in SI. Reverse phase fractionation for peptides was carried out using an in-house prepared stage tip column-based protocol (See SI). LC–MS/MS analysis by Data-Independent Acquisition (DIA) mode was carried out using an Orbitrap Fusion Tribrid mass spectrometer (Thermo Fischer Scientific, Bremen, Germany) connected to the Easy-nLC-1200 nanoflow liquid chromatography system (Thermo Scientific). The methods for Data-Dependent Acquisition (DDA) to generate the spectral library, wide-window DIA analysis, gas-phase fractionation (GPF) by narrow-window DIA^36–38^ are summarized in SI. *In-silico* spectral library for the FASTA sequences of *D. Melanogaster* proteins retrieved from the UniProt database were generated through DIA-NN software^39^.

### Statistical and bioinformatics analysis

Statistical significance (*p < 0.1, **p < 0.05, ***p < 0.01, ****p < 0.0001) for culture-based assays was estimated through t-test using GraphPad Prism version 6. Proteomics data were analysed statistically as described previously^39,40^. The protein abundances were normalized using median normalization and those with a fold-change value of ± 2 (*p* ≤ 0.05) were considered as differentially regulated. Gene ontology and pathway enrichment analysis were performed using the G:Profiler online tool^41^. Venn diagram was generated using the Venny online tool (https://bioinfogp.cnb.csic.es/tools/venny/). Sanky plot was generated using the RAWgraphs tool (https://doi.org/10.1145/3125571.3125585). Chemical structures were drawn using RCSB.org (https://www.rcsb.org/chemical-sketch). For all statistical and bioinformatics analysis and comparison, *p* ≤ 0.05 was considered significant, unless specified otherwise.

## Results

### Identification of NEEL19 as a new strain of clinically originated *P. juntendi*

**Figure 1:**
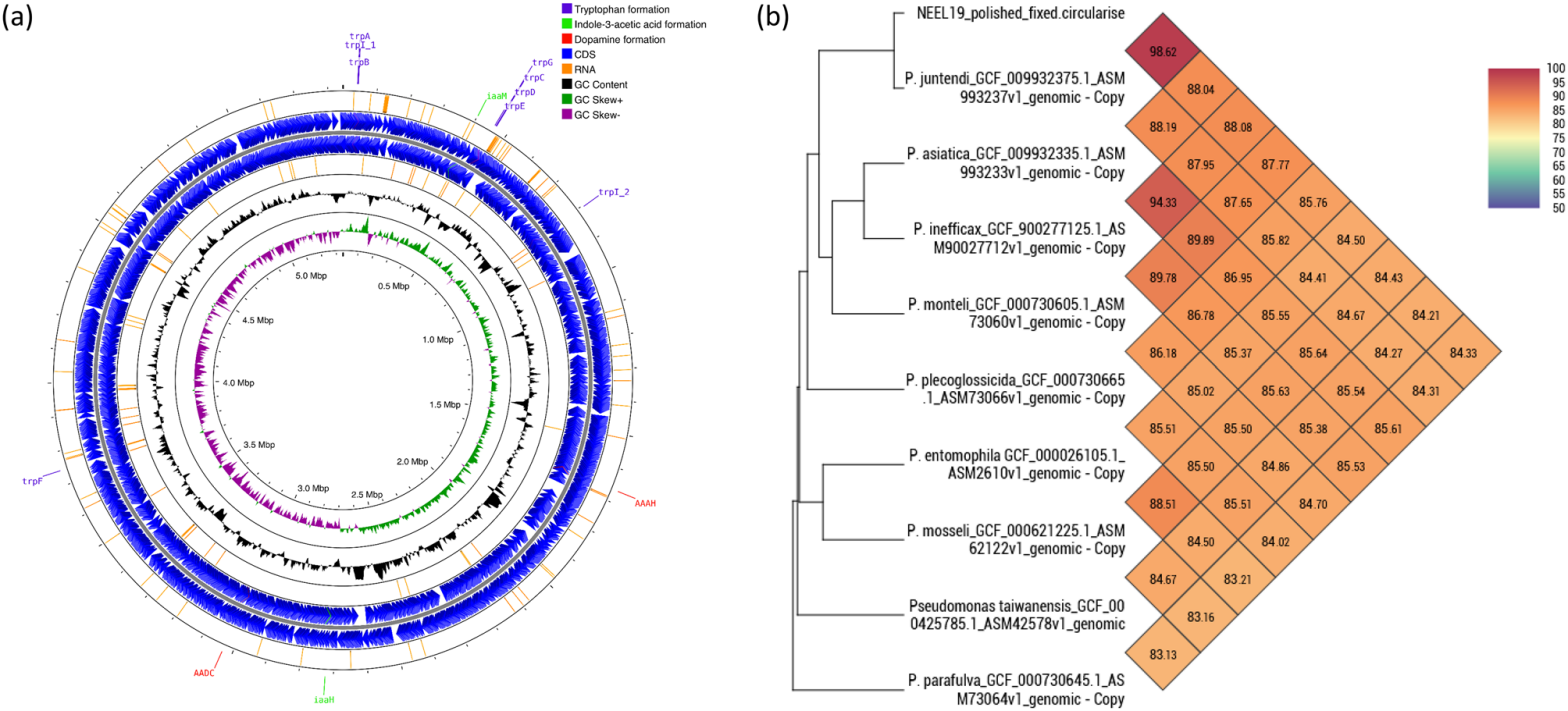
Circular genome map and OrthoANI heatmap obtained for *Pseudomonas juntendi* NEEL19. Circular genome plot showing the localization of genes involved in the alcohol metabolism, and biosynthesis of tryptophan and hormones (indole-3-acetic acid and dopamine) in NEEL19 (**a**). OrthoANI heatmap shows genomic relatedness of NEEL19 with the type strain of *P. juntendi* BML3^T^ and other closely related species of *Pseudomonas* based on EzBiocloud (**b**).

The circular map representing the complete genome sequence of NEEL19 is shown in Fig. 1a. Strain NEEL19 contained 5,344,505 bp, 62.66% GC content, one transfer-messenger RNA, 70 miscellaneous RNA, 22 rRNA, 4,815 genes, 4,646 coding sequences and 76 transfer RNAs. Phylogenetic analysis based on 16S rRNA and genome sequence at Type Strain Genome Server (TYGS) showed a close association of NEEL19 with many strains of *Pseudomonas* (Fig. S1a and b). In EzBiocloud, NEEL19 shared high pair-wise 16S rRNA gene sequence similarity with type strains such as *P. juntendi* BML3^T^ (100%)^42^, *P. asiatica* RYU5^T^ (99.8%)^43^, *P. monteilii* NBRC 103158^T^ (99.7%)^44^ and *P. mosselii* CIP 105259^T^ (99.4%)^45^ of clinical origins. In addition, it also shared high sequence similarity with ichtyopathogenic *P. plecoglossicida* NBRC 103162^T^ isolated from ayu (*Plecoglossus altivelis*) (99.7%)^46^, and entomopathogenic *P. entomophila* L48^T^ originated from female *D. melanogaster* (99.4%)^47^. NEEL19 shared 98.6% genomic relatedness with *P. juntendi* BML3^T^ while < 89.0% relatedness was recorded with other type strains as determined through OrthoANI (Fig. 1b). Thus, NEEL19 is considered to represent a new strain of *P. juntendi*.

### Co-occurrence of alcohol metabolism and hormone production in *P. juntendi*

**Figure 2:**
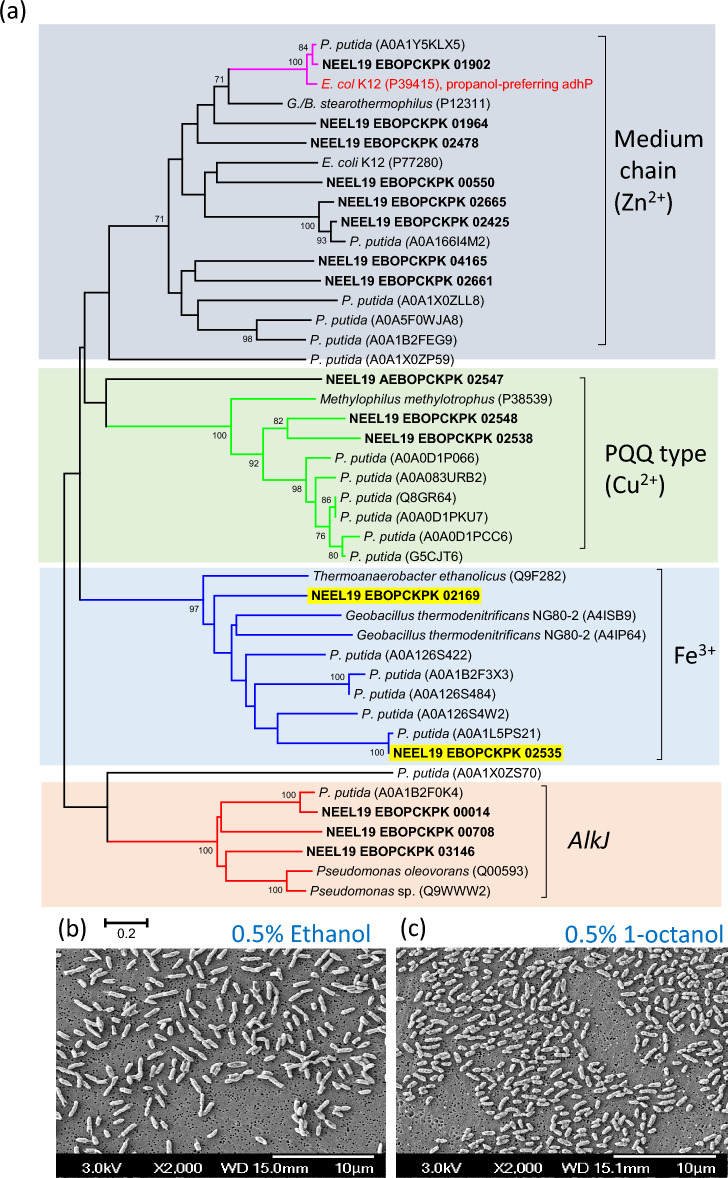
Phylogenetic analysis of alcohol metabolizing proteins detected in *P. juntendi* NEEL19 (**a**) and the pleomorphic response of the strain to ethanol and 1-octanol (**b**,**c**). Unrooted neighbour-joining phylogenetic tree generated for the amino acid sequences of various alcohol dehydrogenases detected in *P. juntendi* NEEL19 and related reference strains. Bootstrap values of > 70% are exclusively shown at branching points. Scale bar, 0.2 substitutions per position. Scanning electron microscopic images of *P. juntendi* NEEL19 cells grown in liquid basal medium supplemented with 0.5% (v/v) ethanol (**b**) and 0.5% (v/v) 1-octanol (**c**). Cells were grown at 37 °C for 3 days.

The NEEL19 genome was screened for genes dedicated to alcohol metabolism and hormone production. Genes encoding Fe^3+^ containing ADHs (*adhI* and *adhB*), Cu^2+^ containing quinoprotein dehydrogenases (*qedA*, n = 2; *qbdA*, n = 1), Zn^2+^ binding ADHs (n = 8) and acceptors of ADHs (*alkJ*, n = 3) were found in NEEL19 (Table S1). The phylogenetic analysis of proteins involved in alcohol degradation is shown in Fig. 2a. Morphological variation in NEEL19 as a function of alcohol treatments is shown in Fig. 2b,c. These data collectively indicated a genetic potential of diverse alcohol degradation in NEEL19.

**Figure 3:**
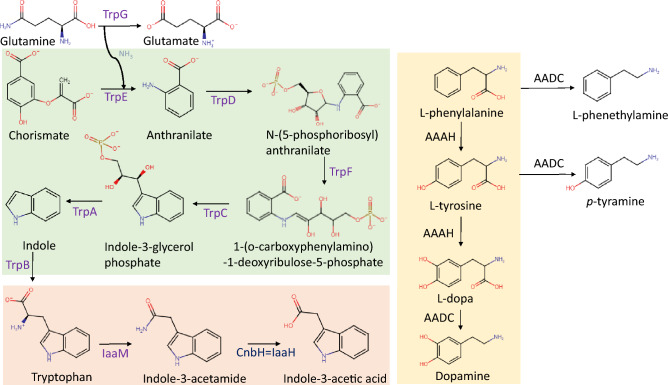
Pathways leading to the formation of tryptophan, indole-3-acetic acid, L-dopa and dopamine in NEEL19. Please Table S1 for the definition of enzymes and gene locus tags.

NEEL19 harboured a complete set of genes involved in trp biosynthesis that include trp synthase α and β chains (*trpA* and *trpB*), indole-3-glycerol phosphate synthase (*trpC*), anthranilate phosphoribosyltransferase (*trpD*), anthranilate synthase component 1 (*trpE*), N-(5'-phosphoribosyl)anthranilate isomerase (*trpF*) and anthranilate synthase component 2 (*trpG*) (Table S1. Figure 1a). Genes coding for trp 2-monooxygenase (*iaaM*) and 2-amino-5-chloromuconic acid deaminase (*cnbH*) sharing similarity with *iaaH*, involved in the formation of IAA from trp through indole-3-acetamide pathway^48^ were found. In addition, NEEL19 harboured genes coding for phenylalanine-4-hydroxylase (*phhA* = AAAH) and aromatic-l-amino-acid decarboxylase (*ddc* = AADC) respectively catalysing the formation of l-dopa and dopamine (Table S1). The pathways and corresponding enzymes involved in the formation of trp, IAA, L-dopa and dopamine in NEEL9 are depicted in Fig. 3 and gene details are summarized in Table S1. These data suggested the genomic potential for trp and multi-hormone production in NEEL19.

**Figure 4:**
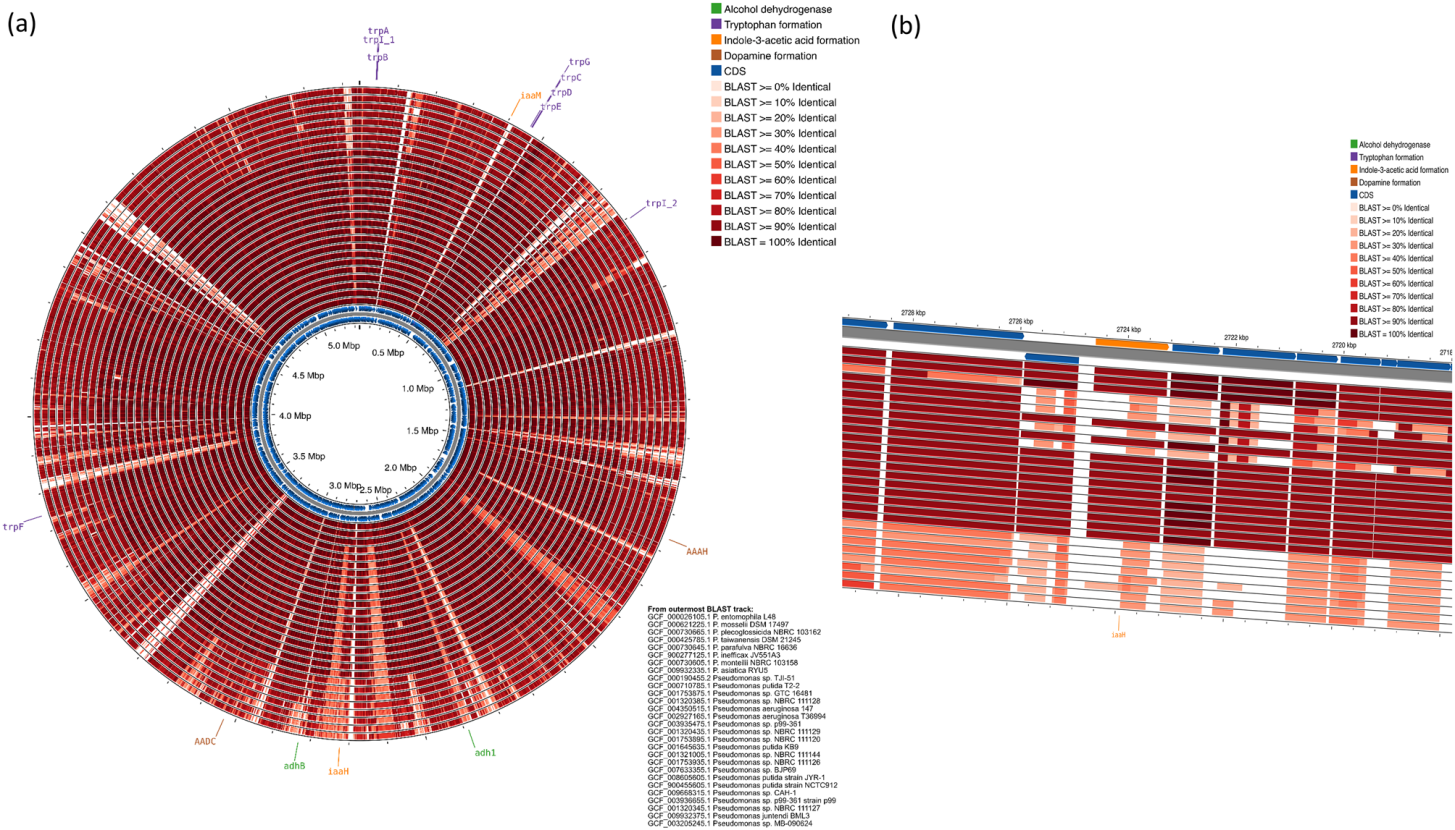
Circular genome map of NEEL19 (inner most dual counter-current blue rings) showing genome features, labelled genes of interest, and BLAST comparison results. The BLAST searches were conducted using CDS translations from NEEL19, which were compared to each translated comparison genome DNA sequence using tblastn and an E-value cutoff of 0.0001 (**a**). From the outermost ring to the innermost ring: BLAST results for 28 comparison genomes, NEEL19 genes on the forward strand, NEEL19 genes on the reverse strand. NEEL19 alcohol dehydrogenase genes and genes predicted to be involved in tryptophan formation, indole-3-acetic acid formation, and dopamine formation are labeled. The map was generated using Proksee (66, 67). The NCBI Genome Assembly accessions for the sequences used in the BLAST comparisons are as follows (ordered from the outside to the center): GCF_000026105.1, GCF_000621225.1, GCF_000730665.1, GCF_000425785.1, GCF_000730645.1, GCF_900277125.1, GCF_000730605.1, GCF_009932335.1, GCF_000190455.2, GCF_000710785.1, GCF_001753875.1, GCF_001320385.1, GCF_004350515.1, GCF_002927165.1, GCF_003935475.1, GCF_001320435.1, GCF_001753895.1, GCF_001645635.1, GCF_001321005.1, GCF_001753935.1, GCF_007633355.1, GCF_008605605.1, GCF_900455605.1, GCF_009668315.1, GCF_003936655.1, GCF_001320345.1, GCF_009932375.1, GCF_003205245.1. Enlarged view of the plot showing the heterogeneous distribution of putative *iaaH* in tested *Pseudomonas* (**b**).

Genetic signs for alcohol degradation and hormone production in *Pseudomonas* species closely related to NEEL19 were traced through comparative genomics. Strains and their target genes analysed are summarized in Table S2. Genes involved in alcohol metabolism and biosynthesis of trp, IAA, L-dopa and dopamine were detected consistently in *P. juntendi* cohort (Fig. 4a). It is noteworthy that *cnbH*, an amidase homologous to *iaaH,* were found to be present in a large proportion of *P. juntendi,* while it was missing non-*juntendi* genomes (Fig. 4b). Thus, alcohol metabolism and multi-hormone production predicted to co-occur in the majority *P. juntendi* and other related species including *P. entomophila.*

### Differential influence of ethanol and 1-octanol on cell growth, metabolism and hormone production in NEEL19

**Figure 5:**
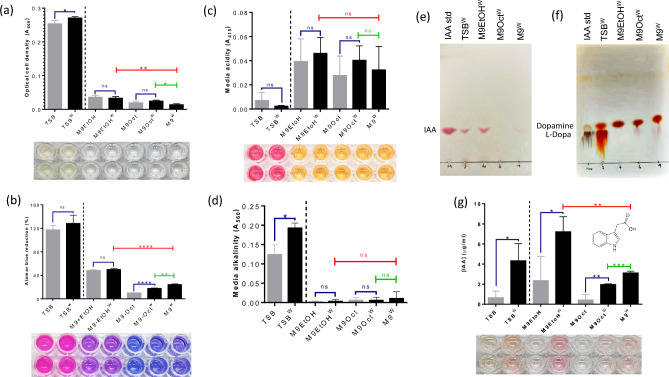
Impacts of short- and long-chain alcohols on cell growth, metabolic activity, media acidification/alkalization and IAA production in *P. juntendi* NEEL19. Optical cell density (**a**), alamar blue dye reduction (**b**), media acidification (**c**) and alkalization (**d**) are shown. Inset, a representative portion of the microplate. Thin layer chromatographs show the separation of hormones of auxin (**e**) and catecholamine (**f**). Colorimetric quantification of IAA produced by *P. juntendi* NEEL19 under various nutritional conditions. Error bar, mean (n = 4) ± s.d. *p < 0.1, **p < 0.05, ***p < 0.01, ****p < 0.0001; ns, non-significant. Statistical significances were determined using t-test; green horizontal line, M9^W^ vs. M9Oct^W^; red horizontal line, M9^W^ vs. M9EtOH^W^. Tryptophan-added treatments are shown as superscript W (single letter code for tryptophan). Treatment codes are defined in the section abbreviations.

The impact of TSB and alcohol-amended M9 media with/without trp on NEEL19 cells are as shown in Fig. 5a‒g. NEE19 exhibited significantly high cell density (*p* = 0.0486), media alkalization (*p* = 0140) and IAA production (*p* = 0.0260) in TSB^W^ as compared to TSB. While significantly high IAA (*p* = 0.412) was found in M9EtOH^W^, high metabolic activity (*p* < 0.0001) and IAA (*p* = 0.0081) were recorded in M9Oct^W^ as compared to their respective trp-lacking counterparts. M9Oct^W^ and M9EtOH^W^ showed significantly high OD (*p* = 0.0141 and 0.0044, respectively), metabolic activity (*p* = 0.0005 and 0.0093, respectively) and IAA (*p* = 0.0015 and < 0.0001, respectively) when compared to M9^W^. Thus, trp input was found to promote IAA secretion in NEEL19 under TSB and ethanol-amended M9 treatments. Short- and long-chain alcohols differentially influence cell density, metabolism and IAA secretion in NEEL19.

Thin layer chromatography (TLC) was carried out to visualize hormones produced by NEEL19 in the combined presence of respective alcohol and trp. IAA secretion was found to be high in trp-supplemented ethanol treatments (Fig. 5e) as compared to trp-supplemented 1-octanol counterpart, whereas L-dopa secretion appeared to be relatively high in the latter (Fig. 5f). High amounts of IAA and L-dopa secretion was also detected in trp-supplemented TSB. Colorimetric analysis showed significantly high (*p* < 0.01) IAA secretion by NEEL19 when provided with trp-supplemented ethanol as compared to TSB control (Fig. 5g). These data indicated a booster role played by ethanol on IAA secretion by NEEL19 when provided with exogenous trp.

**Figure 6:**
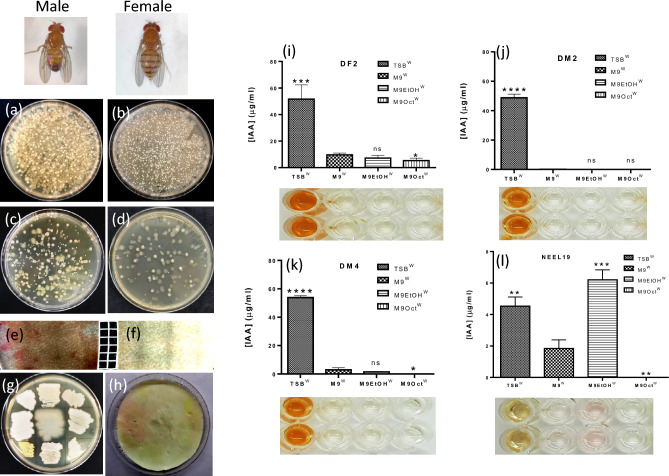
Characterization of *Drosophila* gut microbiota for the production of IAA. *Drosophila* gut microbiota isolated from male and female flies plated without (**a**,**b**) and with dilution (**c**,**d**). Filter plate blot assay stained with Salkowski reagent showing traces of IAA on diluted spread plate agar containing gut microbiota of male and female flies (**e**,**f**). Purified colonies from male and female flies (**g**) and filter paper mount sprayed with Salkowski’s reagent. Quantification of iAA produced by a bacteria originated from male fly (**i**), female fly (**j**,**k**) and NEEL19 reference (**l**) under two different alcohol supplements. Error bar, mean (n = 4) ± s.d. *p < 0.1, **p < 0.05, ***p < 0.01, ****p < 0.0001; ns, non-significant. Statistical significances versus M9^W^ were determined using t-test. Tryptophan-added treatments are shown as superscript W (single letter code for tryptophan). Treatment codes are defined in the section abbreviations.

NEEL19 shared highest genomic relatedness to *P. juntendi* BML3^T^ (98.6%) isolated from the sputum sample of a hospitalized patient^42^. *P. juntendi* shows a human association as the majority of strains have been isolated from clinical settings, particularly in Japan (Table S2). NEEL19 shared 84.5% genome relatedness with entomopathogenic *P. entomophila* L48^T^ isolated from female *D. melanogaster*^47^. Therefore, the gut system of *Drosophila* was screened for possible colonization of IAA-producing bacteria. We found the ubiquitous occurrence of IAA producers in both male and female fly gut systems (Fig. 6a‒f). Isolates were purified (Fig. 6g,h), and subjected to IAA quantification after growing them in ethanol, 1-octanol and TSB under trp-supplement. Gut isolates were found to produce > tenfold IAA as compared to *P. juntendi* NEEL19. NEEL19 and strains (DF2, DM2 and DM4) isolated from *Drosophila* gut showed significant (*p* < 0.0044) IAA secretion in TSB^W^ as compared to M9^W^ (Fig. 6i‒l). However, while ethanol stimulated IAA secretion (*p* = 0.0008) exclusively in NEEL19, 1-octanol suppressed IAA secretion (*p* < 0.0276) in all tested strains including NEEL19. IAA-producing gut isolates of *D. melanogaster* were identified to be different strains of *Providencia rettgeri* based on full-length 16S rRNA gene sequence analysis. However, none of the *Providencia* strains responded positively to ethanol treatment. Further optimization of culture conditions is required to isolate alcohol-responsive strains from the *Drosophila* gut.

### IAA consumption alters odor preference and motility in *D. melanogaster*

**Figure 7:**
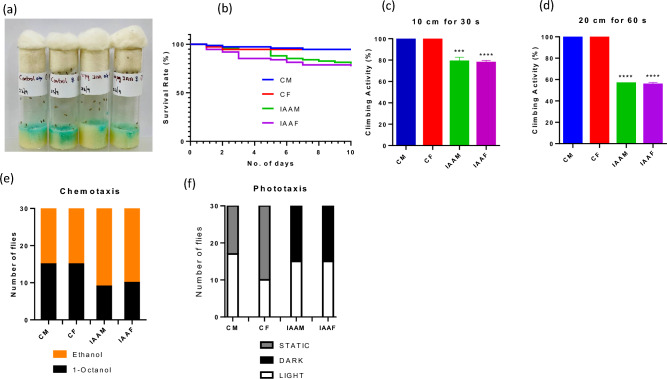
Influence of IAA on the survivability and motility in *Drosophila melanogaster*. Flies were fed with indole-3-acetic acid over a period of ten days Culture setup (**a**), survival rate (**b**), short-distance climbing (**c**), long-distance climbing (**d**), chemotaxis (**e**) and phototaxis (**f**) are shown. Error bar, mean (n = 3) ± s.d. Statistical significances were determined using t-test. ***p < 0.01, ****p < 0.0001; ns, non-significant. CM and CF, IAA-unfed control male and female, respectively. IAAM and IAAF, IAA-fed test male and female, respectively.

*Drosophila melanogaster* was used as a model eukaryote to test the impact of high purity IAA on gut-brain axis. Male (IAAM) and female (IAAF) flies were fed with IAA (10 μg mL^−1^, Fig. 7a) and probed for their survivability and motility while keeping respective IAA-unfed flies (control male (CM) and control female (CF), respectively). IAAM and IAAF showed declined survivability (Fig. 7b) and significantly (*p* < 0.01) low motility (Fig. 7c,d) besides exhibiting altered chemotaxis (Fig. 7e) and phototaxis (Fig. 7f) as compared to their respective IAA-unfed controls (CM and CF). IAA-driven shift towards ethanol is interesting since it would constitute a positive feedback loop that could be of significance in addiction processes. Taken together, these data indicated that IAA influences the survivability, odor preference and motility in *D. melanogaster*.

**Figure 8:**
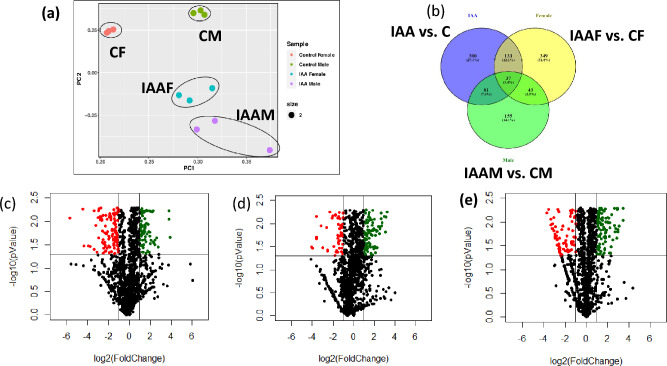
Plots showing protein distribution (**a**,**b**) and differential regulation (**c**‒**e**) in *Drosophila melanogaster* as a function of IAA exposure. Principal component analysis plot showing the clustering of technical triplicates under different experimental conditions based on protein abundance in respective replicates (**a**). Venn’s diagram showing the overlap of differentially regulated proteins (Fold change ± 2; *p* < 0.05) in flies treated with and without IAA (IAA vs. control), female flies with and without IAA (IAAF vs CF) and male flies with and without IAA (IAAM vs. CM) (**b**). Each experimental condition had three technical triplicates that clustered together. Technical triplicates belonging to different groups clustered separately showing that while the protein abundance variation was minimal among the technical triplicates, the experimental conditions showed variation among each other. Volcano plots showing differentially regulated head proteins in IAA vs. control (**c**), IAAM vs. CM (**d**) and IAAF vs. CF (**e**). Green dots depict upregulated and red dots depict the downregulated proteins.

IAA-driven alterations in neuronal and neuromuscular features were presumed to alter odor preference and motility in *D. melanogaster.* Therefore, total brain proteomes of IAA-fed and IAA-unfed *D. melanogaster* were extracted and subjected to high-resolution mass spectrometry to understand the protein-level changes that occurred as a function of IAA intake while their IAA unfed counterparts served as controls. The library used for protein identification consisted of both the Experimental Spectral Library (ESL) and the Predicted Spectral Library (PSL). ESL comprised of a library of spectra derived from DDA and GPF data, while PSL comprised a library of spectra predicted from the reference proteome of *D. melanogaster* (Supplementary Methods)*.* The spectral library search resulted in the identification of 4331 non-redundant, quantifiable proteins. These proteins were identified with at least 2 peptides which were found in minimal 2 technical replicates in each of the experimental conditions. Among these, 219 proteins (5.1% of total identification) were identified from both ESL (Table S3) and PSL (Table S4). Further, 63 proteins (1.5%) were identified in ESL alone, and 4049 proteins (93.5%) were identified only in PSL. These proteins were taken further to identify Differentially Regulated Proteins (DRPs). Principle component analysis showed the sex-wise and treatment-wise segregation of proteins among technical triplicates in all 4 experimental conditions, demonstrating sufficient variation among the experimental conditions and similarity among the technical triplicates (Fig. 8a).

Further to understand the impacts of IAA on *D. melanogaster* brain proteins, we conducted differential regulation analysis, where we considered both fold change values (IAA-fed/IAA-unfed ± 2) and *p* value from the student’s t-test (*p* < 0.05) for picking DRPs. DRPs were analysed in 3 sets of conditions: a) IAA-fed test cohort (both male and female) versus IAA-unfed control cohort (both male and female) (IAA/C); b) IAA-fed males versus IAA-unfed control males (IAAM/CM); (c), IAA-fed females versus IAA-unfed control females (IAAF/CF). We identified a total of 1098 DRPs (Table S5‒S7; Fig. S2). Among these, 37 proteins were differentially regulated in all three experimental conditions (Fig. 8b). Also, 218 proteins were upregulated and 333 proteins were downregulated due to IAA treatment. In IAAM, 171 proteins were upregulated and 145 proteins were downregulated. In IAAF, 364 proteins were upregulated and 198 proteins were downregulated (Fig. 8c‒e). Thus, female flies appeared to be more responsive to IAA treatment as compared to the males.

**Figure 9:**
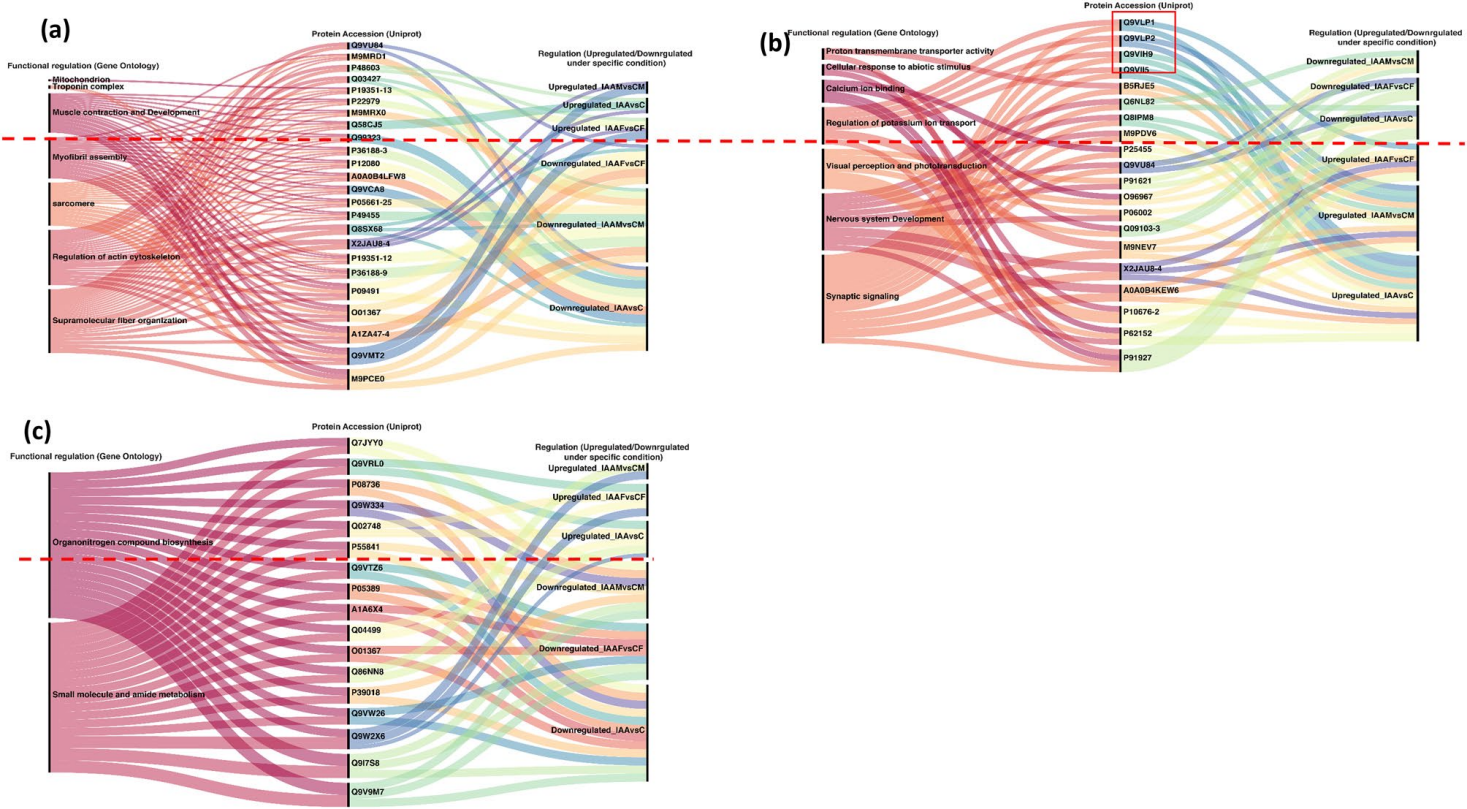
Sankey’s diagrams depicting the impacts of IAA on head proteins governing neuromuscular features and nitrogen metabolism in *Drosophila melanogaster*. Short-listed differentially regulated head proteins involved in structural and functional aspects of muscle (**a**), nervous system (**b**) and nitrogen metabolism (**c**) in flies with and without IAA (IAA vs. C), in male flies with and without IAA (IAAF vs. CF), and female flies with and without IAA (IAAM vs. CM) are shown.

DRPs involved in structural and functional neuromuscular aspects and protein synthesis in *Drosophila* were analysed. In agreement with our prediction, the DRPs represented the impact of IAA on the muscle proteins, nervous system and nitrogen metabolism as shown in Fig. 9a‒c and listed in Table S8. Some of the important muscle proteins and their regulation under IAA treatment are as follows. IAA treatment upregulated F-actin-capping protein subunit beta (cpb), isoform 13 of Troponin T, GH01093p, isoform 4 of protein nervous wreck (nwk) and GEO07854p1 (Fig. 9a). In contrast, IAA downregulated drebrin-like protein (Abp1), muscle-specific protein (Msp300), lamin-C (LamC), heat shock protein 67B3, limpet isoform J (Lmpt), myosin heavy chain non-muscle (zip), integrin alpha-PS2, thin isoform E, ankyrin repeat and KH domain-containing protein (mask), isoform L of myosin heavy chain of muscle (Mhc), tropomyosin-1 isoforms 33/34 (Tm1), CTTNBP2 N-terminal-like protein (Naus), isoform 12 of troponin T skeletal muscle, isoforms 2 and 8 of troponin I (wupA), tropomyosin-2, protein held out wings (how), isoform E of PDZ, LIM domain protein (Zasp52) and phosphoglycerate kinase (Pgk).

Similarly, several nervous system proteins were also dysregulated. Notably, IAA treatment upregulated five distinct protein quiver (promotes sleep), complexin (cpx), Fife isoform B, tumor necrosis factor receptor superfamily member 6, opsin Rh1 (ninaE), amphiphysin isoform B, neither inactivation nor afterpotential protein C (ninaC) and calmodulin (Cam) (Fig. 9b). In contrast, it downregulated protein lin-7 homolog (veli), protein still life isoform SIF type 1 (sif), drosocrystallin (Crys) and eye-specific diacylglycerol kinase (retinal degeneration A protein, rdgA).

Other important classes of dysregulated proteins were involved in nitrogen and energy metabolisms. IAA treatment upregulated cytochrome c1, isoform A, eukaryotic initiation factor 4A, proline dehydrogenase 1 mitochondrial (sluggish protein, slgA), COX5B and ATP synthase delta subunit isoform A (Fig. 9c). In contrast, IAA treatment downregulated ribosomal protein (RpS30), actin muscle-type A1, small ribosomal subunit protein eS28 and eS19A (RpS28b and RpS19a), large ribosomal subunit protein eL14 and P2 (RpL14 and RpLP2), phosphomannomutase, IP17216p, ornithine aminotransferase mitochondrial and 60S ribosomal protein L21.

## Discussion

Clinical association of *P. juntendi*^42^, its drug-resistant attributes^49,50^, differential response to the vapours of short- and long-chain alcohols^20^ and close phylogenetic association with entomo- and ichtyo-pathogenic *Pseudomonas* species^46,47^ promoted a detailed investigation on NEEL19. Detection of an array of genes encoding ADHs validated diverse alcohol metabolism reported earlier in NEEL19^20^. Phylogenetic analysis of 16 ADHs detected in NEEL19 resulted in four discrete clusters in which two ADHs (EBOPCKPK 02169 and EBOPCKPK 02535) formed lineages within the clade that accommodated long-chain alkyl ADHs of *Geobacillus thermodenitrificans* particularly active against ethanol (A4IP64) and 1-octanol (A4ISB9)^14^. SEM analysis showed the pleomorphic nature of NEEL19, where ethanol-treated cells were relatively larger with tapered end as compared to that of 1-octanol-treated cells, suggesting distinct impacts of these alcohols on cell phenotype. Detection of distinct alcohol-metabolizing genes are significant since the majority of *P. juntendi* genome exhibit clinical origins and may point towards its adaptive strategy to counter alcohol exposure (Table S2).

*Pseudomonas* strains are known to produce IAA^48^ and indoleamines such as serotonin and melatonin^51,52^; however, no report exists on the production of dopamine by *Pseudomonas*. Thus, dopamine formation detected in NEEL19 is significant. Bacterial species such as *P. entomophila*^47^, *Erwinia carotovorum*^53^*, Acetobacter fabarum* and *Lactobacillus brevis*^54^ were found to be associated with *D. melanogaster;* however, information doesn’t exist either on their IAA-producing or alcohol utilization traits. IAA-producing genes were not found in the type strains of *E. carotovorum, A. fabarum* and *L. brevis.* Thus, the detection of IAA (and dopamine) genes in *Pseudomonas entomophila* L48^T^ is noteworthy since this strain reportedly originates from *Drosophila*^47^. We tried to isolate IAA-producing bacterial strains directly from the *Drosophila* gut and found *Providencia ruttgeri* as a potential IAA producer. The representatives of *Providencia* are known to be pathogenic to *Drosophila*^55^. Interestingly, *Providencia* strains produced > tenfold IAA than that of NEEL19; however, they were found to be irresponsive to alcohol treatments possibly due to the lack of genes encoding ADHs. In contrast, indole-3-acetamide pathway^48^ and ADHs detected in NEEL19 explain the positive reaction of this strain to ethanol. Comparative genomics revealed the ubiquitous occurrence of genes involved in the biosynthesis of ADHs, trp, IAA, L-dopa and dopamine in *P. juntendi* and other related species. Therefore, the influence of alcohols on the indoleamine and catecholamine hormone formation in *P. juntendi* warrants further investigation.

Studies performed elsewhere on auxin-inducible degradation system or genotoxicity assessment in *D. melanogaster* used IAA concentrations of 1‒20 mM^56,57^ and a synthetic auxin analog (1-naphthalene acetic acid) concentrations of 0‒400 mM^58^ for feeding the flies/larvae. In this study, high purity (> 99.0%) IAA was used to assess the impacts on odor preference and motility in wild-type *D. melanogaster.* The IAA dose (10 μg mL^−1^ =  ~ 60 mM) and duration of feeding were optimized based on bacterial IAA production range^20^ suitable to elicit visible changes in *Drosophila* phenotype without breaching the threshold lethality (LD50). After ten days of feeding, IAA-fed flies exhibited declined survivability and altered motility (chemotaxis and phototaxis) as compared to respective controls. IAA was found to upregulate the protein nervous wreck (isoform 4), a *Drosophila* homolog of the human srGAP3/MEGAP protein associated with mental retardation, proposed to control synapse morphology by regulating actin dynamics downstream of growth signals in presynaptic terminals^59,60^. However, IAA treatment downregulated msp300, a striated muscle-specific protein exclusively involved in the anchoring of the myonuclei to the core actomyosin fibrillar compartment^61^. IAA depleted LamC, which supports the formation of central spindle microtubules essential for cytokinesis in male *Drosophila* during meiosis^62^. IAA downregulated Hsp67Bc, which is the closest functional ortholog of human heat shock protein HSPB8, whose impairment or loss of function is thought to accelerate the progression and/or severity of folding diseases such as polyglutamine disorder spinocerebellar ataxia 3 and peripheral neuropathies^63^. IAA downregulated the myosin heavy chain, which is a motor protein of muscle thick filaments, governing different physiological and ultrastructural characteristics of various muscle types in *D. melanogaster*^64^, and Nausicaa (Naus), which is hypothesized to enhance branch nucleation and junction stability by slowing down cortactin's disassociation from Arp2/3 nucleated branch junctions^65^.

IAA was found to influence troponin-tropomyosin complex (TTC), which acts through a series of Ca^2+^-dependent conformational changes controlling the actin-myosin interactions and muscle contraction^66^. IAA downregulated isoforms 2 and 8 of troponin 1 of *D. melanogaster*. Aberration in troponin I, which is one of the principal regulatory proteins associated with skeletal muscle thin filaments, is known to cause grossly abnormal muscle myofibrils^67^. Isoforms of troponin I play a functional role in the muscle and nervous system during development and the mature activity of *D. melanogaster*^68^. IAA suppressed troponin-T, whose impairment rendered myofibrillar abnormalities in *D. melanogaster*^69^, and hampered tropomyosin-2, a unit of TTC proposed to act as a regulator of motor systems required to maintain nuclear integrity and apico-basal polarity^66^. IAA down-regulated protein held out wings (how), which controls the expression of unidentified mRNAs coding for proteins essential to cardiac and muscular activity^70^. IAA down-regulated integrin alpha-PS2, which is involved in muscle attachment during early development^71^, and Zasp, which localizes to integrin adhesion sites and its depletion disrupts integrin adhesion sites in *D. melanogaster*^72^. Taken together, these altered protein profiles could explain the hampered motility in IAA-fed flies.

IAA treatment hampered Abp1, which governs cortical actin dynamics and plays a critical role in cortical Arp2/3-mediated actin nucleation during sensory organ development^73^, and the establishment of synaptic bouton and branching of neuromuscular junctions^74^. IAA downregulated heavy chain of myosin (non-muscle, zip), which plays a key role in the auditory organ of *Drosophila*^75^, and MASK, which genetically interacts with receptor kinase signaling pathways^76^ and plays a role in muscle development^77^. IAA is likely to hamper visual perception of *Drosophila* as loss of MASK function generates phenotypes of compromised photoreceptor differentiation, cell survival and proliferation in *D. melanogaster*^76^. IAA downregulated still-life protein (sif), orthologous to human dynamin binding protein involved in cataracts, regulating synaptic differentiation through the organization of the actin cytoskeleton by activating Rho-like GTPases^78,79^. Knockdown of sif in *Drosophila* hampered the development of cells that secrete the lens material and exhibited diminished electroretinography amplitudes reflecting an aberrant phototransduction cascade^78^. Similarly, IAA treatment downregulated drosocrystallin, a major glycoprotein of the *D. melanogaster* corneal lens^80^, and eye-specific diacylglycerol kinase (retinal degeneration A protein, rdgA), the absence of which leads to rhabdomere degeneration due to defective phospholipid turnover^81^. Taken together, these data suggested IAA-driven impairment in audio-visual perception in *D. melanogaster*.

IAA down-regulated Letm1, a mitochondrial proton/calcium antiporter that mediates proton-dependent Ca^2+^ efflux from mitochondrion^82^, and ornithine aminotransferase involved in the transformation of ornithine to non-essential amino acid proline^83^. However, IAA treatment upregulated mitochondrial proline dehydrogenase 1 (slgA), an enzyme catalysing the conversion of proline to glutamate^84^. IAA suppressed Lmpt, possibly involved immune response^85^, smaller subunits of ribosomal proteins such as RpS19a^86,87^, RpS28b^86,88^, RpS30^88^, and larger subunits of proteins such as RpLP2^86^, RpL14^89^ and RpL21^87,88^. In addition, IAA downregulated phosphomannomutase 2 (pmm2), whose impairment causes congenital disorder of glycosylation resulting in severe neurological impairment and shortened lifespan^90^, and phosphoglycerate kinase involved in substrate-level ATP synthesis.

In summary, we identified IAA- and dopamine-producing capabilities in *P. juntendi* and established a linkage between bacterial ethanol utilization and IAA production. The ethanol-driven IAA secretion in *P. juntendi* occurring in the presence of trp possibly hints at previously unknown chemical crosstalk occurring between microbes and eukaryotic hosts. IAA was further found to play a key role in *D. melanogaster* gut-brain axis by influencing odor preference, motility and survivability. Brain proteomics provided evidence for IAA-driven neuronal, neuromuscular and audio-visual impairment in *D. melanogaster* and hinted at altered molecular aspects including protein synthesis and energy metabolism that underpin the visible changes in fly behaviour. Experiments are awaited to decode the impact of gut microbial dysbiosis on IAA production and to elucidate the exact role played by differentially regulated *Drosophila* proteins that determine the possible aberrant phenotypes including the sleep (quiver)^91^. Similarly, microbial dysbiosis leading to the possible dominance of IAA producers like *P. juntendi* in the gut system of alcoholics warrants investigations as these individuals may face a high risk of developing neurological disorders and/or cognitive impairment. Functional metagenomics may shed more light on the abundance, diversity and species richness of potential alcohol-responsive indole-, indoleamine- and catecholamine-producing bacteria inhabiting *Drosophila* gut for further in-depth analysis.

### Supplementary Information

Supplementary Information 1.

Supplementary Information 2.

Supplementary Information 3.

Supplementary Information 4.

## Data Availability

Complete genome sequence data of *Pseudomonas juntendi* NEEL19 is deposited at NCBI (https://www.ncbi.nlm.nih.gov/search/all/?term=CP081491) under BioProject No. PRJNA753930, BioSample No. SAMN20717984 and accession No. CP081491. The Proteomics raw data is deposited at ProteomeXchange Consortium (http://proteomecentral.proteomexchange.org) via the PRIDE partner repository with the data set identifier PXD044924.

## Abbreviations

ADHs: Alcohol dehydrogenases
CF: Control (IAA-unfed) female
CKD: Chronic kidney disease
CM: Control (IAA-unfed) male
DDA: Data-dependent acquisition
DIA: Data-independent acquisition
DRPs: Differentially regulated proteins
ESL: Experimental spectral library
GPF: Gas-phase fractionation
IAA: Indole-3-acetic acid
IAAF: IAA-fed female flies
IAAM: IAA-fed male flies
M9: Minimal salt medium
M9^W^: M9 with 0.1% (w/v) tryptophan supplement
M9EtOH: M9 with 0.5% (v/v) ethanol
M9EtOH^W^: M9EtOH with 0.1% (w/v) trp supplement
M9Oct: M9 with 0.5% (v/v) 1-octanol
M9Oct^W^: M9Oct with 0.1% (w/v) trp supplement
OrthoANI: Orthologous average nucleotide identity
PSL: Predicted spectral library
RAST: Rapid annotation using subsystem technology
SMRT: Single-molecule real-time
TLC: Thin layer chromatography
Trp: Tryptophan = W
TSB: Tryptic soy broth
TSB^W^: TSB with 0.1% (w/v) trp supplement
TTC: Troponin-tropomyosin complex
TYGS: Type strain genome server
