# Supplementary material for: Comprehensive insights into the impact of bacterial indole-3-acetic acid on sensory preferences in Drosophila melanogaster
Source: Sci Rep. 2024 Apr 9;14:8311. doi: 10.1038/s41598-024-58829-7 (PMC11003987; doi:10.1038/s41598-024-58829-7)
Supplement: Supplementary file 1 — Supplementary Information 1. [file 41598_2024_58829_MOESM1_ESM.docx]

**Supporting Information**

**Contents**

**SI methods**

**Supplementary Tables S1‒S3, S5‒S8**

**Supplementary Figures S1‒S2, S4‒S5**

**SI Methods**

**Cell culture and genome sequencing of *P. juntendi* NEEL19**

Genomic DNA (1 μg) was sheared by Covaris g-TUBE (Covaris, 520079) and purified via AMPure PB beads (PacBio, 100-265-900). The sheared and purified DNA fragments were used as templates to prepare the SMRTbell library via SMRTbell template prep kit 1.0 (PacBio, 100-991-900), according to the manufacturer’s instructions. After damage fixing and end repairing, the A-tailed inserts were ligated with barcoded overhang adapters and removed small insert SMRTbell templates by performing size selection with the BluePippin System. The sequencing was performed on SMRT 1M Cell v3 (PacBio, 101-531-000) with chemistry version 3.0 on PacBio Sequel sequencer by Genomics BioSci & Tech Co. A primary filtering analysis was performed on the Sequel system followed by a secondary analysis using the SMRT analysis pipeline version 9.0. The hifi reads were generated by subreads with 3 passes with a predicted accuracy of 0.99 and were assembled by hifiasm v0.8. SSPACE-long v1.1 and PBJelly v15.8.24 were applied for scaffolding and gap closing, respectively. The genome was polished by Arrow v2.3.3 and circularized by circulator v1.5.5. QUAST v4.6.3 was used for evaluating the quality of the assembled genome.

**Genome annotation and phylogenomics for *P. juntendi* NEEL19**

Prokka v1.13 was used for finding the bacteria origin of replication (ori), followed by downstream gene/CDS/RNA sequence prediction using the blast method against the UniProt/SwissProt bacterial database (1). Genes coding for transporters, virulence factors, drug targets and antibiotic resistance were screened through TCDB (2), PATRIC/VFDB 2016 (3, 4), TTD/DrugBank 4.0 (5, 6) and CARD (v3.0.5) (7). Resistance Gene Identifier (RGI) v5.1.0 was used to predict resistomes from protein or nucleotide data based on homology and SNP models. AMR phenotype predictions were derived using custom-built AdaBoost (adaptive boosting) machine learning classifiers at PATRIC (8, 9). Additional computational and bioinformatics analyses were done through COG (10), GO (11) and KEGG (12) databases.

**Phylogenetic reconstruction of genes of interest from *P. juntendi* NEEL19**

Amino acid sequences of alcohol dehydrogenases were subjected to multiple alignments using Clustal_X (13) with respective reference proteins retrieved from the UniProt database after blast search. Neighbor–joining phylogenetic tree (14) was generated through MEGA 5 (15) using the distance matrix method (Kimura two-parameter model; 16). The robustness of tree topology was evaluated through the bootstrap resampling method after 1,000 replications (17).

**Reconstruction of trp and hormone biosynthetic pathways in *P. juntendi* NEEL19**

Amino acid sequences of alcohol dehydrogenases, and proteins involved in the biosynthesis of trp, IAA, L-dopa and dopamine. *Pseudomonas juntendi* NEEL19 genome was uploaded at the RAST server and screened for the genes encoding proteins involved in the biosynthesis of trp, IAA and dopamine using reference amino acid sequences of corresponding proteins retrieved from the UniProt database.

**Culture experiments *in vitro* for *P. juntendi* NEEL19 (optical cell density, alamar blue, acidity and alkalinity)**

Cell suspensions were transferred to a 96-well microplate for OD_600_ measurements in a microplate reader (Biochrom [Asys UVM 340](https://www.google.com.tw/url?sa=t&rct=j&q=&esrc=s&source=web&cd=3&cad=rja&uact=8&ved=0ahUKEwjPgMTu_-PSAhUHFZQKHTfzAIoQFggkMAI&url=http%3A%2F%2Fwww.biocompare.com%2FProduct-Reviews%2F41425-Asys-UVM-340-Microplate-Reader-From-Biochrom%2F&usg=AFQjCNES1XEG7oQbiHDwTOFo5pRHvJdLsA&sig2=LkGNGrxIbbP5mRpktpRAHQ)). AB dye reduction (%) was estimated according to the manufacturer’s protocol by introducing 10% (v/v) AB to culture suspension in a microplate, followed by reading the plates at 570 and 600 nm. Media acidity/alkalinity were determined in cell-free culture supernatants using microplates. Acidity and alkalinity were probed by adding 10% (v/v) phenol red to the supernatant, and reading the plates at 415 and 560 nm, respectively.

**Detection of hormone (auxin and catecholamine) secretion by *P. juntendi* NEEL19 using thin-layer chromatography**

IAA and dopamine production in full-strength tryptic soy broth (TSB) without and with 0.1% (w/v) trp supplement (TSB and TSB^W^, respectively), M9 media containing 0.5% (v/v) absolute ethanol without and with 0.1% (w/v) trp (M9EtOH and M9EtOH^W^, respectively), M9 media containing 0.5% (v/v) 1-octanol without and with 0.1% (w/v) trp (M9Oct and M9Oct^W^, respectively) and M9 media supplemented exclusively with 0.1% (w/v) trp (M9^W^). Thin layer chromatography was performed to detect IAA and dopamine in the spent culture media of *P. juntendi* NEEL19 after 72 h of incubation. Cell-free supernatants were concentrated with lyophilization, reconstituted using DDW and loaded onto TLC plate (20 × 20 cm, Merck) and developed using a mixture of chloroform, ethyl acetate and formic acid (77:22:2, v/v) as a mobile phase for the detection of IAA. Spots of IAA was visualized and identified by spraying the TLC plates with Salkowski’s reagent (35% HClO_4_ solution containing 10 mM FeCl_3_) with reference to authentic standard. A mixture of ethyl acetate: n-propanol: water: acetic acid (19:2:10:1, v/v) was used as a mobile phase for the separation of L-dopa and dopamine. Spots were visualized and identified by spraying the TLC plates with butanolic 0.3% (w/v) ninhydrin with reference to authentic standards.

**Quantitative determination of IAA secreted by *P. juntendi* NEEL19 under various nutritional conditions**

Cell-free supernatants were screened for IAA production was assessed colorimetrically with the following modifications: One-fold supernatant was mixed with four-fold Salkowski reagent in a 96-well microplate, incubated for 15 min at room temperature and read at 530 nm. IAA was quantified against the standard curve plotted for the IAA standard.

**Qualitative determination of IAA secretion by *Drosophila melanogaster* gut microbiome**

Gut lysates of male and female flies (n=10 each) without and with five-fold dilution were plated on to tryptic soy agar plates and incubated overnight at 30 °C under darkness. A sterile filter paper was mounted on the actively growing cells, briefly air-dried and stained with Salkowski reagent to visualize the secreted IAA spots. Filter paper mounted on plain TSA was used as a control. Colonies were isolated and purified for quantitative estimation using Salkowski reagent.

**Maintenance of *Drosophila melanogaster* and survival assay**

Three-days-old wild-type *D. melanogaster* (Canton-S) strain was used. Flies were reared at 24±1 ℃ with a relative humidity of 65–70% and 12:12 h light–dark cycle on a standard cream of wheat agar media containing agar, wheat flour, jaggery and propionic acid. Additional yeast suspension was provided for the healthy growth of the organism.

To investigate the effect of IAA on mortality rate, a survival/toxicity analysis assay was conducted following the previously described protocol. A 5% yeast supplemented with 10 µg/ml of IAA (final concentration) was fed to flies along with blue food dye (Three Leaves, GFC Pvt. Ltd.) as a feeding tracker. Filter-sterilized IAA, blue dye and yeast were mixed and added on top of the media during the tube preparation. Feeding was confirmed by visualizing the blue color in the abdominal region of the flies soon after feeding. In the control group, flies were fed exclusively with 5% yeast and food dye. For each set, 25 flies in triplicates were included. 10 days of observations were carried out to check the mortality rate and the survival curve was plotted to compare the statistical significance between the control and treatment groups.

**Climbing/negative geotaxis assay for *Drosophila melanogaster*:**

Behavioural experiments were carried out on the last day of the survival assay (i.e., 10th day) and at specific time intervals in the morning session to keep the environmental conditions uniform. A climbing assay or negative geotaxis assay was conducted to assess the motor defects in control and IAA-fed flies. Control and IAA-fed flies were transferred to a 25 cm vertical glass tube and the two ends of the tube were closed using parafilm with minute holes for air exchange. The flies were tapped 2‒3 times to ensure that all the flies were at the bottom of the tube. The flies were then allowed to climb for 20 cm and the number of flies that crossed the mark in 1 minute was counted. This procedure was repeated three times and a percentage value was used for plotting the final graph.

**Chemotaxis and phototaxis assays for *Drosophila melanogaster*:** An olfactory conditioning assay was conducted to test the odour preferences and phototaxis of control and IAA-treated *D. melanogaster* using a T-maze. Control and IAA-treated flies (n=10 each per replicate, total 3 replicates) were used in the assay. Two odours, 1-octanol and ethanol were introduced simultaneously through the two arms of T-maze. The flies were allowed to choose between 1-octanol and ethanol as well as light and darkness. The number of flies moving towards respective stimuli was calculated.

**Fly head protein extraction and digestion**

IAA-fed and –unfed male and female *D. melanogaster* head homogenates (500 µL each) were subjected to protein extraction by acetone precipitation (at -20 °C overnight). Next, acetone was removed and the protein pellet was dried and resuspended in 50 mM triethylammonium bicarbonate buffer (TEABC). Protein was quantified using a bicinchoninic acid (BCA) assay (Pierce, Waltham, MA). Equal amounts of protein (200 µg) were reduced using 100 mM DTT (incubated at 60 °C for 30 minutes) followed by alkylation using 100 mM IAA (incubated at room temperature for 1 h). Proteins were digested with modified sequencing-grade trypsin (Promega, Madison, WI) with 1:20 substrate/enzyme ratio at 37 °C overnight. Peptides were vacuum-dried and resuspended in 0.1% formic acid and desalted by the C18 stage-tip method. Peptide estimation was carried out using a Pierce quantitative peptide assay kit (Pierce, Waltham, MA).

**Reverse phase fractionation**

To build an in-house spectral library, 20 µg of peptides from each sample were pooled, dried and reconstituted in 0.1% formic acid (FA). Peptide fractionation was carried out using an in-house prepared stage tip column-based protocol. The C18 material was stacked into 200 μl tip and activated by adding 100 μl of 100% acetonitrile (ACN) followed by equilibration with 0.1% FA. The reconstituted samples were loaded onto the activated stage tips to bind peptides to C18 material. The flow-through was passed thrice, followed by washing with 0.1% FA. Peptides were eluted into 24 fractions using a 2‒80% gradient of ACN and 0.1% FA. The 24 fractions were depressively concatenated into 6 fractions.

## **LC-MS/MS analysis by Data-Independent Acquisition (DIA) mode**

An Orbitrap Fusion Tribrid mass spectrometer (Thermo Fischer Scientific, Bremen, Germany) connected to the Easy- nLC-1200 nanoflow liquid chromatography system (Thermo Scientific) was used for data acquisition. All samples were loaded onto a trap column (Acclaim PepMap, 2 cm X 0.75 µ ID, 2 µm) and using an analytical column (Acclaim PepMap, 15 cm X 0.75 µ ID, 2 µm) peptides were separated and eluted by passing mobile phase B (0.1% FA in 80% ACN) in gradient mode for 120 min at 300 nL/min flow rate, and at column temperature of 45 °C.

**Data-Dependent Acquisition (DDA) to generate spectral library**

Reverse phase fractions (n=6) were subjected to an MS survey scan at a mass scan range of 400−1600 m/z (120000 mass resolution at 200 m/z) in a data-dependent mode using an Orbitrap mass analyser with a maximum injection time of 50 ms. Peptides with a charge state of 2−7 were considered for analysis with a dynamic exclusion rate of 45 s. For MS/MS analysis, data was acquired at top speed mode with a 3 s cycle and subjected to higher collision energy dissociation with 35% normalized collision energy. MS/MS scans were carried out at a range of 100−2000 m/z using the Orbitrap mass analyzer at a resolution of 30000 at 100 m/z. The maximum injection time was 200 ms.

### **Wide-window DIA analysis**

The digested samples were resuspended with 0.1% formic acid. An equal volume (~2 µg/µL) of the sample and iRT peptide mixture (~250 fmol) was spiked and loaded onto a 96-well plate. Samples were analyzed through data-independent acquisition (DIA) mode. The precursor molecules in the 380‒1080 m/z range were filtered in quadrupole with a 25 Da isolation window (0.5 m/z margin) and were fragmented at 30% HCD. There were 30 isolation windows in each cycle of DIA with a maximum injection time of 60 ms and an automatic gain control (AGC) target value of 1e6. The fragment ions within the m/z 145‒2000 range were detected in Orbitrap at resolution 30000 at 100 m/z. Between each 20 MS/MS scans, one Full MS scan for the range of 350‒1500 m/z was performed in Orbitrap at 120K resolution, with a maximum injection time of 50 ms and an automatic gain control (AGC) target value of 1e6.

### **Gas-phase fractionation (GPF) by narrow-window DIA**

### A small amount of peptides (5 μg from each sample) were pooled and used for gas-phase fractionation (GPF) using a narrow-window DIA method. For the precursors with m/z ranges of 380‒1080, the pooled sample was fractionated into 7 groups (380‒482, 480‒582, 580‒682, 680‒782, 780‒882, 880‒982, 980‒1080). Each fraction was acquired separately with 4 m/z X 52 isolation windows (generated with Skyline version 21.1.0.146) and the overlap deconvolution method. GPF was performed in between fixed wide window DIA runs. The remaining LC-MS/MS parameters were the same as those used in the wide window DIA method.

**Building an *in-silico* predicted spectral library of *Drosophila melanogaster* head proteins**

FASTA sequences of *D. Melanogaster* proteins were downloaded from the UniProt database (July 2023, 42757 entries). Using the DIA-NN software we generated an *in-silico* spectral library from this FASTA file. Here, we utilized the deep learning-based spectra, and retention time prediction feature of the software. The FASTA sequences were used for generating an *in-silico* spectral library (precursor FDR=1%) by considering trypsin as the enzyme and up to 1 missed cleavage was allowed. All other parameters were set to default.

### **Building in-house spectral library from DDA data**

The data acquired through DDA mode were used to build an in-house spectral library. Using Proteome Discoverer 2.2 (Thermo Fisher Scientific, Bremen, Germany), DDA data was searched against the UniProt protein database of *D. Melanogaster* and known contaminants (116 entries) using SequestHT and MASCOT algorithms. Oxidation of methionine and protein N-terminal acetylation were set as dynamic modifications and carbamidomethylation of cysteine was set as static modification. Minimum peptide length was set to six amino acids and up to two missed cleavages were allowed. Proteins were identified at 1% False Discovery Rate (PSM, peptide and protein level) with a mass tolerance of 20 ppm at MS level and 0.02 Da at MS/MS level. Further, Skyline (version 21.1.0.146) was used to convert the “.pdresults” file into a spectral library.

**Chromatogram library from GPF**

The GPF data was searched against the *D. Melanogaster* predicted spectral library previously generated using DIA-NN software. The parameters used were the same as those used for predicted library generation. This search generated a chromatogram library from the GPF data. Further, all 7 GPF files were merged into a single file using ProteoWizard (18) which was used to generate a calibrated iRT calculator in Skyline. This single file was searched against the previously generated chromatogram library. Finally, the peptide precursors were reintegrated with a decoy-trained mProphet model in which the “Retention time difference” feature score was ignored during model training. After reintegrating the peptide precursors, 14 Pierce iRT peptides (iRT-C18) were chosen and calibrated against the entire chromatogram library of reintegrated peptide precursors. Such a chromatogram model/library generated by integrating the iRT calculations was used to identify proteins in the DIA data.

**DIA data analysis: spectral library search**

The DIA data was searched against two spectral libraries: 1) predicted/*in-silico* spectral library generated through DIA-NN software, and 2) ESL developed using DDA and GPF data. First, the DIA data was searched against the PSL using DIA-NN software. Proteins were identified and quantified at 1% FDR, considering trypsin as the enzyme and up to 1 allowed missed cleavage. All other parameters were set to default. A detailed statistical report generated by DIA-NN software during the PSL search is shown in Fig. S3.

Next, the DIA data was searched against the ESL as follows. Using Skyline (version 21.1.0.146), all DIA raw files were searched against the in-house spectral library and chromatogram library built from DDA and GPF data, respectively. The calibrated iRT model was incorporated into the Skyline document. Decoy peptides were created by inverting the sequence and added to the document. Peptide settings were as follows: the digestion parameters- enzyme: trypsin [KR|P] and max missed cleavages: 2; background proteome: *D. melanogaster*; enforce peptide uniqueness by: none options were selected. Peptides with 7‒50 amino acids were filtered by auto-selecting all matched transitions. Peptides that matched both library and filter were picked and ranked by picked intensity. Carbamidomethyl at cysteine (C) was selected as a fixed modification, and acetylated N-terminal and oxidation at methionine (M) were selected as variable modifications. Transition settings were as follows: Precursor charges from 2‒6 were considered with ion charges:1 and 2, and ion types y, b, and p. Product ions from “ion 3” to “last ion” were selected. All matching transitions were auto-selected and “use DIA precursor window for exclusion” options were selected under the filter tab. In the library tab, the ion match tolerance parameter was set to 0.02 m/z and the option “if a library spectrum is available, pick its most intense ions” (Product ion range was given as 3‒6 ions) from filtered product ions were selected. Min m/z of 145 m/z, and a Max m/z of 2000 m/z from 3‒100 min were set under the Instrument tab. For MS1 filtering, an Orbitrap mass analyzer with a resolving power of 1,20,000 at 200 m/z was employed with 3 isotope peaks. For MS/MS filtering the acquisition method was set as DIA with isolation schema for 380‒1080 m/z range with 20m/z window width and 0.5 m/z margin. Centroided with a mass accuracy of 20 ppm was set for the product mass analyzer. For retention time filtering, we used only scans within 5 minutes of the predicted RT being set. After importing all the files into the Skyline document, peptide precursors which are having less than 6 transitions (at least three precursor and product ions each) were discarded. The remaining peptide precursors were reintegrated using a decoy-trained mProphet model. Finally, peptide precursors that had a q-value of less than 0.01 were considered true identifications. Fig. S4 and S5 explain the retention time distribution and target-decoy analysis model along with the mass error distribution of DIA data as compared to ESL, respectively.

**References**

1. Seemann T (2014) Prokka: rapid prokaryotic genome annotation. *Bioinformatics* 30:2068‒2069.
2. Saier MH, *et al.* (2016) The Transporter Classification Database (TCDB): recent advances. *Nucleic Acids Res* 44:D372‒379.
3. Mao C, *et al.* (2015) Curation, integration and visualization of bacterial virulence factors in PATRIC. Bioinformatics 31:252‒258.
4. Chen L, Zheng D, Liu B, Yang J, Jin Q (2016) VFDB 2016: hierarchical and refined dataset for big data analysis--10 years on. *Nucleic Acids Res* 44:D694‒697.
5. Zhu F, *et al.* (2012) Therapeutic target database update 2012: a resource for facilitating target-oriented drug discovery. *Nucleic Acids Res* 40:D1128‒1136.
6. Law V, *et al.* (2014) DrugBank 4.0: shedding new light on drug metabolism. *Nucleic Acids Res* 42:D1091‒1097.
7. McArthur AG, *et al.* (2013) The comprehensive antibiotic resistance database. *Antimicrob Agents Chemother* 57:3348‒3357.
8. Davis JJ, et al. (2016) PATtyFams: Protein Families for the Microbial Genomes in the PATRIC Database. *Front Microbiol* *7*:118.
9. Long SW, *et al.* (2017) Population Genomic Analysis of 1,777 Extended-Spectrum Beta-Lactamase-Producing *Klebsiella pneumoniae* Isolates, Houston, Texas: Unexpected Abundance of Clonal Group 307 *mBio* 8.
10. Tatusov RL, Galperin MY, Natale DA, Koonin EV (2000) The COG database: a tool for genome-scale analysis of protein functions and evolution. *Nucleic Acids Res* 28.
11. Ashburner M, *et al.* (2000) Gene ontology: tool for the unification of biology. The Gene Ontology Consortium. *Nat Genet* 25:25‒29.
12. Ogata H, Goto S, Fujibuchi W, Kanehisa M (1998) Computation with the KEGG pathway database. *Biosystems* 47:119‒128.
13. Thompson JD, Gibson TJ, Plewniak F, Jeanmougin F, Higgins DG (1997) The CLUSTAL_X windows interface: flexible strategies for multiple sequence alignment aided by quality analysis tools. *Nucleic Acids Res* 25:4876‒4882.
14. Saitou N, Nei M (1987) The neighbor-joining method: a new method for reconstructing phylogenetic trees. *Mol Biol Evol* 4:406‒425.
15. Tamura K, *et al.* (2011) MEGA5: molecular evolutionary genetics analysis using maximum likelihood, evolutionary distance, and maximum parsimony methods. *Mol Biol Evol* 28: 2731‒2739.
16. Kimura M (1980) A simple method for estimating evolutionary rates of base substitutions through comparative studies of nucleotide sequences. *J Mol Evol* 16:111‒120.
17. Felsenstein J (1985) Confidence Limits on Phylogenies: An Approach Using the Bootstrap. *Evolution* 39:783‒791.
18. Chambers MC, *et al.* (2012) A cross-platform toolkit for mass spectrometry and proteomics. *Nat Biotechnol* 30:918–920.
