# Supplementary material for: Comprehensive insights into the impact of bacterial indole-3-acetic acid on sensory preferences in Drosophila melanogaster
Source: Sci Rep. 2024 Apr 9;14:8311. doi: 10.1038/s41598-024-58829-7 (PMC11003987; doi:10.1038/s41598-024-58829-7)
Supplement: Supplementary file 2 — Supplementary Information 2. [file 41598_2024_58829_MOESM2_ESM.pdf]

Supplementary Information

Table S1. Genes encoding proteins/enzymes involved in alcohol metabolism, and the production of tryptophan and hormones in NEEL19

Prokka_ID	Start	End	Strand	EC No.	Gene	Product
				**Alcohol utilization**		
EBOPCKPK_02169	2388661	2389824	+	1.1.1.192	*adh1*	Long-chain-alcohol dehydrogenase 1
EBOPCKPK_02535	2829657	2830820	-	1.1.1.1	*adhB*	Alcohol dehydrogenase 2
				PQQ type (Cu2+ containing)		
EBOPCKPK_02538	2833204	2834991	-	1.1.2.8	*qedA_1*	Quinoprotein alcohol dehydrogenase (cytochrome c)
EBOPCKPK_02547	2843721	2844182	-	1.1.9.1	*qbdA*	Quinohemoprotein alcohol dehydrogenase ADH IIB
EBOPCKPK_02548	2844437	2846332	+	1.1.2.8	*qedA_2*	Quinoprotein alcohol dehydrogenase (cytochrome c)
				Medium chain alcohol dehydrogenases		
EBOPCKPK_00550	583065	584153	-	1.-.-.-	*ydjJ*	putative zinc-type alcohol dehydrogenase-like protein YdjJ
EBOPCKPK_01902	2102360	2103370	-	1.1.1.1	*adhP*	Alcohol dehydrogenase%2C propanol-preferring
EBOPCKPK_01964	2164672	2165724	+	1.1.1.2	*adhc1*	NADP-dependent alcohol dehydrogenase C 1
EBOPCKPK_02425	2713655	2714857	+			putative zinc-binding alcohol dehydrogenase
EBOPCKPK_02478	2769471	2770511	-			Alcohol dehydrogenase
EBOPCKPK_02661	2965203	2966210	+			Zinc-type alcohol dehydrogenase-like protein
EBOPCKPK_02665	2971326	2972549	+			putative zinc-binding alcohol dehydrogenase
EBOPCKPK_04165	4626759	4627769	-	1.1.1.1	*adhT*	Alcohol dehydrogenase
EBOPCKPK_00014	15162	16808	+	1.1.99.-	*alkJ_1*	Alcohol dehydrogenase [acceptor]
EBOPCKPK_00708	769662	771257	+	1.1.99.-	*alkJ_2*	Alcohol dehydrogenase [acceptor]
EBOPCKPK_03146	3509821	3511410	-	1.1.99.-	*alkJ_3*	Alcohol dehydrogenase [acceptor]
				**Tryptophan biosynthesis**		
EBOPCKPK_00040	41566	42375	-	4.2.1.20	*trpA*	Tryptophan synthase α chain
EBOPCKPK_00041	42372	43589	-	4.2.1.20	*trpB*	Tryptophan synthase β chain
EBOPCKPK_00410	461994	462827	+	4.1.1.48	*trpC*	Indole-3-glycerol phosphate synthase
EBOPCKPK_00409	460948	461997	+	2.4.2.18	*trpD*	Anthranilate phosphoribosyltransferase
EBOPCKPK_00406	456497	457978	+	4.1.3.27	*trpE*	Anthranilate synthase component 1
EBOPCKPK_03359	3743991	3744611	-	5.3.1.24	*trpF*	N-(5'-phosphoribosyl)anthranilate isomerase
EBOPCKPK_00408	460345	460938	+	4.1.3.27	*trpG*	Anthranilate synthase component 2
				**Formation of indole-3-acetic acid from tryptophan (indole-3-acetamide pathway)**		
EBOPCKPK_00353	400572	402254	-	1.13.12.3	*iaaM*	Tryptophan 2-monooxygenase
EBOPCKPK_02437	2723242	2724600	-	3.5.99.5	*cnbH*	2-amino-5-chloromuconic acid deaminase
				**Formation of Dopamine**		

EBOPCKPK_01539	1663274	1664062	-	1.14.16.1	*phhA (AAAH)*	Phenylalanine-4-hydroxylase
EBOPCKPK_02721	3028513	3029925	-	4.1.1.86	*ddc (AADC)*	Aromatic-L-amino-acid decarboxylase

Table S2. Details of *Pseudomonas* strains used in the comparative genomic analysis

				Trp biosyntehsis							IAA biosyn.		Dopamine biosyn.		Alcohol deg.	
**SL. No**	**Genome**	**Taxon name***	**Source and country**	**trpA**	**trpB**	**trpC**	**trpD**	**trpE**	**trpF**	**trpG**	**iaaM**	**iaaH**	**AADC**	**AAAH**	**adh1**	**adhB**
1	GCF_000026105.1	*P. entomophila* L48^{T}	female fruit fly *D. melanogaster,* Guadeloupe Island	1	1	1	1	1	1	1	1	0	1	1	1	1
2	GCF_000621225.1	*P. mosselii* DSM 17497^{T}	clinical specimen, NA	1	1	1	1	1	1	1	1	0	1	1	1	1
3	GCF_000730665.1	*P. plecoglossicida* NBRC 103162^{T}	bacterial haemorrhagic ascites of ayu, Japan	1	1	1	1	1	1	1	1	0	1	1	1	1
4	GCF_000425785.1	*P. taiwanensis* DSM 21245^{T}	soil, Taiwan	1	1	1	1	1	1	1	1	0	1	1	1	1
5	GCF_000730645.1	*P. parafulva* NBRC 16636^{T}	Japanese rice paddy, Japan	1	1	1	1	1	1	1	1	0	1	1	1	1
6	GCF_900277125.1	*P. inefficax* JV551A3^{T}	soil, France	1	1	1	1	1	1	1	1	0	1	1	1	1
7	GCF_000730605.1	*P. monteilii* NBRC 103158^{T}	bronchial aspirate, *Homo sapiens* , France	1	1	1	1	1	1	1	1	0	1	1	1	1
8	GCF_009932335.1	*P. asiatica* RYU5^{T}	hospitalized patient's stool sample, Japan	1	1	1	1	1	1	1	1	0	1	1	1	1
	Pseudomonas juntendi															
9	GCF_000190455.2	*Pseudomonas* sp. TJI-51	mango orchid, NA	1	1	1	1	1	1	1	1	1	1	1	1	1
10	GCF_000710785.1	*Pseudomonas putida* T2-2	tongue, NA	1	1	1	1	1	1	1	1	1	1	1	1	1
11	GCF_001753875.1	*Pseudomonas* sp. GTC 16481	*Homo sapien,* Japan	1	1	1	1	1	1	1	1	1	1	1	1	1
12	GCF_001320385.1	*Pseudomonas* sp. NBRC 111128	*Homo sapien,* Japan	1	1	1	1	1	1	1	1	1	1	1	1	1
13	GCF_004350515.1	*Pseudomonas aeruginosa* 147	hospital (host:Homo sapiens), Italy	1	1	1	1	1	1	1	1	1	1	1	1	1
14	GCF_002927165.1	*Pseudomonas aeruginosa* T36994	urine, *Homo sapien,* USA	1	1	1	1	1	1	1	1	1	1	1	1	1
15	GCF_003935475.1	*Pseudomonas* sp. p99-361	nursing call button in hospital ICU, Pakistan	1	1	1	1	1	1	1	1	1	1	1	1	1
16	GCF_001320435.1	*Pseudomonas* sp. NBRC 111129	*Homo sapien,* Japan	1	1	1	1	1	1	1	1	1	1	1	1	1
17	GCF_001753895.1	*Pseudomonas* sp. NBRC 111120	*Homo sapien,* Japan	1	1	1	1	1	1	1	1	1	1	1	1	1
18	GCF_001645635.1	*Pseudomonas putida* KB9	apoplast, *Arabidopsis thalian,* South Korea	1	1	1	1	1	1	1	1	1	1	1	1	1
19	GCF_001321005.1	*Pseudomonas* sp. NBRC 111144	*Homo sapien,* Japan	1	1	1	1	1	1	1	1	0	1	1	1	1
20	GCF_001753935.1	*Pseudomonas* sp. NBRC 111126	*Homo sapiens,* Japan	1	1	1	1	1	1	1	1	1	1	1	1	1
21	GCF_007633355.1	*Pseudomonas* sp. BJP69	sputum, *Homo sapien,* China	1	1	1	1	1	1	1	1	0	1	1	1	1
22	GCF_008605605.1	*Pseudomonas putida* JYR-1	NA	1	1	1	1	1	1	1	1	1	1	1	1	1
23	GCF_900455605.1	*Pseudomonas putida* NCTC912	NA	1	1	1	1	1	1	1	1	0	1	1	1	1
24	GCF_009668315.1	*Pseudomonas* sp. CAH-1	NA, USA	1	1	1	1	1	1	1	1	0	1	1	1	1
25	GCF_003936655.1	*Pseudomonas* sp. p99-361 p99	nursing call button in hospital ICU, Pakistan	1	1	1	1	1	1	1	1	0	1	1	1	1
26	GCF_001320345.1	*Pseudomonas* sp. NBRC 111127	*Homo sapien,* Japan	1	1	1	1	1	1	1	1	1	1	1	1	1
**27**	**GCF_009932375.1**	***Pseudomonas juntendi* BML3^{T}**	**sputum, *Homo sapien,* Japan**	**1**	**1**	**1**	**1**	**1**	**1**	**1**	**1**	**1**	**1**	**1**	**1**	**1**
28	GCF_003205245.1	*Pseudomonas* sp. MB-090624	NA, USA	1	1	1	1	1	1	1	1	1	1	1	1	1
	*, Based on EzBiocloud; 1, present; 0, absent; NA, not available															

Table S3. Proteins identified by searching DIA data against experimental spectral library developed using DDA data and iRT integrated GPF data. Proteins that are identified in at least 2 replicates in any one of the four conditions (CF, CM, IAAF, IAAM) were chosen as true identifications. The abundances shown here are raw abundances.

Protein Accession	C_F_R1	C_F_R3	C_F_R3	C_M_R1	C_M_R2	C_M_R3	IAA_F_R1	IAA_F_R2	IAA_F_R3	IAA_M_R1	IAA_M_R2	IAA_M_R3
A0A023GQA5	524500	499920	267500	267500	543660	221440	350250	380420	390660	211170	188500	19376
A0A0B4K7G4	145720	121120	17764	17764	24361	17352	88588	105270	99334	0	145360	38439
A0A0B4KEW6	857650	830150	0	0	256380	350400	138210	154080	143270	48907	77240	14281
A0A0B4KFT0	1048200	1101600	461250	461250	490240	418020	365340	411620	428310	314640	306900	55810
A0A0B4KFZ9	1146700	1098600	349830	349830	378420	339610	84730	105210	96645	69308	41752	45420
A0A0B4KG37	226240	268140	160130	160130	170770	145880	243500	276700	251210	168280	149090	9285
A0A0B4KGT7	82341	84677	189660	189660	242990	201430	148020	151020	123150	77074	61362	0
A0A0B4KH25	304830	1212200	209790	209790	131440	112780	53005	933420	797610	870330	838000	27413
A0A0B4KHJ9	369110	285640	135400	135400	130240	118510	21710	22945	20658	7273	9417	0
A0A0B4LEY6	572270	345450	133480	133480	135150	113880	157150	153510	132880	74241	58081	17945
A0A0B4LFL3	4066600	4143400	1189000	1189000	1130600	1011100	733540	1014100	890450	677620	609030	272510
A0A0B4LFM0	823260	751150	757410	757410	493110	395180	93021	297400	314770	186060	135350	19690
A0A0B4LGS4	1250800	1124500	392350	392350	461260	357720	155960	210270	153110	93699	70968	12019
A0A0B4LGZ7	1522400	1494400	698000	698000	715540	640080	767060	0	0	474030	450280	66138
A0A0B4LHE7	806020	981980	339130	339130	320290	296190	180840	269140	222250	170440	155420	0
A0A0B4LHL7	946150	868550	0	0	0	0	0	0	0	345220	282320	0
A0A1B2AIV9	1724900	1728300	150040	150040	145260	123120	145950	159410	151090	43972	45330	0
A0A1B2AIW6	2314300	2674000	756530	756530	704460	557480	428230	213380	369400	437100	339890	123320
A0A1B2AJ59	139380	110480	34819	34819	22523	31745	16191	28704	16794	31560	15824	0
A0A1B2AJI5	1181600	1048300	586270	586270	620550	547610	113050	167380	151910	980220	909070	130570
A0A1B2AJW2	691290	623840	367040	367040	309070	211120	1116600	1455000	1428200	0	0	289110
A0A1B2AK36	599570	420380	194540	194540	745490	609380	198200	242010	217480	166540	180840	17606
A0A1B2AKN7	826720	709470	448900	448900	462330	454050	212940	302710	319910	442990	415070	55951
A0A1B2AKQ4	0	1965000	881150	881150	889070	829270	740150	797090	772420	566680	523300	82149
A0A1B2AL87	359400	379400	156970	156970	2886600	384950	1975200	3160200	147110	3044000	3422600	1018000
A0A1B3Q3M8	123520	115320	66702	66702	63480	54800	43945	48356	38539	37521	33968	0
A0A1B3Q3N5	882500	901640	57662	57662	62699	56616	136950	193250	188970	122910	120970	17952
A1Z8D0	139340	152700	0	0	0	491950	60466	67176	62890	22836	30136	0
A1Z8H6	286140	184030	209240	209240	141140	174210	509320	99100	125700	93891	178220	138510
A1ZA73	1130000	1273200	793740	793740	774240	684480	517260	1026100	918770	451880	397590	51252
A1ZB23	212950	214480	122580	122580	117540	102330	85457	106430	112670	85172	75332	3517
A4V1N8	959250	937120	1521500	1521500	0	265940	0	0	0	0	0	0
A4V383	1922600	1820200	502410	502410	661860	581960	606650	726940	776810	321080	250270	31199
A4V4W0	485180	515360	364130	364130	416210	349500	417980	491480	403840	339640	194290	780270

A8DRW0	244740	225130	151480	151480	189720	175140	0	538050	493140	315910	291850	36098
A8E774	436420	406380	162240	162240	194280	182210	229220	291380	274780	194550	163590	29752
B5RIU6	716690	695660	558850	558850	576460	473940	532540	630090	665430	582000	530690	106530
B7Z107	234450	232760	49644	49644	35292	40615	56099	62226	47926	42644	40547	5845
C0HK92	1522400	1494400	698000	698000	715540	640080	767060	0	0	474030	450280	66138
C0HK95	359400	379400	156970	156970	2886600	384950	1975200	3160200	147110	3044000	3422600	1018000
C0HKA0	1374400	1527400	198260	198260	223820	191210	130170	155790	154850	35600	51438	15345
C0HKA1	1374400	1527400	198260	198260	223820	191210	130170	155790	154850	35600	51438	15345
C0HLZ9	175130	150210	179910	179910	195920	146060	250750	0	196890	151230	140600	13945
C0HM00	175130	150210	179910	179910	195920	146060	250750	0	196890	151230	140600	13945
C4NYP8	317470	340050	111870	111870	94690	88317	55794	58410	56887	560490	24375	66386
C7LA94	1329400	1218000	315760	315760	321560	299780	185840	52276	75541	28011	30785	0
D2NUG3	1951000	1644800	1133800	1133800	1201500	1122600	691470	1081700	1034700	965940	839330	111000
D5A7M1	5557800	4921900	2261700	2261700	2268100	2067400	2324000	2855700	2877900	3254700	3190500	145840
E1JGR4	708150	1216700	1274800	1274800	460380	993990	316010	362040	488890	513660	567100	141880
E1JHQ1	588830	554860	308410	308410	360030	296330	164120	234720	214790	176390	181280	24998
E1JIJ5	545080	522900	141670	141670	170910	122900	77152	94188	0	225990	51859	0
E2QD63	317470	340050	111870	111870	94690	88317	55794	58410	56887	560490	24375	66386
F3YDH0	733480	699340	148010	148010	159630	125410	203870	291680	220240	0	0	76830
H0RN81	2059200	2028600	768230	768230	722470	668970	0	390880	356800	0	254350	0
I0DHK3	1038100	930070	329130	329130	365200	294000	248860	336410	263420	182940	177200	11830
M9MSK4	350510	159280	241300	241300	140790	196560	114550	140990	245190	190020	172330	26625
M9NCS8	662140	704340	213230	213230	224150	0	383410	224070	232110	105660	99011	0
M9NE68	1113500	978950	483970	483970	559600	520920	195260	292080	278630	365010	326910	51270
M9PBA3	1081900	1033200	339290	339290	368090	341270	605540	666170	676760	241690	243560	34018
M9PCE0	1222800	1124100	396710	396710	383830	368940	183790	254360	223000	152300	147060	12829
M9PCU0	740590	754980	1016700	1016700	847760	743660	358920	386190	384100	265050	1426200	156220
M9PD14	1526200	1368400	464510	464510	626990	523690	337020	461770	752620	1006400	851690	281250
M9PD75	169970	148100	51546	51546	1280200	316940	47298	1422500	67683	495530	478050	68490
M9PEG1	361260	156850	78238	78238	67869	57001	112730	135980	142650	73039	74333	125960
M9PFZ6	234760	197530	119570	119570	122790	97575	25684	0	21573	25683	46001	4304
M9PG76	177810	155600	37555	37555	43114	38595	312550	39071	41944	31204	28948	59795
O02195	246430	233080	90758	90758	74374	78686	29340	59127	49349	18981	11708	34577
O02649	1329400	1218000	315760	315760	321560	299780	185840	52276	75541	28011	30785	0
O16043	1359900	1068100	832310	832310	853220	746650	532370	838200	839790	717620	654360	59124
O17444	1951000	1644800	1133800	1133800	1201500	1122600	691470	1081700	1034700	965940	839330	111000
O17452	593540	519680	334510	334510	335100	278910	282170	412090	375310	278450	256920	19456
O96827	4066600	4143400	1189000	1189000	1130600	1011100	733540	1014100	890450	677620	609030	272510
O97418	3330200	0	1519300	1519300	1412800	1291800	1554300	1770700	1553500	1295500	1209800	247430
P02283	2616100	2458000	1161600	1161600	1298200	1139600	1523800	1891100	1624200	600980	611930	78063

P02516	1113500	978950	483970	483970	559600	520920	195260	292080	278630	365010	326910	51270
P02574	234760	197530	119570	119570	122790	97575	25684	0	21573	25683	46001	4304
P05205	463540	420080	274730	274730	298790	257670	271940	282810	267470	156740	149250	199650
P08985	304830	1212200	209790	209790	131440	112780	53005	933420	797610	870330	838000	27413
P09491-2	369110	285640	135400	135400	130240	118510	21710	22945	20658	7273	9417	0
P10676	772590	699200	360260	360260	359250	316080	276960	322090	321000	205830	205160	21813
P10676-2	740590	754980	1016700	1016700	847760	743660	358920	386190	384100	265050	1426200	156220
P11046	8016	6285	58074	58074	2049	580	131470	131380	1936	0	0	0
P12881	1038100	930070	329130	329130	365200	294000	248860	336410	263420	182940	177200	11830
P13060	366490	1355900	227530	227530	1111500	953050	168870	206210	180550	831510	706410	0
P13469	145720	121120	17764	17764	24361	17352	88588	105270	99334	0	145360	38439
P14318	1070000	1193200	376970	376970	406340	339150	188870	274220	682710	360520	0	0
P14484	609520	764870	368330	368330	583100	302420	164790	351350	191520	200280	273050	25385
P15215	173650	167830	12005	12005	53520	48684	44828	54386	43572	38493	26805	0
P18432	507080	471390	301520	301520	311220	267010	193250	221700	190190	168980	160600	9852
P19889	177810	155600	37555	37555	43114	38595	312550	39071	41944	31204	28948	59795
P20477	588830	554860	308410	308410	360030	296330	164120	234720	214790	176390	181280	24998
P21187	351090	344350	32818	32818	40778	34635	22103	39689	33224	109070	99382	2382
P23696	419150	321690	272830	272830	190530	213140	49484	169440	126760	338330	329570	0
P23779	1438300	0	913870	913870	750420	409500	218100	131300	55627	78102	82334	182130
P24156	524500	499920	267500	267500	543660	221440	350250	380420	390660	211170	188500	19376
P25007	436420	406380	162240	162240	194280	182210	229220	291380	274780	194550	163590	29752
P29829	457920	418770	246190	246190	231070	203620	117840	229080	223710	144140	138610	13804
P29844	733480	699340	148010	148010	159630	125410	203870	291680	220240	0	0	76830
P31409	545080	522900	141670	141670	170910	122900	77152	94188	0	225990	51859	0
P33438	662140	704340	213230	213230	224150	0	383410	224070	232110	105660	99011	0
P35415	959250	937120	1521500	1521500	0	265940	0	0	0	0	0	0
P35554	1526200	1368400	464510	464510	626990	523690	337020	461770	752620	1006400	851690	281250
P41044	823260	751150	757410	757410	493110	395180	93021	297400	314770	186060	135350	19690
P41964	691290	623840	367040	367040	309070	211120	1116600	1455000	1428200	0	0	289110
P42281	2314300	2674000	756530	756530	704460	557480	428230	213380	369400	437100	339890	123320
P42325	601290	1010000	316740	316740	528260	358610	311680	553630	602570	383960	0	139640
P45594	0	1965000	881150	881150	889070	829270	740150	797090	772420	566680	523300	82149
P47949	1556700	1454500	250660	250660	279360	163500	5175100	6583700	5244700	826970	0	0
P48375	2059200	2028600	768230	768230	722470	668970	0	390880	356800	0	254350	0
P48588	882500	901640	57662	57662	62699	56616	136950	193250	188970	122910	120970	17952
P49630	253430	248890	53075	53075	432610	317070	34104	273220	199390	204360	129250	46958
P54399	1696600	177710	94560	94560	82936	103460	0	0	0	0	0	0
P54611	1250800	1124500	392350	392350	461260	357720	155960	210270	153110	93699	70968	12019
P61849	82341	84677	189660	189660	242990	201430	148020	151020	123150	77074	61362	0

P61851	1747400	1683200	905040	905040	919850	818510	1280400	1395800	1408600	1395900	1560400	234260
P80455	1264500	1402200	369720	369720	333810	419060	460620	488560	461450	238640	253690	18589
P84040	1146700	1098600	349830	349830	378420	339610	84730	105210	96645	69308	41752	45420
P91929	1922600	1820200	502410	502410	661860	581960	606650	726940	776810	321080	250270	31199
P92181	2482900	2165600	1191700	1191700	1215700	1163400	1088400	1360500	1495600	930470	860690	133840
Q01604	1222800	1124100	396710	396710	383830	368940	183790	254360	223000	152300	147060	12829
Q03427	186700	172990	46741	46741	48377	41078	28681	27049	26859	16061	12159	22268
Q09103-3	637540	537890	313760	313760	290640	267210	73333	165850	153140	57108	70108	12859
Q0E8V7	508260	362460	250710	250710	140380	144390	67559	75879	97070	168830	181510	50956
Q0E8X8	467950	407210	250910	250910	245480	239780	637160	946140	950130	1151100	1082500	113310
Q0KHX4	415020	403040	86847	86847	82925	149890	89530	290640	163430	119220	22012	0
Q23983	163030	118480	122630	122630	561740	133530	85149	151820	135040	137130	141570	23286
Q24211	485180	515360	364130	364130	416210	349500	417980	491480	403840	339640	194290	780270
Q24251	946150	868550	0	0	0	0	0	0	0	345220	282320	0
Q24269	208590	188120	700720	700720	664160	594720	87549	441790	98690	352950	345660	9703
Q26377	216270	169280	787610	787610	1022300	124250	185990	257520	272880	200240	176660	26461
Q27377	1181600	1048300	586270	586270	620550	547610	113050	167380	151910	980220	909070	130570
Q5U126	1545500	1517000	866210	866210	988280	940530	349650	435870	418480	340030	390330	56886
Q6NLJ9	151940	126600	91958	91958	142100	82181	160670	197640	202440	159520	163130	16492
Q7JR58	567130	603940	171890	171890	178700	175440	181970	116700	312050	125310	136530	22701
Q7JR71	1583200	1498300	495320	495320	548210	495570	276880	406670	375870	384880	389330	48539
Q7JRC0	29680	24074	19311	19311	15895	8541	21950	20370	22028	17607	17520	6826
Q7JV39	588520	591730	215640	215640	264280	226370	290180	368710	324760	248030	242780	29542
Q7JWF1	269470	1225800	840200	840200	644000	189700	164790	267290	222670	654530	165050	0
Q7JZW0	1569400	1606300	715260	715260	732500	646080	729820	935840	853410	603370	486310	121760
Q7K084	9539000	8857200	4289400	4289400	4411200	3886200	3823400	4345900	4127400	3442200	3569300	389020
Q7K1M4	204420	190120	127130	127130	133920	118410	78590	89668	89551	70462	98856	10638
Q7K2D2	1815000	103430	85949	85949	328030	298360	9952900	6740300	0	0	0	0
Q7K3Z3	127710	157470	77645	77645	67958	61125	43130	112050	112840	127690	131470	0
Q7K5J8	171640	156570	123490	123490	74781	100960	143860	198920	160200	104430	107560	18800
Q7K5K3	472090	484430	109250	109250	302170	115540	75303	67065	125800	57279	103750	0
Q7K5M6	25722000	27809000	74046000	74046000	72933000	55942000	0	##########	0	0	##########	50008000
Q7KMQ0	1667100	100090	4557900	4557900	84292	41564	4540600	8486000	128600	112720	7065800	2803300
Q7KV34	69585	0	37293	37293	33325	30765	76689	81738	85647	0	29202	0
Q86DS1	317470	340050	111870	111870	94690	88317	55794	58410	56887	560490	24375	66386
Q8IM93	470740	782790	1099500	1099500	1104700	1062200	430380	494410	469540	361260	771340	22942
Q8IN43	139380	110480	34819	34819	22523	31745	16191	28704	16794	31560	15824	0
Q8IN44	1724900	1728300	150040	150040	145260	123120	145950	159410	151090	43972	45330	0
Q8IQW5	280710	278770	164460	164460	172510	154460	79079	95629	96232	103520	108360	13892
Q8MKJ5	730000	622690	359550	359550	397150	345100	161550	175280	171240	213500	197670	31941

Q8MLN7	0	50193	30306	30306	23222	10010	50170	59442	57869	71067	59816	12447
Q8MSS1	152860	119320	40948	40948	44111	41394	41406	43999	37419	26978	21623	0
Q8MZC1	370180	388000	165450	165450	189350	12786	22277	25383	29332	77608	23279	0
Q8SY67	268010	272100	127160	127160	134570	116330	256580	282440	268600	138790	110330	14683
Q8SYJ2	2238900	1916700	1108400	1108400	1076800	948850	355780	538750	521940	471820	393720	39016
Q8SYQ4	330100	304470	177870	177870	205230	174730	293050	341710	310920	203940	191820	22053
Q8SZA8	608190	594940	214980	214980	214600	182770	332130	407970	344410	356350	320960	89742
Q8T390	716690	695660	558850	558850	576460	473940	532540	630090	665430	582000	530690	106530
Q8T9H4	35279	79251	61669	61669	50282	19598	76450	84659	77557	42414	48872	5169
Q94514	1048200	1101600	461250	461250	490240	418020	365340	411620	428310	314640	306900	55810
Q94518	572270	345450	133480	133480	135150	113880	157150	153510	132880	74241	58081	17945
Q94522	461400	410420	146580	146580	170750	141710	44326	117980	0	60385	46624	6133
Q94920	169970	148100	51546	51546	1280200	316940	47298	1422500	67683	495530	478050	68490
Q95RA9	223710	205450	74125	74125	83226	74672	29806	77548	52109	47235	29210	5167
Q95RB2	711750	621010	333690	333690	329750	303060	261670	174410	337380	0	0	16072
Q960M4	827090	521820	240430	240430	213330	117280	65539	29078	10180	55767	607630	61754
Q9GU68	590930	579500	163370	163370	167060	172370	179750	256330	385770	187710	544720	15044
Q9I7Q5	222900	196540	274380	274380	251620	355800	951680	1127200	930100	844100	664120	156980
Q9V396	635600	711170	543360	543360	278800	192960	373360	311370	319660	291780	355960	139910
Q9V3E7	759130	948730	666450	666450	256610	598400	74382	1073300	1002500	1080300	942870	19678
Q9V3R1	0	90267	40784	40784	0	44571	0	0	0	38935	46091	0
Q9V3W0	330920	333330	78422	78422	95402	64812	33175	40593	35326	63611	50353	3638
Q9V426	482960	363400	178670	178670	192290	174100	69543	93676	88409	215300	267670	0
Q9V521	552810	540150	176590	176590	206600	156150	289760	322800	284980	165030	144170	0
Q9V8Y2	826720	709470	448900	448900	462330	454050	212940	302710	319910	442990	415070	55951
Q9V931	599570	420380	194540	194540	745490	609380	198200	242010	217480	166540	180840	17606
Q9VA32	954130	449860	255930	255930	263440	227760	81911	122680	101250	36409	33304	0
Q9VAC1	255120	280280	82156	82156	81545	78369	103680	134170	135280	89551	99635	10657
Q9VB69	183900	176670	41640	41640	63612	51077	45878	44931	45544	24252	22784	38372
Q9VB81	399750	392490	162790	162790	170540	138590	13201	46418	30919	0	26005	0
Q9VD01	230680	237680	220530	220530	338780	211970	151070	0	94469	0	30734	32121
Q9VD48	259740	261870	287320	287320	288230	260660	215600	305710	262480	226910	208960	35775
Q9VD58	62531	156040	89947	89947	138290	36844	77352	80669	88802	36211	40634	23481
Q9VEB1	637650	561920	269200	269200	214540	157710	308890	449970	420980	0	205870	0
Q9VEJ0	313460	417730	313650	313650	329450	1033000	102270	166040	125030	129040	0	0
Q9VET0	226240	268140	160130	160130	170770	145880	243500	276700	251210	168280	149090	9285
Q9VFF0	2108800	1806400	738530	738530	804040	740250	438330	566080	553160	339490	273580	47255
Q9VFI3	220390	214130	81380	81380	97688	81055	97944	124240	130790	127310	123500	18382
Q9VFV9	225180	163200	2228000	2228000	1893600	1650800	0	0	9338500	0	0	0
Q9VG69	198530	172790	68408	68408	64242	71192	103360	156850	131290	57974	113410	10503

Q9VGA3	87015	92218	25924	25924	29357	22598	11961	9207	11648	0	30270	0
Q9VGK3	220710	332700	82552	82552	66486	68615	39916	44534	40535	183680	40443	65419
Q9VGP7	449650	388260	140300	140300	176580	130160	0	0	0	152840	140670	55866
Q9VGQ1	802080	726520	813820	813820	0	0	886270	1163800	873280	1696400	1556400	210250
Q9VH26	182320	135510	76967	76967	99125	107710	50324	96286	97530	79954	82707	10926
Q9VHC8	165130	168480	80550	80550	78744	72246	87304	115160	98783	94364	73674	12277
Q9VHX4	1146800	989980	266710	266710	332570	265400	46956	68829	100320	63167	178380	1654
Q9VIE8	666510	652190	175550	175550	181910	152450	206030	243770	224480	46173	48161	0
Q9VIH9	460500	433590	194240	194240	196010	178870	262570	290580	265890	325750	307210	52615
Q9VIQ8	502300	398500	259380	259380	281200	210280	212060	249870	233740	75674	67151	17467
Q9VJD1	358990	355060	72488	72488	72305	86469	66421	92205	101040	42545	36618	91854
Q9VJD4	355880	362300	161310	161310	202110	161100	53904	67942	217730	157710	168020	0
Q9VJQ3	404030	366160	138830	138830	132790	93392	55559	69917	75286	56400	51952	5737
Q9VK60	4492500	4554500	1767900	1767900	2000400	1825800	937610	1106700	1031300	805950	577810	319150
Q9VKQ2	91139	43815	36534	36534	35932	30011	150630	177310	109040	79092	56271	3639
Q9VL78	416780	361730	192150	192150	194150	180780	125150	152140	131960	69118	68369	81459
Q9VLP1	967850	644600	332300	332300	377370	314640	461860	613560	687380	594660	492780	38623
Q9VLP2	581330	549330	226960	226960	221260	196170	204570	903130	885450	589570	481160	76901
Q9VLS4	726980	637650	264260	264260	355610	309690	219270	339480	325600	259720	252620	68748
Q9VLU6	123520	115320	66702	66702	63480	54800	43945	48356	38539	37521	33968	0
Q9VMM6	64030	63073	31371	31371	35135	21652	35007	66263	56737	37833	39412	2505
Q9VMM6-2	64634	74932	34414	34414	39093	30172	75901	88238	93175	44408	46525	3166
Q9VNA3	306670	310840	210630	210630	231950	371890	114780	102360	815680	731840	739560	0
Q9VNL0	533790	505670	343200	343200	373150	342130	200310	598190	312230	706200	720290	0
Q9VNW6	268750	257940	99683	99683	111250	216280	254680	411460	330720	0	0	22712
Q9VP57	187270	213760	73323	73323	86962	88292	18502	36811	21205	120200	8917	0
Q9VPC2	398570	450650	174250	174250	163420	186090	206130	244910	250680	185140	171330	178130
Q9VPR2	179230	153330	59313	59313	72381	66643	500130	111420	121130	135280	48419	0
Q9VQT7	653020	536890	3608800	3608800	7087900	111630	281180	313330	317850	354440	296710	55924
Q9VR79	826740	768310	464890	464890	450620	412620	279370	323650	314800	137270	115360	20399
Q9VRL0	293230	246590	105100	105100	136960	135330	255760	264610	279670	173990	156060	16210
Q9VSA9	953240	1002000	609400	609400	647860	554180	155970	226880	204740	234270	191470	29484
Q9VSN3	1487500	2650400	1308200	1308200	1875900	1161000	173350	1126600	1083400	810370	768750	107310
Q9VSP9	102960	106850	283430	283430	420980	25581	24341	41863	120360	0	213540	0
Q9VSY0	612160	416880	363780	363780	417580	222380	687660	886030	822670	328690	340270	37559
Q9VTC3	1512700	1589800	728670	728670	770820	624730	1263400	1714300	1655900	2319400	2072800	344910
Q9VTU2	659220	983020	353770	353770	390420	412450	598410	0	0	396000	0	0
Q9VU35	0	537180	564750	564750	226770	202580	185270	251870	238090	180780	167990	17972
Q9VU75	955380	838010	318990	318990	344270	305050	225050	236550	231140	125300	112830	19701
Q9VUV8	414510	806740	203620	203620	910370	688470	883590	1048900	985630	632130	573510	11464

Q9VV36	7795300	6376700	5104600	5104600	5508000	4781100	1283800	1773000	1761000	1612600	1873900	227100
Q9VV37	429900	439510	545270	545270	617230	554350	295260	374990	355910	219070	218030	24731
Q9VV75	937500	883630	216980	216980	230990	191850	180420	211320	180550	185200	165310	37396
Q9VVC3	200240	104260	63858	63858	79964	66225	29802	37531	62702	0	1724500	0
Q9VVL7	1344400	1279400	547580	547580	521630	628610	366330	515310	363870	568290	0	71441
Q9VWG1	776190	559390	382350	382350	485270	454130	343290	359790	434830	294870	235690	31929
Q9VWI0	356400	339120	178180	178180	169920	140960	169450	197690	159460	95160	81229	168180
Q9VYD7	390460	387930	161310	161310	186590	165820	315490	376810	361820	328510	349460	42930
Q9VZ01	1242400	1360600	1174100	1174100	1199200	1020800	755080	882470	867210	790670	814170	108620
Q9VZ24	479690	452620	202200	202200	186480	190100	274620	339230	310430	241680	235010	38281
Q9W077	2546600	2616300	1726000	1726000	1789300	1614300	550410	707020	646480	493450	521020	90133
Q9W1C9	5557800	4921900	2261700	2261700	2268100	2067400	2324000	2855700	2877900	3254700	3190500	145840
Q9W1F8	133010	136100	83155	83155	84861	75156	159990	183940	178900	111530	103000	9351
Q9W227	1178500	1109700	205620	205620	232860	190790	33514	61977	57234	22054	29547	19873
Q9W266	480540	327430	365540	365540	560240	250470	163520	410610	415120	334850	797180	15735
Q9W2X6	5414400	5507800	2341600	2341600	2990100	1878300	2169700	2260700	1828800	1784800	1553700	373840
Q9W306	0	981420	591180	591180	629570	555840	0	1253500	0	805830	781280	0
Q9W334	8308900	7629300	1820800	1820800	2089900	1982600	789640	837120	796790	166120	149660	47268
Q9W369	275760	213080	1143700	1143700	153930	106130	66373	1179500	131060	1107200	1044400	76523
Q9W3E2	312030	296660	137720	137720	145130	108660	108740	126560	104290	54995	52946	2327
Q9W3N9	367200	326440	646480	646480	644930	584860	238110	288160	198210	123750	2871800	850040
Q9W402	1241400	1209300	194370	194370	69126	209980	93990	18439	16909	45305	0	9760
Q9W4J4	381570	407970	143690	143690	149350	118070	117390	139100	126230	78949	67144	0
Q9XZH6	806020	981980	339130	339130	320290	296190	180840	269140	222250	170440	155420	0
Q9Y114	135600	123190	52405	52405	0	0	40984	151860	55552	58570	51516	63029
Q9Y1A3	1449900	1477500	517500	517500	464000	434380	259660	306820	240170	262530	169710	51070
X2J5E8	416780	361730	192150	192150	194150	180780	125150	152140	131960	69118	68369	81459
X2J8Y6	0	90267	40784	40784	0	44571	0	0	0	38935	46091	0
X2JAF1	152860	119320	40948	40948	44111	41394	41406	43999	37419	26978	21623	0
X2JAW6	173650	167830	12005	12005	53520	48684	44828	54386	43572	38493	26805	0
X2JB87	1449900	1477500	517500	517500	464000	434380	259660	306820	240170	262530	169710	51070
X2JC35	253430	248890	53075	53075	432610	317070	34104	273220	199390	204360	129250	46958
X2JCI6	1556700	1454500	250660	250660	279360	163500	5175100	6583700	5244700	826970	0	0
X2JCX8	1374400	1527400	198260	198260	223820	191210	130170	155790	154850	35600	51438	15345
X2JDI1	419150	321690	272830	272830	190530	213140	49484	169440	126760	338330	329570	0
X2JEM4	8308900	7629300	1820800	1820800	2089900	1982600	789640	837120	796790	166120	149660	47268
X2JEU5	390460	387930	161310	161310	186590	165820	315490	376810	361820	328510	349460	42930
X2JGP4	1696600	177710	94560	94560	82936	103460	0	0	0	0	0	0
A1Z7S3	0	0	0	0	373240	349930	318750	375750	348820	301110	284160	64488
D1Z366	0	0	384960	384960	0	312800	0	680470	619730	308330	261320	126930

M9NGG5	0	0	236680	236680	0	207370	0	0	371250	0	0	0
P29746	0	0	651300	651300	646630	592190	0	1544200	0	0	1240700	180890
P49455	0	0	0	0	1113600	976080	875090	1011600	301000	458960	391120	0
P81160	0	0	384960	384960	0	312800	0	680470	619730	308330	261320	126930
Q9VQT8	0	0	2638900	2638900	2621800	2339900	5134400	6649300	6321900	3882200	3544100	599840
Q9VV46	0	0	1841400	1841400	1799300	1610600	0	0	607330	426790	0	0
Q9W596	0	0	236680	236680	0	207370	0	0	371250	0	0	0
Q9VQF7	0	0	0	0	2796100	0	0	6864400	6356700	6021800	5513900	513290

Table S5. Differentially regulated proteins in IAA-fed flies (IAA) as compared to untreated flies (C).

Protein§	Fold change¶	p-Value†	Protein§	Fold change¶	p-Value†	Protein§	Fold change¶	p-Value†
P09491	0.137	0.001	Q04047-2	0.309	0.003	M9PE32	3.822	0.017
Q9W366	2.543	0.001	Q0E9E2	0.439	0.003	Q9VZY3	3.822	0.017
Q8SZK9	0.277	0.001	Q7KN97	0.439	0.003	Q9VCA8	0.432	0.017
Q9W1W4	8.363	0.001	Q9W334	0.245	0.003	A8DZ09	0.197	0.018
P06002	2.135	0.001	X2JEM4	0.245	0.003	M9PBC6	0.197	0.018
B1P5P0	2.591	0.001	Q9U1K7	0.250	0.004	M9PG25	0.197	0.018
B1P5P1	2.591	0.001	P50887	5.169	0.004	Q8SX73	0.197	0.018
B1P5P2	2.591	0.001	Q9W3N9	0.360	0.004	Q9VJI6	0.197	0.018
E1JGW3	2.591	0.001	P91927	0.470	0.004	X2JED0	0.197	0.018
Q0E8W5	2.591	0.001	Q9VTC3	3.531	0.004	M9PFF1	0.406	0.018
Q9W151	2.591	0.001	Q9VZ01	2.178	0.004	O18358	0.406	0.018
O97477	0.065	0.001	Q9W0G1	3.931	0.004	Q9VTX5	0.406	0.018
Q8IGD8	5.972	0.001	Q9W0G1-2	3.931	0.004	Q9W258	2.355	0.018
Q9W257	5.972	0.001	Q9W0G1-3	3.931	0.004	O18332	0.215	0.018
Q9VIQ0	2.887	0.001	Q9VV29	2.372	0.004	Q9VV28	3.173	0.018
B7Z002	0.203	0.001	Q9VV30	2.372	0.004	Q9VH69	0.335	0.019
M9NCY5	0.203	0.001	M9PBJ1	0.251	0.004	B5X542	0.335	0.019
M9NDB7	0.203	0.001	M9PE03	0.251	0.004	Q7KUT5	0.335	0.019
M9NEL3	0.203	0.001	Q0E8J3	0.251	0.004	Q9VW41	0.335	0.019
Q9VPL9	0.203	0.001	Q0E8J4	0.251	0.004	Q9VI64	0.329	0.019
Q9VV36	0.475	0.001	Q3KN34	0.251	0.004	X2JAU8-7	2.136	0.019
Q27331	0.394	0.001	Q7Z1Y4	0.251	0.004	X2JAU8	2.136	0.019
Q8IR41	2.160	0.001	Q9W053	0.251	0.004	Q8SZA8	2.268	0.020
Q9VXX6	2.160	0.001	A8YPP6	2.752	0.004	A0A0B4KGY6-10	0.441	0.020
Q58CJ5	2.422	0.001	A8YPP9	2.752	0.004	A0A0B4KGY6-11	0.441	0.020
Q9VGN4	2.422	0.001	A8YPQ0	2.752	0.004	A0A0B4KGY6-12	0.441	0.020
Q9V931	3.268	0.001	A8YPQ3	2.752	0.004	A0A0B4KGY6-2	0.441	0.020
Q9VA32	0.267	0.001	A8YPQ4	2.752	0.004	A0A0B4KGY6-3	0.441	0.020
Q9VU84	0.126	0.001	E1JGP3	2.752	0.004	A0A0B4KGY6-4	0.441	0.020
A0A0B4KFY8	0.148	0.001	E1JGP5	2.752	0.004	A0A0B4KGY6-6	0.441	0.020
Q0KI76	0.148	0.001	E1JGP7	2.752	0.004	A0A0B4KGY6-7	0.441	0.020
Q0E8X8	5.183	0.001	E1JGQ0	2.752	0.004	A0A0B4KGY6-8	0.441	0.020
D3DMF7	0.200	0.001	Q9W2N3	2.752	0.004	A1A6X4	0.322	0.020
Q8IPW1	0.200	0.001	Q9W2N4	2.752	0.004	O97118	0.322	0.020
Q8T8Q9	0.200	0.001	Q8SX68	0.249	0.004	Q0ZHI2	0.322	0.020
Q9U3Z9	0.200	0.001	P22464-3	0.138	0.004	Q9W0A8	0.322	0.020

Q9VPU7	0.200	0.001	Q02748	2.244	0.004	Q04499	3.875	0.020
P20228	0.304	0.001	B7Z0J4	0.132	0.004	Q04499-2	3.875	0.020
M9PHM0	3.320	0.001	Q4V485	2.537	0.005	Q04499-3	3.875	0.020
Q9VWD3	3.320	0.001	Q9W2P5	0.260	0.005	Q04499-5	3.875	0.020
Q9Y125	2.167	0.001	Q8T0Q4	2.040	0.005	B7Z0W9	2.176	0.020
Q8MYS9	2.050	0.001	Q9VIL2	0.277	0.005	B7Z0W9-2	2.176	0.020
Q9VLX9	2.050	0.001	C0HL66	0.200	0.005	B7Z0W9-3	2.176	0.020
A1Z784	3.281	0.001	C0HL67	0.200	0.005	Q9VTB4	0.250	0.021
Q7JV23	3.281	0.001	P02299	0.200	0.005	Q24491	0.315	0.021
A1ZAK7	0.141	0.001	Q24583	2.305	0.005	Q9VJC7	0.432	0.021
A1ZAK8	0.141	0.001	Q03427	0.448	0.005	D0UGE6	15.089	0.022
P48593	0.186	0.001	A8JNJ6	0.153	0.005	C0HKA0	0.391	0.022
Q9W114	0.435	0.001	M9PBL6	0.153	0.005	C0HKA1	0.391	0.022
M9PCE0	0.495	0.001	Q7KV69	0.153	0.005	Q9W3U9	0.443	0.022
Q01604	0.495	0.001	Q7KV70	0.153	0.005	X2JAQ5	0.443	0.022
Q9VB51	0.019	0.001	Q9VZQ3	0.153	0.005	Q9VC99	6.061	0.023
Q24212	2.172	0.001	Q9VR59	0.133	0.005	O01367	0.131	0.023
Q8T8R1	0.188	0.001	Q9W596	2.318	0.005	O01367-2	0.131	0.023
Q9VQ35	0.141	0.001	Q868Z9	0.499	0.006	O01367-3	0.131	0.023
A1ZBK7	14.051	0.001	Q868Z9-6	0.499	0.006	Q8SXX2	0.269	0.023
Q9VLP1	2.603	0.001	P19109	0.349	0.006	P48148	0.345	0.024
Q8I0P8	2.049	0.001	P19109-2	0.349	0.006	E2QD98	0.256	0.024
M9PDX2	3.594	0.001	P19109-3	0.349	0.006	M9NEZ2	0.256	0.024
Q8SWU4	3.594	0.001	P19109-4	0.349	0.006	M9NG38	0.256	0.024
Q95SS8	5.519	0.001	Q9VIH9	2.035	0.006	M9PJD0	0.256	0.024
A0A0B4LFW5	4.099	0.001	Q8IPM8	3.138	0.006	X2JCZ1	0.256	0.024
Q9VL18	0.285	0.001	Q8IPM8-3	3.138	0.006	X2JIZ6	0.256	0.024
Q9VAM6	3.036	0.001	A1ZBU8	2.196	0.006	Q961K9	2.722	0.024
A0A6I8WFJ4	5.805	0.001	A4VA47	2.196	0.006	Q9W095	2.722	0.024
Q58L88	5.805	0.001	A4VA48	2.196	0.006	A1Z7H7	0.351	0.024
Q9VC23	2.423	0.001	A4VA49	2.196	0.006	Q8MSK6	0.351	0.024
M9MSK4	2.261	0.001	B5RJ56	2.196	0.006	Q9VSH4	0.423	0.024
Q8IGE3	0.404	0.001	Q8IGD1	2.196	0.006	Q04448	0.175	0.025
Q8IQB7	0.404	0.001	O01404	3.157	0.006	Q04448-2	0.175	0.025
P08928	0.372	0.001	Q9VXT7	0.098	0.006	M9MRD1	0.349	0.025
P38979	0.123	0.001	Q9VQF7	13.945	0.006	Q9VJ68	5.475	0.025
Q7JWD6	2.250	0.001	A1Z7S3	3.735	0.006	Q8IGK8	0.346	0.025
Q7KYI0	2.250	0.001	X2JAU8-4	4.851	0.006	Q9NFP0	0.346	0.025
P16914	0.234	0.001	X2JAU8-6	4.851	0.006	Q9W289	0.346	0.025
P16914-2	0.234	0.001	Q7JR49	0.132	0.006	Q9VYV4	2.369	0.025

Q8MLN7	4.380	0.001	Q9VXB0	4.072	0.006	Q9VPI3	0.446	0.025
Q7YU00	0.233	0.001	Q9VP55	2.493	0.006	P54192	4.175	0.025
Q9VDE9	0.233	0.001	Q9VLP3	2.796	0.006	O96967	0.405	0.027
Q7JZJ3	3.109	0.001	Q9VQ62	0.471	0.006	M9NEV7	3.023	0.027
A1ZB77	0.042	0.001	Q9VCH5	0.485	0.006	Q9VII1	3.023	0.027
Q6IGX9	3.950	0.001	Q9VCH5-2	0.485	0.006	A0A0B4KFA6	4.910	0.027
B5RJE5	4.028	0.001	Q9VII3	4.557	0.006	A0A0B4KGE4	4.910	0.027
Q0KHY8	4.028	0.001	A0ZX43	2.584	0.006	Q8SYR7	4.910	0.027
Q8IGI8	4.028	0.001	A0ZX45	2.584	0.006	Q9VHX9	4.910	0.027
Q8IMJ0	4.028	0.001	A1ZBU5	2.584	0.006	Q9VF08	0.197	0.028
Q7KS11	0.230	0.001	G2J5W3	2.584	0.006	G7H7Z0	0.167	0.028
Q961C4	0.230	0.001	D6W4W6	2.269	0.006	Q8SXM8	0.167	0.028
B3DML7	0.065	0.001	M9PBB3	2.269	0.006	Q9W327	0.167	0.028
Q8SYA8	0.065	0.001	M9PCX0	2.269	0.006	Q9VL70	0.439	0.028
Q9W309	0.065	0.001	M9PFU6	2.269	0.006	Q9VW12	4.941	0.029
Q7JYY0	0.227	0.001	Q7KTB0	2.269	0.006	P36188-3	0.356	0.030
Q9VDH8	0.227	0.001	Q9VJW7	2.269	0.006	D9HRV1	0.184	0.030
A1Z8Z3	3.068	0.001	Q7K3E2	0.383	0.007	D9HRV3	0.184	0.030
D4G7G5	3.068	0.001	Q9V8M5	0.436	0.007	D9HRV4	0.184	0.030
A8JQY2	2.990	0.001	Q8IP62	0.337	0.007	D9HRV5	0.184	0.030
B8A412	2.990	0.001	A1Z968	0.271	0.007	D9HRV8	0.184	0.030
Q8INJ4	2.990	0.001	Q5KTT4	0.271	0.007	D9HRW2	0.184	0.030
Q9VGF8	2.990	0.001	E1JHT6	2.341	0.007	D9HRW5	0.184	0.030
Q24008	0.291	0.002	Q9VMV9	2.341	0.007	D9HRW7	0.184	0.030
Q9VVN2	0.243	0.002	Q86S05	0.271	0.007	D9HRW9	0.184	0.030
Q8IR95	2.111	0.002	Q86S05-2	0.271	0.007	D9HRX4	0.184	0.030
A1Z765	4.430	0.002	Q86S05-3	0.271	0.007	D9HRY4	0.184	0.030
Q8MSH0	4.430	0.002	Q9VC18	0.177	0.007	D9HRZ7	0.184	0.030
Q9VKS1	3.463	0.002	P55841	0.209	0.007	D9HRZ9	0.184	0.030
Q9GQF1	0.101	0.002	Q9W0W6	2.386	0.008	D9HS27	0.184	0.030
Q9GQF1-2	0.101	0.002	Q59DX2	0.284	0.008	F1D8F7	0.184	0.030
C7LA94	0.194	0.002	Q9W002	0.154	0.008	F1D8F9	0.184	0.030
O02649	0.194	0.002	O18640	0.147	0.008	F1D8G2	0.184	0.030
Q9VQ52	9.283	0.002	Q99323	0.299	0.008	F1D8G4	0.184	0.030
A0A0B4KH84	0.175	0.002	Q99323-1	0.299	0.008	F1D8G6	0.184	0.030
Q6NR62	0.175	0.002	Q99323-2	0.299	0.008	F1D8G7	0.184	0.030
Q9VB82	0.175	0.002	Q99323-4	0.299	0.008	F1D8G9	0.184	0.030
Q9VSN3	0.320	0.002	Q8MZC1	0.263	0.008	F1D8H2	0.184	0.030
A1ZA23	0.144	0.002	Q8MRW3	0.124	0.008	H8F4T3	0.184	0.030
Q8T0F0	0.144	0.002	Q9VUN9	0.124	0.008	Q9NDS6	0.184	0.030

Q9U485	0.231	0.002	Q9VLR3	0.020	0.008	Q9VFG5	0.184	0.030
Q9VJ30	0.231	0.002	M9PC73	0.295	0.008	P48603	4.060	0.030
Q8SWS2	3.384	0.002	M9PCD4	0.295	0.008	A8DRW0	2.606	0.031
Q9VYM7	3.384	0.002	M9PCR7	0.295	0.008	P40304	5.375	0.032
X2JEY3	3.384	0.002	Q9VM93	0.295	0.008	Q8MLY8	0.360	0.032
P10379	0.104	0.002	P49630	0.294	0.009	Q8SZM2	0.452	0.032
O46036	0.225	0.002	A1Z877	0.153	0.009	Q86NN8	2.277	0.032
O46036-2	0.225	0.002	Q9W2Y3	0.375	0.009	Q9VMB9	2.277	0.032
M9PCA7	2.942	0.002	Q9VNR6	2.769	0.009	Q9W4Z1	0.200	0.032
M9PI37	2.942	0.002	M9PDP6	0.163	0.009	X2JI50	0.200	0.032
Q9VTW8	2.942	0.002	Q9VPJ0	0.163	0.009	Q9VXA3	2.022	0.033
M9PBA3	2.189	0.002	Q9VZU7	0.228	0.009	Q8MR94	2.486	0.033
Q9W0S5	0.109	0.002	A0A075M5N0	0.144	0.009	Q9W0B3	0.049	0.033
Q9Y0V9	0.109	0.002	M9PD81	0.144	0.009	P39018	0.469	0.033
Q4V5H9	5.267	0.002	M9PGC5	0.144	0.009	A0A0B4KFZ2	0.258	0.033
Q9VZG0	5.267	0.002	M9PIH3	0.144	0.009	Q6AWD5	0.258	0.033
Q9VHX4	0.287	0.002	B5RIJ9	0.295	0.009	Q95SH2	0.067	0.034
B7Z0E0	0.403	0.002	Q8MT23	0.295	0.009	Q9VHS2	0.202	0.034
C8VV61	0.403	0.002	Q9VJ19	0.295	0.009	Q7JR99	0.073	0.034
Q7KUB0	0.403	0.002	Q9GU49	0.147	0.009	Q24048	0.278	0.035
Q7KUB1	0.403	0.002	A0A0B4LFM0	0.457	0.009	Q24048-2	0.278	0.035
Q8IQA7	0.403	0.002	P41044	0.457	0.009	Q8SZD9	6.888	0.035
Q9VSI6	0.403	0.002	Q9VWD9	0.162	0.009	Q9VJH8	6.888	0.035
P61851	2.218	0.002	Q07327	0.379	0.009	B7FNM4	2.520	0.036
P29746	2.741	0.002	Q9W022	2.185	0.010	Q8IRD6	2.520	0.036
P18459	0.126	0.002	A0A0B4KFZ9	0.390	0.010	Q45VV3	0.228	0.037
P18459-2	0.126	0.002	P84040	0.390	0.010	Q45VV3-2	0.228	0.037
P47948	0.218	0.002	Q9V3V6	0.355	0.011	Q9VJZ6	0.374	0.037
P54359	0.321	0.002	A0A0B4KI07	3.075	0.011	Q9W402	0.181	0.038
Q06943	0.413	0.002	A0A0B4KI51	3.075	0.011	P09180	0.482	0.038
Q9VRL0	2.122	0.002	B5X552	3.075	0.011	A1Z9M6	0.471	0.039
P12080	0.251	0.003	Q9VAP9	3.075	0.011	Q1EC07	0.471	0.039
P12080-2	0.251	0.003	A1Z8Y3	0.378	0.011	Q07171	0.461	0.039
P91621	0.500	0.003	Q29QQ5	0.378	0.011	Q07171-2	0.461	0.039
Q9W1F8	2.220	0.003	P10676-2	3.416	0.011	Q07171-6	0.461	0.039
A0A1Z1CN86	2.701	0.003	Q95083	0.403	0.012	P06606	0.095	0.040
Q9VHK7	2.701	0.003	Q7K1V0	2.150	0.012	A1Z7A6	0.212	0.042
Q6NL82	0.476	0.003	P37193	2.200	0.012	A1Z7A6-2	0.212	0.042
Q8IMT8	0.476	0.003	Q9VII5	2.764	0.012	A0A0B4KH25	3.301	0.042
Q9VBY7	0.476	0.003	Q9VMM6-2	2.249	0.012	P08985	3.301	0.042

P13677	3.686	0.003	A0A0B4K6X5	2.338	0.012	Q9VPX6	0.198	0.043
Q05856	0.245	0.003	C1C535	2.338	0.012	X2JAU8-3	3.398	0.044
X2JDH2	2.314	0.003	Q94529	2.085	0.012	C8VUZ1	0.301	0.044
Q9I7S8	0.319	0.003	Q9VTZ6	0.107	0.012	E8NH92	0.301	0.044
Q9VJJ0	2.375	0.003	Q9W0S7	0.203	0.012	Q8IRH0	0.301	0.044
Q9V9M7	0.286	0.003	A0A0B4KEW6	2.895	0.013	Q8IRH1	0.301	0.044
P08736	0.190	0.003	Q9VSH5	2.306	0.013	Q9W0E4	0.301	0.044
Q9V359	0.192	0.003	Q9NFZ3	2.798	0.013	Q9VKQ9	3.801	0.045
A0A0B4LFW8	0.126	0.003	Q9VSY0	2.226	0.013	Q9VMV5	0.475	0.045
Q7JUV6	0.126	0.003	Q08012	0.412	0.013	Q7K206	0.118	0.045
Q9VLP2	3.190	0.003	Q9VPC2	2.032	0.014	Q9VSL4	0.118	0.045
Q9W266	2.832	0.003	P05389	0.369	0.015	P25455	2.405	0.045
P36975	2.121	0.003	Q9NB04	0.313	0.015	P25455-1	2.405	0.045
P25171	0.265	0.003	P48610	2.699	0.016	P25455-2	2.405	0.045
A1Z935	4.153	0.003	Q9W2X6	2.107	0.016	P25455-4	2.405	0.045
A1Z936	4.153	0.003	Q7KU06	2.165	0.016	P25455-5	2.405	0.045
Q8SZB0	4.153	0.003	Q8SYK1	2.165	0.016	P25455-6	2.405	0.045
Q9VGQ1	4.392	0.003	O46037	0.381	0.016	Q9U616	2.477	0.046
P29843	0.262	0.003	Q95RJ9	0.170	0.016	Q8MQL9	0.266	0.047
Q8T0I9	0.235	0.003	Q95RY2	0.417	0.016	Q9VXU3	0.266	0.047
Q968S4	0.235	0.003	Q9VKQ2	3.641	0.016	Q9VW26	0.379	0.048
Q9U3W8	0.235	0.003	B7YZN8	2.769	0.016	Q9VV46	0.177	0.048
Q9VM97	0.235	0.003	Q32KE4	2.373	0.017	Q7KTB3	0.376	0.049
E0R953	0.247	0.003	Q9VZZ1	2.373	0.017	Q8IP79	0.376	0.049
Q4V615	0.247	0.003	P62152	2.328	0.017	Q8IP80	0.376	0.049
Q9VYC9	0.247	0.003	Q9W227	0.238	0.017	Q9VK25	0.376	0.049
P29327	0.192	0.003	Q00174	0.392	0.017	Q9V8Y9	2.946	0.049
Q04047	0.309	0.003	M9PDV6	3.822	0.017			

§UniProt; ¶, Cutoff ±2; 3, p-value generated by Student's t-test.

Table S6. Differentially regulated proteins in IAA-fed female flies (IAAF) as compared to untreated female flies (CF).

Protein§	Fold change¶	p-Value†	Protein§	Fold change¶	p-Value†	Protein§	Fold change¶	p-Value†
Q9VKK5	2.867	0.001	Q24595-3	2.028	0.002	X2JAU8-4	6.241	0.012
Q9VHC8	2.049	0.001	Q9VB51	0.042	0.002	X2JAU8-6	6.241	0.012
A1Z765	2.783	0.001	Q8SYA7	2.806	0.002	A0A9F2GM10	2.166	0.012
Q8MSH0	2.783	0.001	Q6NP53	0.092	0.003	A0A9F2H0X6	2.166	0.012
Q24583	2.498	0.001	Q9VLX0	0.092	0.003	A0A9F2H0X7	2.166	0.012
P36188	2.661	0.001	P19351-13	2.168	0.003	A0A9F2H0X8	2.166	0.012
P29746	3.772	0.001	Q26416	4.665	0.003	A8JNI2	2.166	0.012
P48603	45.520	0.001	A1ZB77	0.062	0.003	Q7YZ99	2.166	0.012
Q9VQK7	2.262	0.001	H7BWR4	0.070	0.003	Q86BP9	2.166	0.012
Q9VIQ8	2.783	0.001	H7BWS2	0.070	0.003	Q960C4	2.166	0.012
B1P5P0	2.499	0.001	H7BWS3	0.070	0.003	Q9W0B0	2.166	0.012
B1P5P1	2.499	0.001	H7BWS6	0.070	0.003	X2J8K5	2.166	0.012
B1P5P2	2.499	0.001	H7BWS7	0.070	0.003	Q8IGS1	8.500	0.012
E1JGW3	2.499	0.001	H7BWU6	0.070	0.003	Q9VEN3	8.500	0.012
Q0E8W5	2.499	0.001	H7BWU8	0.070	0.003	A1ZAK7	0.221	0.012
Q9W151	2.499	0.001	Q2QBM1	0.070	0.003	A1ZAK8	0.221	0.012
P05389	0.373	0.001	Q9NIW0	0.070	0.003	Q7JX94	2.136	0.012
P48809	0.466	0.001	Q9NIW1	0.070	0.003	Q9VVI2	0.134	0.013
Q9VTY2	0.485	0.001	Q9NIW2	0.070	0.003	F3YDN9	3.153	0.013
Q9VSY0	3.206	0.001	Q9U1J1	0.070	0.003	M9PGW5	3.153	0.013
Q9VVI9	2.363	0.001	Q9VG31	0.070	0.003	O76869	3.153	0.013
Q6NLJ9	2.849	0.001	Q9VG32	0.070	0.003	Q8I072	3.153	0.013
A0A1B2AJW2	4.591	0.001	Q9VC30	5.831	0.003	Q8I081	3.153	0.013
P41964	4.591	0.001	Q95SH2	0.092	0.003	Q8I097	3.153	0.013
Q9VIQ5	3.054	0.001	P06603	3.322	0.003	Q8I6B8	3.153	0.013
Q8IGE3	0.327	0.001	P06605	3.322	0.003	Q8I6B9	3.153	0.013
Q8IQB7	0.327	0.001	Q7KT70	2.864	0.003	Q8I6C0	3.153	0.013
Q9VLP1	3.704	0.001	Q7YU32	2.864	0.003	Q8I6C1	3.153	0.013
Q9W0B3	0.062	0.001	Q9NK80	2.864	0.003	Q8I6C2	3.153	0.013
Q9VEY0	0.400	0.001	M9PAZ4	4.069	0.003	Q8I6C3	3.153	0.013
Q24008	0.455	0.001	Q59E11	4.069	0.003	Q8I6C4	3.153	0.013
C7LA72	2.516	0.001	Q7YTY8	4.069	0.003	Q8I6C6	3.153	0.013
Q8IQX3	2.516	0.001	Q9VPV8	4.069	0.003	Q8I6C7	3.153	0.013
A9J7N9	0.497	0.001	Q23970	0.420	0.003	Q8I6C8	3.153	0.013
Q4QPQ0	0.497	0.001	P47949	11.502	0.003	Q8IRV2	3.153	0.013
Q9VLC5	0.497	0.001	X2JCI6	11.502	0.003	Q8IRV3	3.153	0.013

Q8T0L3	0.252	0.001	A1ZBK7	15.748	0.003	Q9W4V1	3.153	0.013
Q9VT04	2.322	0.001	M9PFR6	0.442	0.003	C6TP67	4.644	0.013
M9PFX7	2.065	0.001	Q9XZ03	0.442	0.003	E1JGT3	4.644	0.013
Q8MYU6	2.065	0.001	C0P8N1	2.968	0.003	Q9W1R6	4.644	0.013
Q9VW17	2.065	0.001	Q9W022	2.065	0.003	A1Z9U2	6.880	0.014
P02515	4.969	0.001	Q8MYS9	4.909	0.003	Q8SX39	6.880	0.014
P19334	2.313	0.001	Q9VLX9	4.909	0.003	C0HK92-2	0.109	0.014
Q9Y125	2.215	0.001	O16157	2.177	0.003	P09491	0.112	0.014
B7Z002	0.379	0.001	Q8MSI2	2.177	0.003	Q9W335	3.334	0.014
M9NCY5	0.379	0.001	M9MSK4	3.138	0.003	Q9VP57	0.321	0.014
M9NDB7	0.379	0.001	O62621	5.130	0.003	Q9VLY1	2.929	0.014
M9NEL3	0.379	0.001	Q9VTA8	11.283	0.003	Q9VZ23	7.923	0.014
Q9VPL9	0.379	0.001	Q9W3L4	0.445	0.003	Q9W5N2	0.206	0.015
P54192	3.651	0.001	Q9VCU0	2.834	0.003	M9NEN9	2.611	0.015
Q26365-2	2.112	0.001	Q9VW26	0.174	0.004	Q7KU08	2.611	0.015
Q9VAA6	2.423	0.001	P91927	0.490	0.004	Q9VQ47	2.611	0.015
Q9VXY5	14.867	0.001	A0A0B4KI23	43.664	0.004	Q9VMT2	3.780	0.015
Q9VZ01	2.633	0.001	Q9VAE5	43.664	0.004	X2JD70	3.780	0.015
Q9VUV8	4.215	0.001	Q9U1K3	0.434	0.004	Q0E8V7	0.414	0.015
Q9VH76	2.134	0.001	Q9VF15	0.434	0.004	Q9XZ19	0.120	0.015
Q9W1X9	3.052	0.001	Q7JV39	2.234	0.004	Q9VQT8	7.851	0.016
Q8IR41	2.490	0.001	Q9V4N3	4.652	0.004	Q960M4	0.150	0.016
Q9VXX6	2.490	0.001	Q9V4N3-2	4.652	0.004	A0ANY9	3.653	0.016
Q9VZL1	2.607	0.001	A0A0B4K6N4	2.055	0.004	A0ANZ0	3.653	0.016
Q7K3W4	2.100	0.001	A0A0B4K7R6	2.055	0.004	Q9U5W4	3.653	0.016
Q24211	2.050	0.001	A0A0B4KHF2	2.055	0.004	Q9VM69	3.653	0.016
O97365	2.169	0.001	A0A0B4KHW6	2.055	0.004	A9YK67	6.608	0.016
Q24212	2.598	0.001	A0A6H2EEH6	2.055	0.004	A9YK77	6.608	0.016
P45843	3.015	0.001	A8JRH3	2.055	0.004	Q9W3B3	6.608	0.016
Q9W1G0	0.224	0.001	Q6IDG5	2.055	0.004	Q9VDH3	3.432	0.017
Q95RA9	0.321	0.001	Q9VA59	2.055	0.004	Q9VRL0	2.552	0.017
Q9VP53	7.009	0.001	Q9W1F8	2.837	0.004	Q9VHP0	0.128	0.018
P29844	0.471	0.001	M9PFZ6	0.183	0.004	Q9I7K0-2	2.473	0.019
Q9I7U4-5	7.671	0.001	P02574	0.183	0.004	H0RN81	0.283	0.019
Q9V9U7	2.227	0.001	X2JEU5	2.265	0.004	P48375	0.283	0.019
Q8SYD9	0.478	0.001	Q9VYD7	2.265	0.004	O18373	0.144	0.020
O97064	4.378	0.001	A1Z8H1	3.874	0.004	Q24201	2.158	0.020
Q8MRD2	4.378	0.001	Q29QJ2	3.874	0.004	Q8IH63	2.158	0.020
P13677	5.577	0.001	Q9VR07	2.644	0.005	Q9VIB5	2.158	0.020
Q9VC44	4.972	0.001	Q9V4C8	2.227	0.005	Q8SY67	2.418	0.020

Q9VIQ0	5.608	0.001	Q9V4C8-5	2.227	0.005	A0A0B4KF06	4.421	0.020
M9PG20	13.249	0.001	Q9V4C8-6	2.227	0.005	Q0E993	4.421	0.020
M9PG82	13.249	0.001	A0A0B4LFW3	3.719	0.005	Q960E6	4.421	0.020
M9PID3	13.249	0.001	A9QHS4	3.719	0.005	T2GFI6	4.421	0.020
Q9VPB0	13.249	0.001	A9QHS6	3.719	0.005	Q8MRF0	3.496	0.020
A1Z8H6	3.347	0.001	A9QHW8	3.719	0.005	Q9VIK0	3.496	0.020
P54357	2.190	0.001	C0PV28	3.719	0.005	Q9V813	0.132	0.020
Q9W0C1	3.822	0.001	Q7K088	3.719	0.005	Q8SX68	0.136	0.021
Q9W0C1-2	3.822	0.001	Q9TVP3	2.514	0.005	Q9VJQ3	0.452	0.021
Q9W0C1-3	3.822	0.001	Q9TVP3-2	2.514	0.005	P07701	0.128	0.021
Q9W0C1-4	3.822	0.001	X2JAU8-7	2.420	0.005	P06606	0.135	0.021
Q9W0C1-5	3.822	0.001	X2JAU8	2.420	0.005	Q9Y1A3	0.483	0.021
Q9VM18	0.449	0.001	Q24478	2.664	0.005	X2JB87	0.483	0.021
Q8SXE1	15.704	0.001	Q9VIT5	6.227	0.005	Q9V426	0.491	0.021
Q9V3W7	0.325	0.001	Q9VX77	7.500	0.005	Q8SY69	7.375	0.022
Q59E04	10.377	0.001	X2JAW9	11.083	0.005	Q24147	0.174	0.022
Q59E04-2	10.377	0.001	A0A126GUV7	0.085	0.005	Q7K180	0.174	0.022
Q9VKQ9	6.639	0.001	Q7KSD3	0.085	0.005	Q9VL68	0.132	0.022
P35128	3.807	0.001	Q9VE62	0.085	0.005	A0A0B4K7A5	3.413	0.022
P16914	0.411	0.001	Q9W2F6	7.574	0.005	A0A0B4K849	3.413	0.022
P16914-2	0.411	0.001	Q9VAC4	2.457	0.005	P40423	0.143	0.022
Q4QQA4	3.130	0.001	A1Z979	0.155	0.006	Q9VQ88	5.649	0.024
Q9VWL3	3.130	0.001	C1C5A7	0.155	0.006	Q9VTZ6	0.153	0.024
P54359	0.392	0.001	Q4V4H7	0.155	0.006	A0A075M5N0	0.266	0.025
M9PEG1	2.149	0.001	Q9VMM6-2	2.913	0.006	M9PD81	0.266	0.025
B7FNM4	3.149	0.001	Q8T0Q4	2.370	0.006	M9PGC5	0.266	0.025
Q8IRD6	3.149	0.001	Q9V9M7	0.419	0.006	M9PIH3	0.266	0.025
Q9VKK1	0.056	0.001	P47948	0.360	0.006	P55830	0.379	0.026
P91941	3.620	0.001	Q09103-3	0.494	0.006	Q8MLN7	3.494	0.026
Q7JND6	3.620	0.001	P36975	3.228	0.006	Q9VSA9	0.431	0.026
E1JHT6	2.565	0.001	Q8MSI7	2.298	0.006	Q9VU84	0.257	0.027
Q9VMV9	2.565	0.001	Q9VQD7	2.298	0.006	A0A0B4KFZ9	0.230	0.028
A0A0B4KEW6	2.812	0.001	A0A0B4KHJ9	0.169	0.006	P84040	0.230	0.028
B7Z0E0	0.365	0.001	P09491-2	0.169	0.006	A0A0B4LGS4	0.388	0.028
C8VV61	0.365	0.001	Q7KUQ6	2.238	0.006	P54611	0.388	0.028
Q7KUB0	0.365	0.001	Q9W552	2.315	0.006	Q9W3N6	0.170	0.029
Q7KUB1	0.365	0.001	G2J5Z0	2.423	0.006	Q7K860	0.394	0.030
Q8IQA7	0.365	0.001	P21521	2.111	0.006	Q8SZM6	0.394	0.030
Q9VSI6	0.365	0.001	P21521-2	2.111	0.006	Q9VQ35	0.276	0.030
A0A0B4K891	0.464	0.001	P21521-4	2.111	0.006	Q7JUS9	2.387	0.030

Q7K581	0.464	0.001	A0A0B4KHH8	0.077	0.006	Q5BIC6	0.151	0.030
A8Y4V5	3.064	0.001	A0A0B4KHI4	0.077	0.006	Q8IQM9	0.151	0.030
A1ZA47-4	0.329	0.001	A0A0B4KHT5	0.077	0.006	C4NYP8	0.464	0.030
A1ZA47-5	0.329	0.001	Q7KS11	0.403	0.006	E2QD63	0.464	0.030
Q8IM93	2.152	0.001	Q961C4	0.403	0.006	Q86DS1	0.464	0.030
C9QPI3	13.603	0.001	A0A0B4KFZ2	0.368	0.007	M9PDP6	0.272	0.031
Q961H6	13.603	0.001	Q6AWD5	0.368	0.007	Q9VPJ0	0.272	0.031
Q9VM10	13.603	0.001	Q9VKQ0	2.718	0.007	Q9VXY7	2.052	0.032
Q0E8X8	4.278	0.002	Q8IGV1	2.162	0.007	Q9VCK1	0.272	0.033
Q8IR95	2.576	0.002	Q8IMV6	2.162	0.007	Q7K206	0.166	0.033
Q9V931	4.169	0.002	Q9VQT7	2.272	0.007	Q9VSL4	0.166	0.033
P06002	3.536	0.002	Q6NND1	5.245	0.007	Q9VKQ2	5.033	0.033
Q04047	0.376	0.002	Q8IMC1	5.245	0.007	Q8T9H4	2.354	0.034
Q04047-2	0.376	0.002	Q9V4E7	5.245	0.007	Q9VIN9	0.180	0.035
M9PC74	2.808	0.002	A0A0B4KG37	2.240	0.007	Q9VGQ1	2.163	0.036
Q7K1W8	2.808	0.002	Q9VET0	2.240	0.007	Q05856	0.167	0.036
Q8MZC1	0.167	0.002	D3DMF7	0.358	0.008	A8YPP6	3.339	0.036
A4VA25	5.561	0.002	Q8IPW1	0.358	0.008	A8YPP9	3.339	0.036
Q8SY78	5.561	0.002	Q8T8Q9	0.358	0.008	A8YPQ0	3.339	0.036
Q9VIQ6	5.561	0.002	Q9U3Z9	0.358	0.008	A8YPQ3	3.339	0.036
Q9VTU2	3.053	0.002	Q9VPU7	0.358	0.008	A8YPQ4	3.339	0.036
B7Z0W9	2.122	0.002	O02195	0.478	0.008	E1JGP3	3.339	0.036
B7Z0W9-2	2.122	0.002	Q9W330	2.128	0.008	E1JGP5	3.339	0.036
B7Z0W9-3	2.122	0.002	Q8SY61	2.900	0.008	E1JGP7	3.339	0.036
Q9U1L3	3.664	0.002	Q9VZA4	0.457	0.008	E1JGQ0	3.339	0.036
Q9W5C2	3.664	0.002	B3DML7	0.120	0.008	Q9W2N3	3.339	0.036
Q6NL87	5.955	0.002	Q8SYA8	0.120	0.008	Q9W2N4	3.339	0.036
Q8I0D4	5.955	0.002	Q9W309	0.120	0.008	P06754-1	2.210	0.037
Q8MQM4	5.955	0.002	Q8SXV4	4.074	0.008	Q9VWD9	0.227	0.037
Q9VFR2	5.955	0.002	Q9VDD2	3.649	0.008	Q9W2X6	2.018	0.038
A4VA55	2.799	0.002	A1ZB23	2.289	0.008	A0A1B2AIW6	0.386	0.038
C7LAB3	2.106	0.002	Q9XZS3	2.182	0.008	P42281	0.386	0.038
D0IQD2	2.106	0.002	Q8IGF6	4.278	0.008	P18431	3.354	0.038
Q9W3X7	2.106	0.002	Q7K5J8	2.044	0.009	P18431-3	3.354	0.038
B7FNM8	2.777	0.002	P41094	0.101	0.009	Q9I7S8	0.290	0.039
C3KGQ8	2.777	0.002	Q9W0G1	2.633	0.009	A0A0B4K7G4	2.311	0.039
M9MS70	2.777	0.002	Q9W0G1-2	2.633	0.009	P13469	2.311	0.039
A0A1Z1CN86	3.486	0.002	Q9W0G1-3	2.633	0.009	Q94522	0.315	0.039
Q9VHK7	3.486	0.002	Q9VKG4	2.097	0.009	Q500Y7	0.179	0.040
M9PBA3	2.952	0.002	Q7JZV0	11.136	0.009	C7LA94	0.258	0.040

A4VA91	2.882	0.002	X2JCY4	2.029	0.009	O02649	0.258	0.040
Q8SY60	2.882	0.002	Q9W2I2	2.832	0.009	Q9VGA3	0.345	0.040
Q9VK39	2.882	0.002	Q23709	2.082	0.009	Q9W2P5	0.297	0.041
Q04499	7.956	0.002	Q9V9U0	2.082	0.009	O01367	0.173	0.041
Q04499-2	7.956	0.002	Q5U108	0.405	0.010	O01367-2	0.173	0.041
Q04499-3	7.956	0.002	Q8I937	0.405	0.010	O01367-3	0.173	0.041
Q04499-5	7.956	0.002	Q8MR24	0.405	0.010	P29843	0.293	0.041
Q9VQ52	11.523	0.002	Q8SYQ4	2.261	0.010	Q02748	2.537	0.041
Q8I0P8	3.336	0.002	Q8SWS2	3.550	0.010	E1JIJ5	0.308	0.043
P26308	3.017	0.002	Q9VYM7	3.550	0.010	P31409	0.308	0.043
A0A1B2AJI5	0.296	0.002	X2JEY3	3.550	0.010	Q7KTP7	0.178	0.043
Q27377	0.296	0.002	Q7KV34	4.051	0.010	M9PCE0	0.496	0.043
Q9VAY2	0.047	0.002	Q94519-2	2.085	0.010	Q01604	0.496	0.043
A1Z8Z3	4.026	0.002	Q4V5I9	4.207	0.010	P62152	3.179	0.045
D4G7G5	4.026	0.002	P37193	2.511	0.010	Q9VYY7	4.808	0.045
A0A0B4K7C1	2.226	0.002	Q7K533	2.066	0.010	F0JAK8	0.182	0.045
A0A0B4KFS5	2.226	0.002	Q4V498	4.597	0.010	Q7K7G0	0.182	0.045
D1Z397	2.226	0.002	Q4V4B8	4.597	0.010	Q9U8A9	0.182	0.045
Q7KHE2	2.226	0.002	Q8I929	4.597	0.010	Q9W555	0.182	0.045
Q9GQ82	2.226	0.002	Q9VR48	4.597	0.010	Q9W4A6	2.511	0.046
Q9VSH5	3.107	0.002	P22700-2	2.243	0.010	O76454	2.013	0.046
Q9VAA9	0.046	0.002	A1A6X4	0.491	0.011	P91926	0.188	0.047
Q9VV29	2.747	0.002	O97118	0.491	0.011	Q8SXG6	0.191	0.047
Q9VV30	2.747	0.002	Q0ZHI2	0.491	0.011	Q9VZH5	0.191	0.047
Q9NJH0	2.417	0.002	Q9W0A8	0.491	0.011	E2QCY9	2.300	0.047
Q9W306	2.626	0.002	E0R953	0.110	0.011	E2QCZ0	2.300	0.047
Q9I7Q5	7.308	0.002	Q4V615	0.110	0.011	O01404	3.004	0.049
Q9VW12	3.872	0.002	Q9VYC9	0.110	0.011	Q9V438	0.204	0.049
M9NEV7	2.431	0.002	M9PDL2	2.707	0.011	Q9VHX4	0.188	0.049
Q9VII1	2.431	0.002	Q9VJH2	2.707	0.011	P54622	0.188	0.049
Q24595	2.028	0.002	O97477	0.099	0.012	Q9VYY3	0.199	0.049
						Q9VSY6	0.190	0.050

§UniProt; ¶, Cutoff ±2; 3, p-value generated by Student's t-test.

Table S7. Differentially regulated proteins in IAA-fed male flies (IAAM) as compared to untreated male flies (CM).

Protein§	Fold change¶	p-Value†	Protein§	Fold change¶	p-Value†	Protein§	Fold change¶	p-Value†
Q03427	0.388	0.001	Q961K9	9.461	0.006	Q9VZR2	2.123	0.017
P40304	13.472	0.001	Q9W095	9.461	0.006	Q9VJJ0	3.237	0.017
Q9W334	0.163	0.001	Q86NN8	2.860	0.006	Q0E9E2	0.451	0.017
X2JEM4	0.163	0.001	Q9VMB9	2.860	0.006	Q7KN97	0.451	0.017
A8DRW0	2.443	0.001	Q01617-6	0.380	0.006	Q9VFS8	0.485	0.018
A1ZBK7	12.779	0.001	M9PCE0	0.493	0.006	Q2PDQ6	3.493	0.018
Q9W2X6	2.189	0.001	Q01604	0.493	0.006	Q9I7S8	0.345	0.018
P15215	0.450	0.001	Q9VNR6	2.221	0.006	Q8MR94	2.377	0.018
Q9VFI3	2.153	0.001	Q9VDS5	6.269	0.006	M9PFZ6	0.445	0.018
Q9VX35	0.491	0.001	Q9W114	0.291	0.006	P02574	0.445	0.018
X2JFW6	0.491	0.001	Q9VSP9	0.300	0.007	C0HK94	0.483	0.018
P22979	0.260	0.001	Q27331	0.241	0.007	Q23970	2.433	0.018
Q8SY61	2.553	0.001	Q9VQ52	7.932	0.007	Q7JXF7	0.402	0.019
O96967	0.313	0.001	O62619	0.375	0.007	Q9VU43	0.082	0.020
Q9VV36	0.486	0.001	O62619-2	0.375	0.007	Q95SS8	2.928	0.021
P13395	0.339	0.001	P05661-25	0.368	0.007	Q9VV29	2.100	0.021
P02515	18.374	0.001	P05661-26	0.368	0.007	Q9VV30	2.100	0.021
P48596	0.078	0.001	Q9VB81	0.083	0.007	Q9V535	0.084	0.021
Q09103-3	0.370	0.001	Q9VQF7	8.651	0.007	Q9VQT7	2.199	0.022
A0A1B3Q3N5	3.077	0.001	B7YZN8	2.649	0.007	B5RJE5	2.020	0.022
P48588	3.077	0.001	P49455	0.500	0.007	Q0KHY8	2.020	0.022
A1Z8G7	3.085	0.001	A0A0B4KI07	2.987	0.007	Q8IGI8	2.020	0.022
A4V9Y5	3.085	0.001	A0A0B4KI51	2.987	0.007	Q8IMJ0	2.020	0.022
Q8MYT6	3.085	0.001	B5X552	2.987	0.007	Q8SZN1	2.366	0.022
Q9VM18	0.266	0.002	Q9VAP9	2.987	0.007	Q9Y125	2.115	0.023
C0HK95	0.367	0.002	Q9VTY2	0.371	0.008	Q7K5M6	0.369	0.023
P40421	8.347	0.002	F2FBB9	0.234	0.008	Q8SX68	0.351	0.026
P55841	0.104	0.002	Q8IPD8	0.234	0.008	Q9VII5	2.803	0.026
Q9VLP2	3.670	0.002	M9PFE5	0.340	0.008	P39018	0.375	0.027
C4NAP6	2.403	0.002	M9PFI2	0.340	0.008	Q9VCR9	2.427	0.027
F3YD60	2.403	0.002	Q9VU76	0.340	0.008	Q9VKS1	2.933	0.028
Q9W077	0.474	0.002	Q9NHE5	6.295	0.008	Q9VGS2	0.476	0.028
P19351-12	0.486	0.002	Q9NHE5-2	6.295	0.008	Q7JRN6	2.290	0.028
M9MRX0	0.478	0.002	Q9NHE5-4	6.295	0.008	Q9VLP3	2.990	0.029

A1ZA47-4	0.356	0.002	Q9NHE5-5	6.295	0.008	Q9U616	3.019	0.030
A1ZA47-5	0.356	0.002	Q9NHE5-6	6.295	0.008	A0A0B4KEW6	2.963	0.031
Q9VXF9	0.422	0.003	Q9NHE5-7	6.295	0.008	Q9VIE8	0.269	0.031
Q24583	2.148	0.003	Q8SYD9	0.393	0.008	M9PCA7	4.096	0.032
Q23982	0.029	0.003	Q9V8Y9	3.992	0.008	M9PI37	4.096	0.032
A1Z765	9.473	0.003	O01404	3.295	0.008	Q9VTW8	4.096	0.032
Q8MSH0	9.473	0.003	Q9VYV4	2.377	0.008	Q26377	2.447	0.032
X2JEU5	2.800	0.003	Q7JWD6	2.884	0.009	A8DY49	3.086	0.032
Q9VYD7	2.800	0.003	Q7KYI0	2.884	0.009	A9UNH4	3.086	0.032
P91621	0.486	0.003	A1Z8H1	2.316	0.009	P22464-3	0.059	0.032
P10676-2	2.793	0.003	Q29QJ2	2.316	0.009	B3DNG2	3.724	0.032
P36188-9	0.337	0.003	F7VJS8	3.651	0.009	E1JIS4	3.724	0.032
P08736	0.264	0.004	Q6IJE8	3.651	0.009	P25455	3.820	0.032
A0A0B4K7E3	0.448	0.004	Q9W0G1	6.797	0.009	P25455-1	3.820	0.032
A0A0B4K7M0	0.448	0.004	Q9W0G1-2	6.797	0.009	P25455-2	3.820	0.032
A0A6M3Q9L5	0.448	0.004	Q9W0G1-3	6.797	0.009	P25455-4	3.820	0.032
F0JAJ0	0.448	0.004	Q9VMT2	2.588	0.009	P25455-5	3.820	0.032
Q0E8Y1	0.448	0.004	X2JD70	2.588	0.009	P25455-6	3.820	0.032
Q8T0K5	0.448	0.004	Q9VQT8	2.252	0.010	B5X535	4.114	0.033
Q9W1Y2	0.448	0.004	Q9VJE5	0.483	0.010	Q9VCR4	4.114	0.033
Q9W1Y3	0.448	0.004	Q9VJE5-2	0.483	0.010	Q9VII3	4.789	0.034
Q8T8R1	0.264	0.004	A0AQ24	0.444	0.011	A0A0B4KH84	0.126	0.034
Q06943	0.458	0.004	Q9VQR2	0.444	0.011	Q6NR62	0.126	0.034
Q9VA32	0.135	0.004	P56079	3.461	0.011	Q9VB82	0.126	0.034
Q86B87	0.383	0.004	Q9VMW7	0.416	0.011	B7FNM4	2.239	0.034
Q86B87-10	0.383	0.004	Q9VV46	0.112	0.011	Q8IRD6	2.239	0.034
Q86B87-11	0.383	0.004	Q9VC30	6.532	0.011	Q9I7Q5	4.291	0.034
Q86B87-12	0.383	0.004	Q0E8X8	5.997	0.011	Q9VDH3	4.311	0.034
Q86B87-13	0.383	0.004	Q9VIH9	2.592	0.011	Q9V9M7	0.064	0.034
Q86B87-14	0.383	0.004	Q9V4N3	5.349	0.012	P42325	0.459	0.034
Q86B87-15	0.383	0.004	Q9V4N3-2	5.349	0.012	P46150	0.390	0.034
Q86B87-16	0.383	0.004	Q8MLN7	5.157	0.012	P46150-2	0.390	0.034
Q86B87-17	0.383	0.004	Q02427	0.163	0.012	P46150-3	0.390	0.034
Q86B87-18	0.383	0.004	Q02427-1	0.163	0.012	P46150-4	0.390	0.034
Q86B87-19	0.383	0.004	O01666	0.455	0.012	P46150-5	0.390	0.034
Q86B87-2	0.383	0.004	Q9VG51	0.391	0.013	Q9VMD0	3.888	0.034
Q86B87-20	0.383	0.004	Q8IPA4	3.848	0.013	Q6NL87	2.590	0.034
Q86B87-21	0.383	0.004	P36188-5	0.393	0.013	Q8I0D4	2.590	0.034
Q86B87-22	0.383	0.004	Q24395	5.939	0.013	Q8MQM4	2.590	0.034
Q86B87-23	0.383	0.004	P61851	2.568	0.014	Q9VFR2	2.590	0.034

Q86B87-24	0.383	0.004	Q0E8H8	3.574	0.014	Q9VXB0	3.802	0.035
Q86B87-25	0.383	0.004	Q3HKQ2	3.574	0.014	B7Z0W9	2.221	0.036
Q86B87-26	0.383	0.004	Q8T964	3.574	0.014	B7Z0W9-2	2.221	0.036
Q86B87-27	0.383	0.004	Q9VTA8	2.719	0.014	B7Z0W9-3	2.221	0.036
Q86B87-28	0.383	0.004	Q9VBL6	4.543	0.014	Q9VAC4	2.080	0.036
Q86B87-29	0.383	0.004	Q27268	3.796	0.014	H0RN81	0.178	0.037
Q86B87-3	0.383	0.004	Q7K326	4.678	0.014	P48375	0.178	0.037
Q86B87-30	0.383	0.004	Q9VHF9	4.678	0.014	A0A0B4KGT7	0.315	0.038
Q86B87-31	0.383	0.004	C6TP67	2.880	0.014	P61849	0.315	0.038
Q86B87-4	0.383	0.004	E1JGT3	2.880	0.014	Q95RB2	0.178	0.040
Q86B87-5	0.383	0.004	Q9W1R6	2.880	0.014	M9PDX2	2.547	0.040
Q86B87-6	0.383	0.004	Q9V931	2.658	0.014	Q8SWU4	2.547	0.040
Q86B87-7	0.383	0.004	Q95NH6	4.888	0.015	Q9VSR5	0.445	0.041
Q86B87-8	0.383	0.004	Q8IPM8	3.191	0.015	Q0E8J0	2.060	0.041
Q86B87-9	0.383	0.004	Q8IPM8-3	3.191	0.015	Q3KN57	2.060	0.041
Q9VTC3	4.728	0.004	Q9W258	2.989	0.015	A0A1B2AIV9	0.310	0.042
M9MRY7	0.347	0.004	Q9VN86	4.698	0.015	Q8IN44	0.310	0.042
Q8IRG7	0.347	0.004	Q9VGQ1	8.636	0.015	Q9VZA4	0.337	0.042
Q8SZU6	0.347	0.004	Q6NLJ9	2.064	0.015	P81160	4.401	0.042
X2JAU8-4	4.117	0.004	M9NDL7	0.432	0.016	A9YK67	2.064	0.043
X2JAU8-6	4.117	0.004	Q8MQS4	0.432	0.016	A9YK77	2.064	0.043
M9PDV6	10.263	0.005	Q9W266	2.853	0.016	Q9W3B3	2.064	0.043
M9PE32	10.263	0.005	A0A1Z1CN86	2.221	0.016	Q8SWS2	3.229	0.043
Q9VZY3	10.263	0.005	Q9VHK7	2.221	0.016	Q9VYM7	3.229	0.043
P51592	9.412	0.005	E1JIE5	0.463	0.016	X2JEY3	3.229	0.043
Q9VPC2	2.286	0.005	Q9VS49	0.463	0.016	Q6NL82	0.483	0.046
Q6NND1	3.627	0.005	Q9VC23	5.582	0.016	Q8IMT8	0.483	0.046
Q8IMC1	3.627	0.005	B0LUZ3	2.123	0.017	Q9VBY7	0.483	0.046
Q9V4E7	3.627	0.005	Q2UYJ4	2.123	0.017	M9NEV7	3.590	0.047
Q7JYY0	0.203	0.006	Q2UYM0	2.123	0.017	Q9VII1	3.590	0.047
Q9VDH8	0.203	0.006	Q2UYN1	2.123	0.017	P47947	0.290	0.049
						Q9VIU8	3.218	0.050

§UniProt; ¶, Cutoff ±2; 3, p-value generated by Student's t-test.

Table S8. Details of *Drosophila melanogaster* differentially regulated proteins used in the Sanky's plot

UniProt Accession No	Gene	Protein
Muscle		
Q9VU84	Abp1	Drebrin-like protein (Actin binding protein 1)
M9MRD1	Msp300	Muscle-specific protein
P48603	cpb	F-actin-capping protein subunit beta
Q03427	LamC	Lamin-C
P19351-13	up	Isoform 13 of Troponin T
P22979	Hsp67Bc	Heat shock protein 67B3
M9MRX0	Lmpt	Limpet, isoform J
Q58CJ5	sals	GH01093p
Q99323	zip	Myosin heavy chain, non-muscle
P36188-3	wupA	Isoform 2 of Troponin I
P12080	if	Integrin alpha-PS2
A0A0B4LFW8	tn	Thin, isoform E
Q9VCA8	mask	Ankyrin repeat and KH domain-containing protein mask
P05661-25	Mhc	Isoform L of Myosin heavy chain, muscle
P49455	Tm1	Tropomyosin-1, isoforms 33/34
Q8SX68	Naus	CTTNBP2 N-terminal-like protein
X2JAU8-4	nwk	Isoform 4 of Protein nervous wreck
P19351-12	up	Isoform 12 of Troponin T, skeletal muscle
P36188-9	wupA	Isoform 8 of Troponin I
P09491	Tm2	Tropomyosin-2
O01367	how	Protein held out wings
A1ZA47-4	Zasp52	Isoform E of PDZ and LIM domain protein Zasp
Q9VMT2	TpnC25D	GEO07854p1
M9PCE0	Pgk	Phosphoglycerate kinase
Neurons		
Q9VLP1	Dmel\CG14275	Protein quiver, drome
Q9VLP2	Dmel\CG7781	Protein quiver, drome
Q9VIH9	BcDNA:GH07967	Protein quiver, drome
Q9VII5	Dmel\CG31676	Protein quiver
B5RJE5	CG31030-RA	FI09225p
Q6NL82	veli	Protein lin-7 homolog
Q8IPM8	cpx	Complexin
M9PDV6	Fife	Fife, isoform B
P25445[a]	FAS	Tumor necrosis factor receptor superfamily member 6, human
Q9VU84	Abp1	Drebrin-like protein (Actin binding protein 1)

P91621	sif	Protein still life, isoform SIF type 1
O96967	Crys	Drosocrystallin
P06002	ninaE	Opsin Rh1
Q09103-3	rdgA	Eye-specific diacylglycerol kinase (Retinal degeneration A protein)
M9NEV7	BcDNA:RE71975	Protein quiver
X2JAU8-4	nwk	Protein nervous wreck
A0A0B4KEW6	Amph	Amphiphysin, isoform B
P10676	ninaC	Neither inactivation nor afterpotential protein C
P62152	Cam	Calmodulin
P91927	Letm1	Mitochondrial proton/calcium exchanger protein
Metabolism		
Q7JYY0	RpS30	RH08962p (Ribosomal protein)
Q9VRL0	Cyt-c1	Cytochrome c1, isoform A
P07836[b]	-	Actin, muscle-type A1
Q9W334	RpS28b	Small ribosomal subunit protein eS28
Q02748	eIF4A	Eukaryotic initiation factor 4A
P55841	RpL14	Large ribosomal subunit protein eL14
Q9VTZ6	Pmm2	Phosphomannomutase
P05389	RpLP2	Large ribosomal subunit protein P2
A1A6X4	RpL23a	IP17216p
Q04499	slgA	Proline dehydrogenase 1, mitochondrial
O01367	how	Protein held out wings
Q86NN8	COX5B	COX5B
P39018	RpS19a	Small ribosomal subunit protein eS19A
Q9VW26	Oat	Ornithine aminotransferase, mitochondrial
Q9W2X6	ATPsyndelta	ATP synthase, delta subunit, isoform A
Q917S8[c]	-	Genome polyprotein
Q9V9M7	RpL21	60S ribosomal protein L21

[a]*Homo sapiens*; [b]*Bombyx mori*; [c]*Hepacivirus* C

(a)

16S

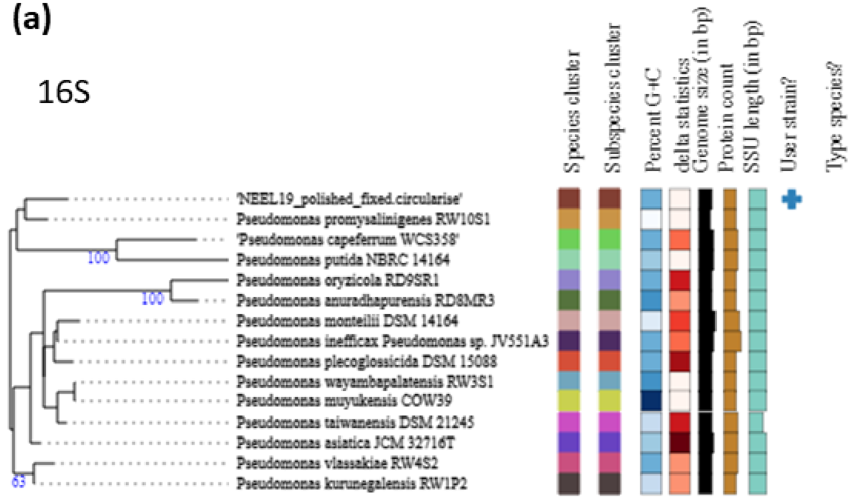

(b)

Genome

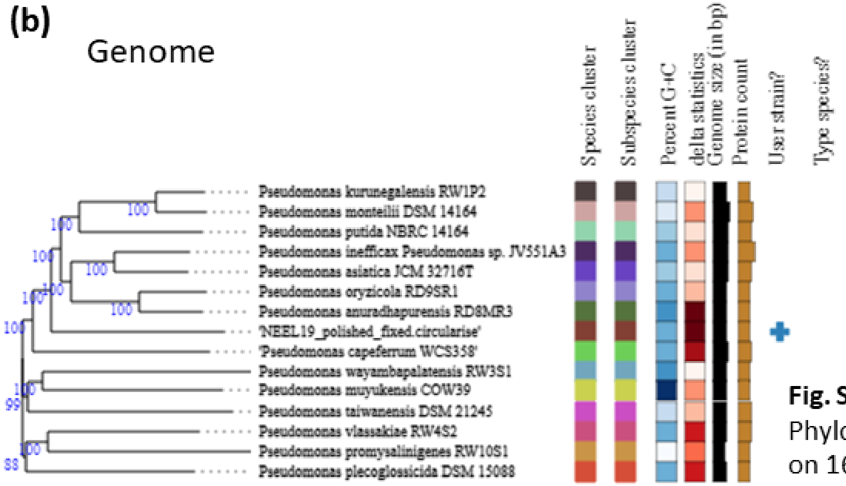

Fig. S1. Phylogenetic analysis of NEEL19 at TYGS. Phylogenetic trees showing the position of NEEL19 based on 16S rRNA gne (a) and genome (b) through default setting are shown.

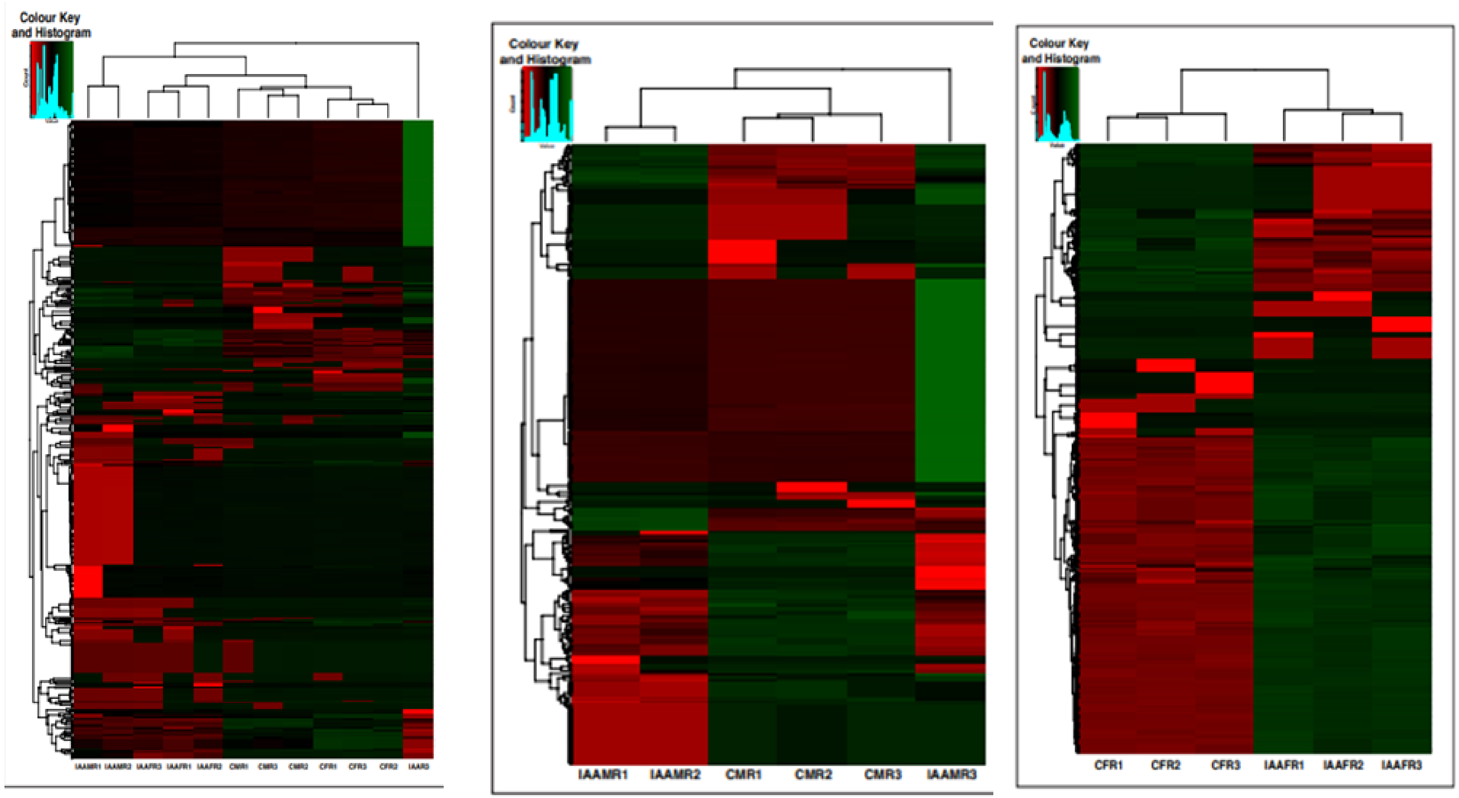

Fig. S2. Heatmap showing differentially regulated proteins of wild-type fruit fly (*Drosophila melanogaster*) head as a function of exposure to bacterial IAA. Differentially regulated head protein with and without IAA treatment in flies (a), with and without IAA treatment in male flies (b) and with and without IAA treatment in female flies (c). Dark green, upregulation; dark red, down regulation.

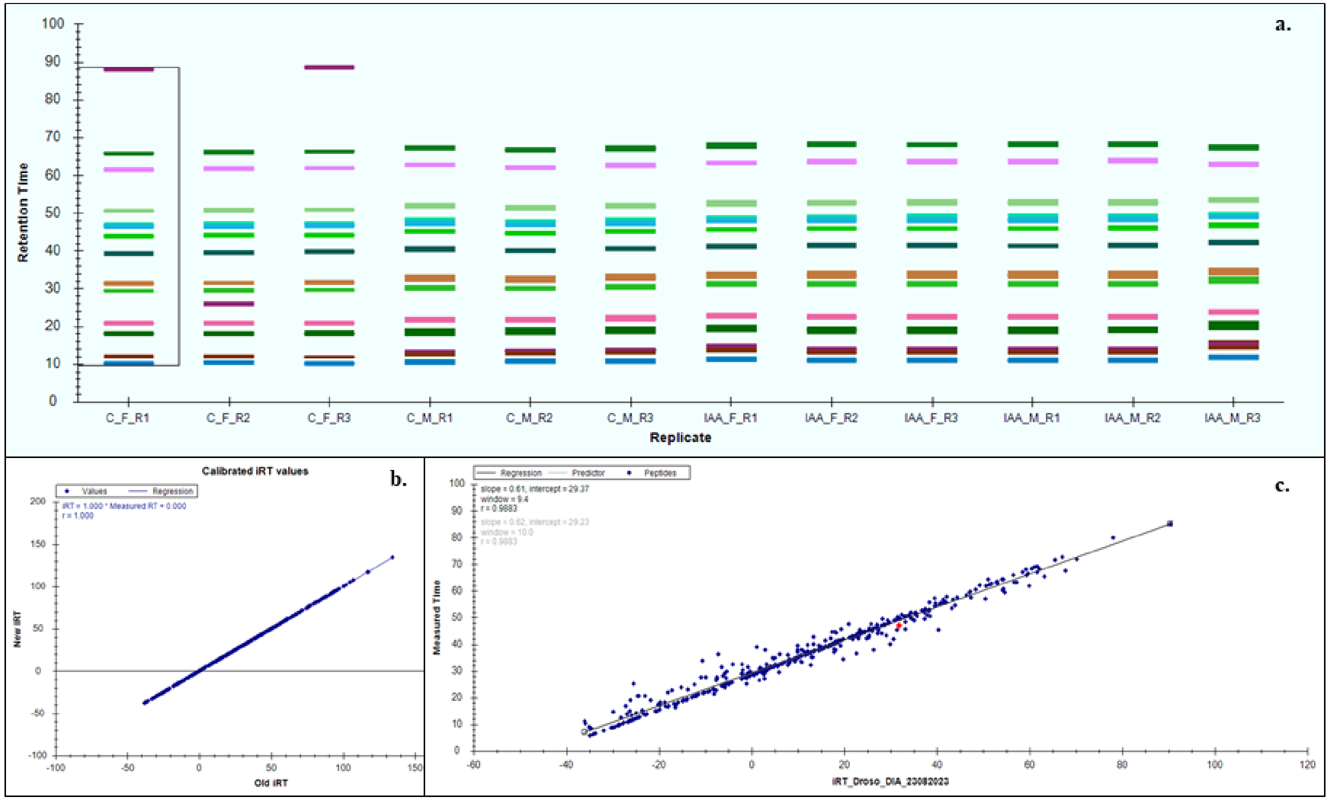

Fig. S4: Retention time calibration and distribution of DIA data as compared to ESL. a) Retention time of internal standard (14 Thermo pierce iRT peptides) in DIA data. X-axis indicates retention time in minutes and y-axis indicates the file/sample name. b) Calibrated iRT values containing peptides from internal standard and peptides from GPF data. This model was used for retention time correction in DIA data. c) Distribution of measured retention time in the DIA data as compared to calibrated iRT model.

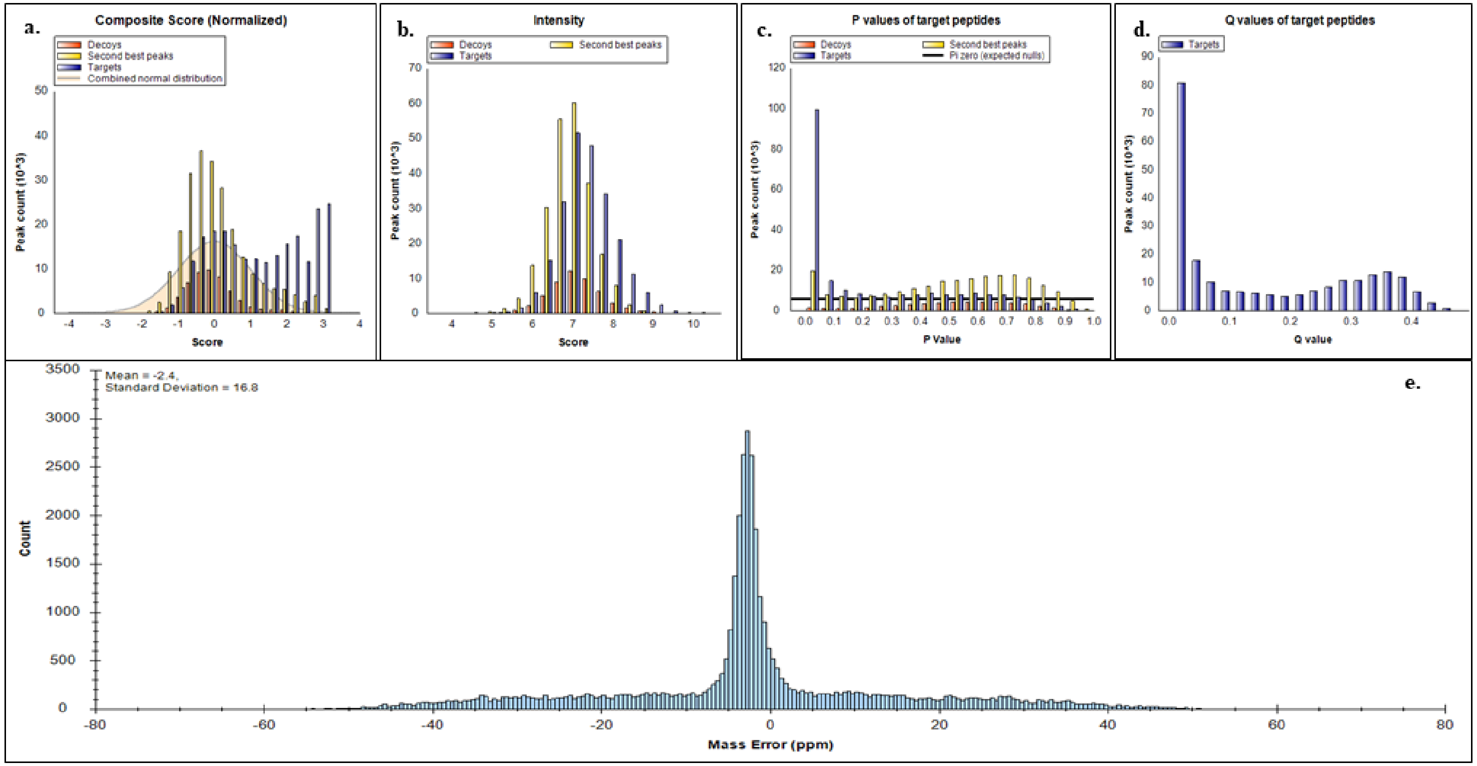

Fig. S5: Target-decoy assessment model (mProphet) and mass error distribution of DIA data as compared to ESL. a) Normalized composite score generated by mProphet by comparing the decoys and target distribution in DIA data. b) Intensity plot of target-decoys plotted against the composite score. c) P-value distribution of target peptides showing highest peptide intensity at a p-value <0.05. d) Q-value distribution of target peptides showing highest intensity at a q-value <0.05. e) Mass error distribution of all the identified peptides plotted as count vs mass error in ppm.
