# Supplementary material for: Comprehensive insights into the impact of bacterial indole-3-acetic acid on sensory preferences in Drosophila melanogaster
Source: Sci Rep. 2024 Apr 9;14:8311. doi: 10.1038/s41598-024-58829-7 (PMC11003987; doi:10.1038/s41598-024-58829-7)
Supplement: Supplementary file 3 — Supplementary Information 3. [file 41598_2024_58829_MOESM3_ESM.pdf]

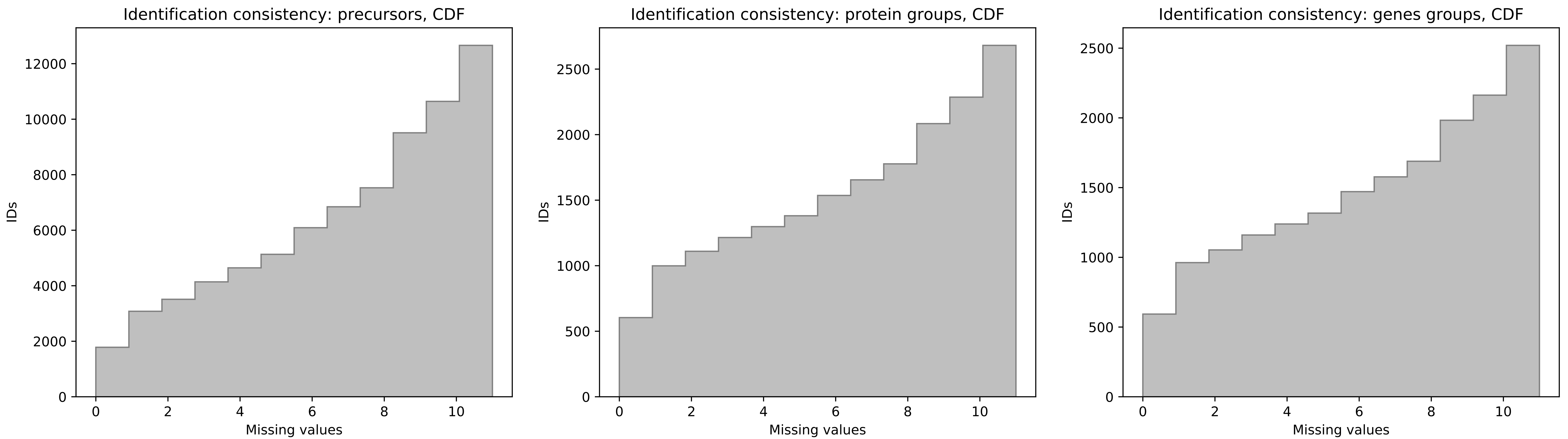
Identification consistency: precursors, CDF
IDs
Missing values
0
2000
4000
6000
8000
10000
12000
0
2
4
6
8
10
Identification consistency: protein groups, CDF
IDs
Missing values
0
500
1000
1500
2000
2500
0
2
4
6
8
10
Identification consistency: genes groups, CDF
IDs
Missing values
0
500
1000
1500
2000
2500
0
2
4
6
8
10

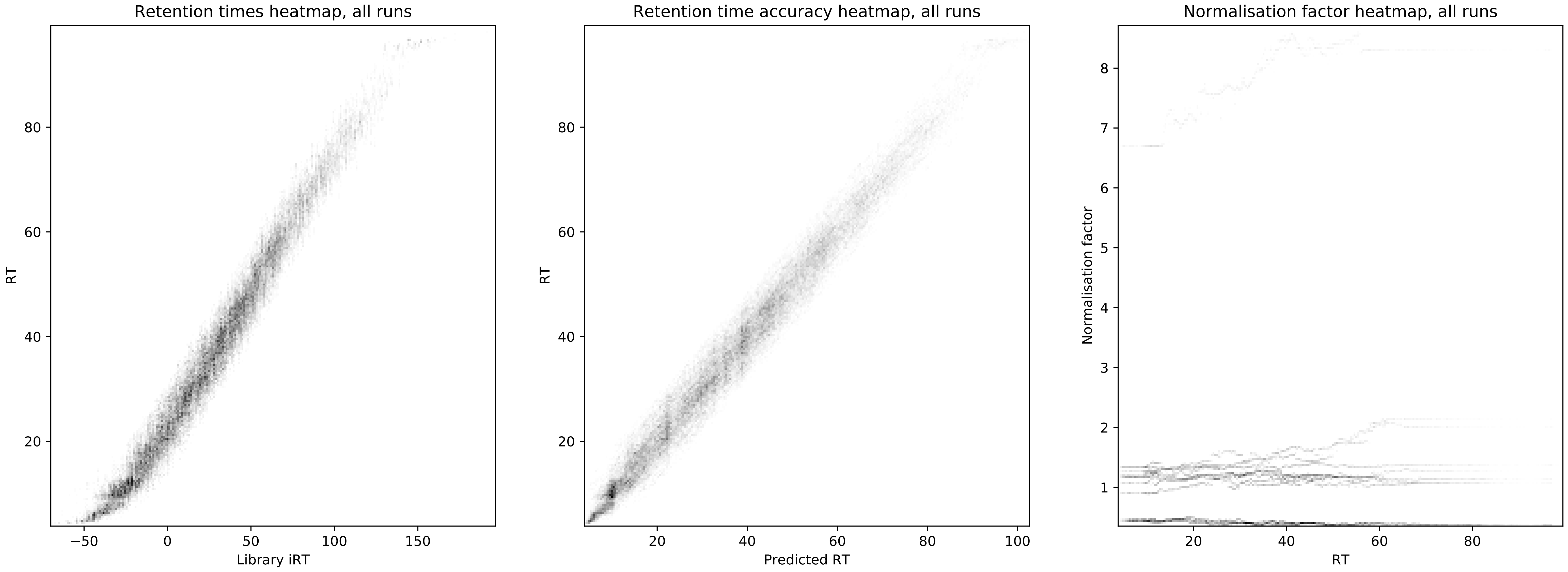

Retention times heatmap, all runs
RT
Library iRT
Retention time accuracy heatmap, all runs
RT
Predicted RT
Normalisation factor heatmap, all runs
Normalisation factor
RT

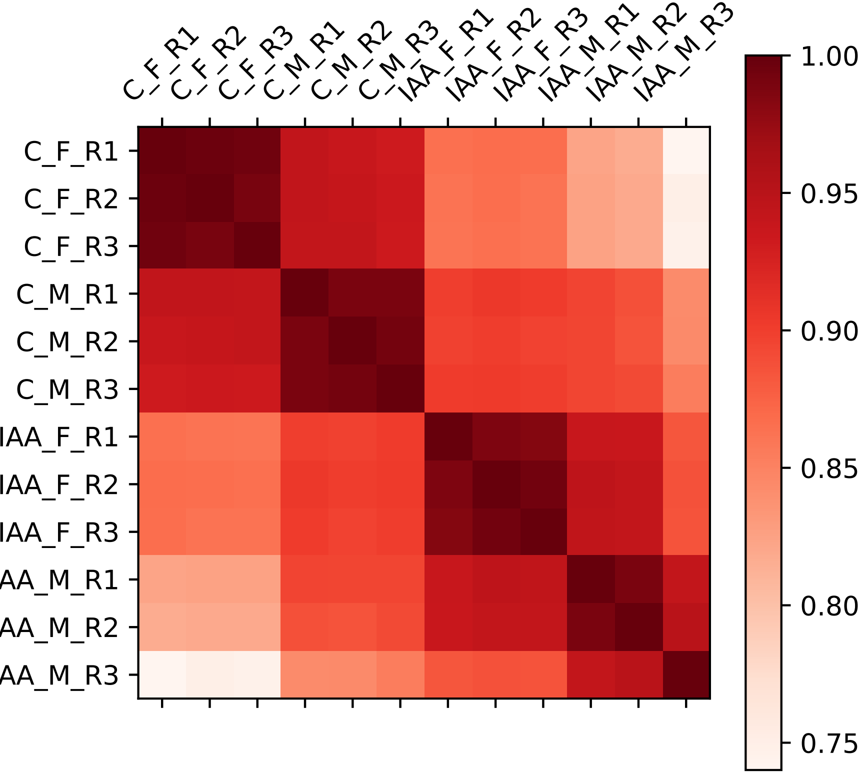

C_F_R1
C_F_R2
C_F_R3
C_M_R1
C_M_R2
C_M_R3
IAA_F_R1
IAA_F_R2
IAA_F_R3
IAA_M_R1
IAA_M_R2
IAA_M_R3
1.00
0.95
0.90
0.85
0.80
0.75

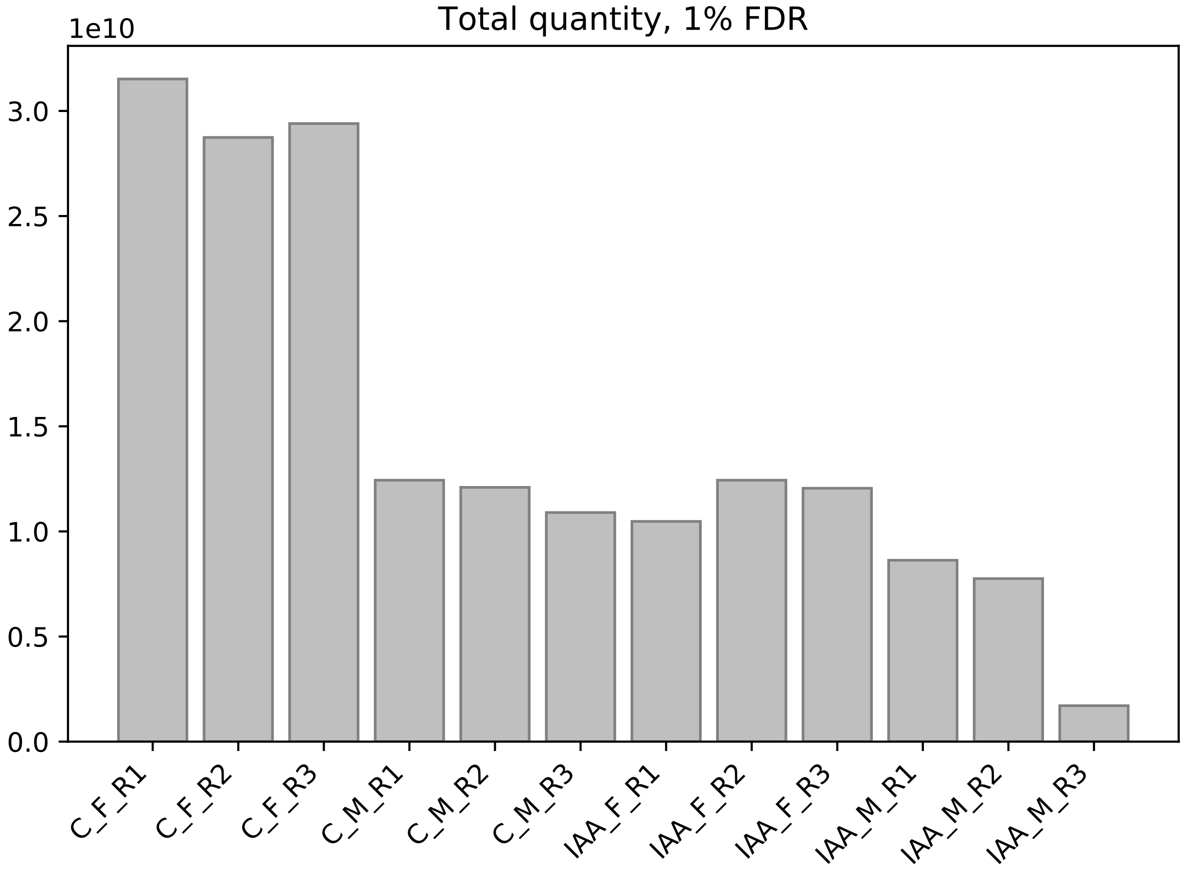

Total quantity, 1% FDR
1e10
3.0
2.5
2.0
1.5
1.0
0.5
0.0
C_F_R1
C_F_R2
C_F_R3
C_M_R1
C_M_R2
C_M_R3
IAA_F_R1
IAA_F_R2
IAA_F_R3
IAA_M_R1
IAA_M_R2
IAA_M_R3

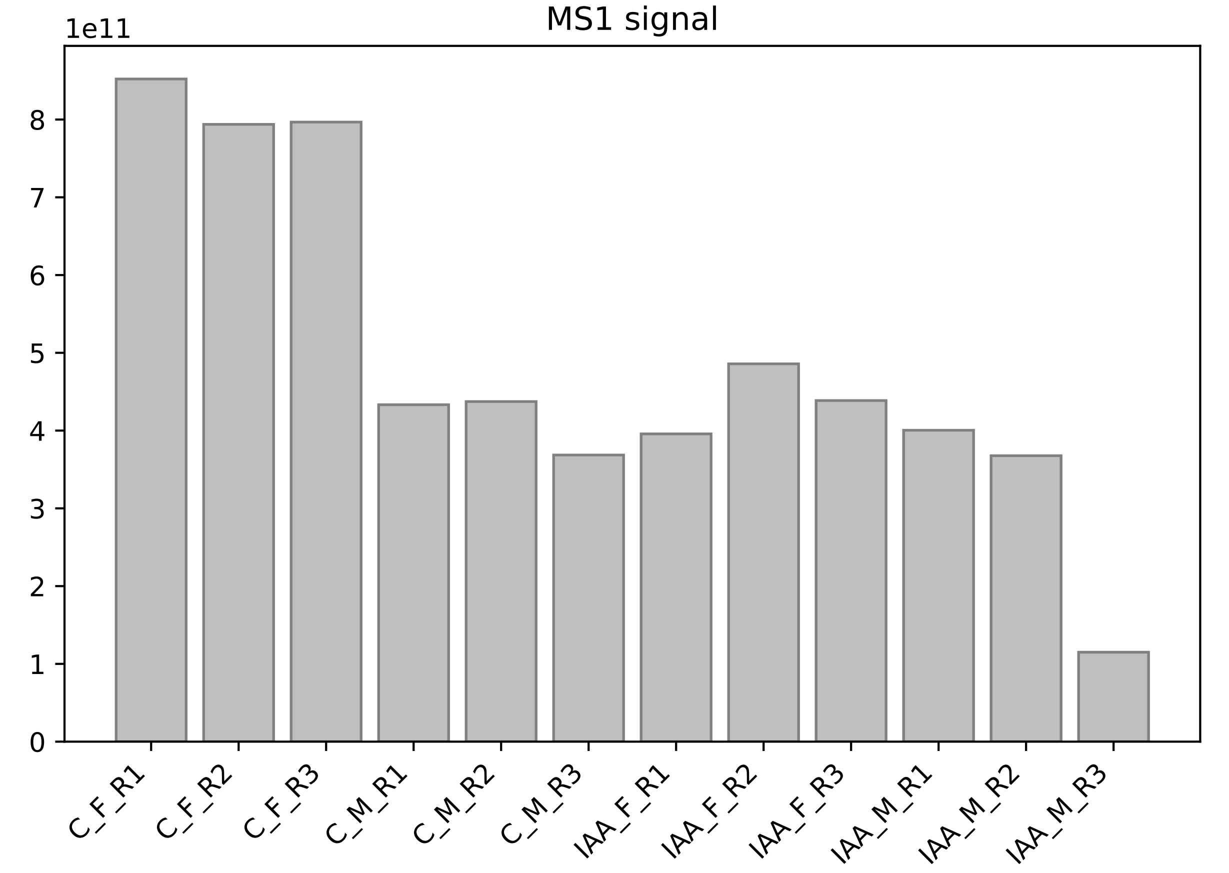

MS1 signal
1e11
0
1
2
3
4
5
6
7
8
C_F_R1
C_F_R2
C_F_R3
C_M_R1
C_M_R2
C_M_R3
IAA_F_R1
IAA_F_R2
IAA_F_R3
IAA_M_R1
IAA_M_R2
IAA_M_R3

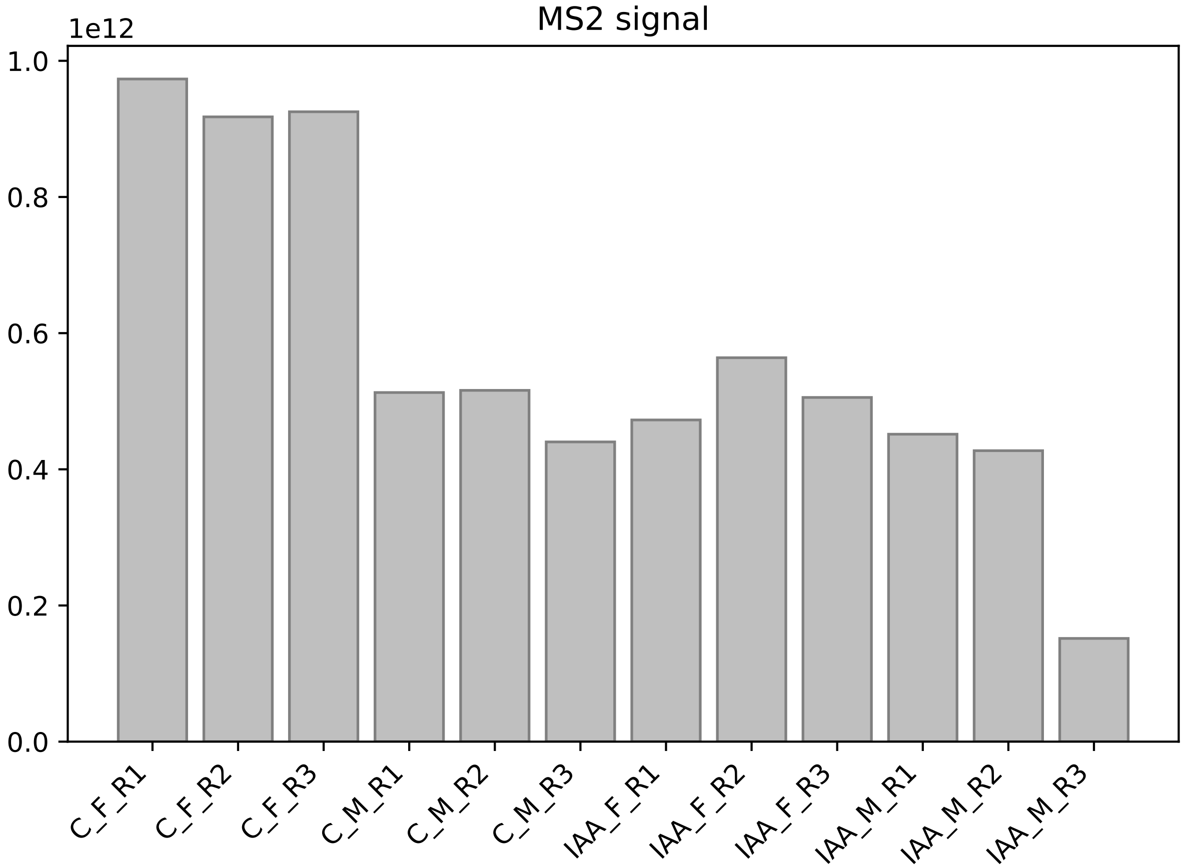

MS2 signal
1e12
1.0
0.8
0.6
0.4
0.2
0.0
C_F_R1
C_F_R2
C_F_R3
C_M_R1
C_M_R2
C_M_R3
IAA_F_R1
IAA_F_R2
IAA_F_R3
IAA_M_R1
IAA_M_R2
IAA_M_R3

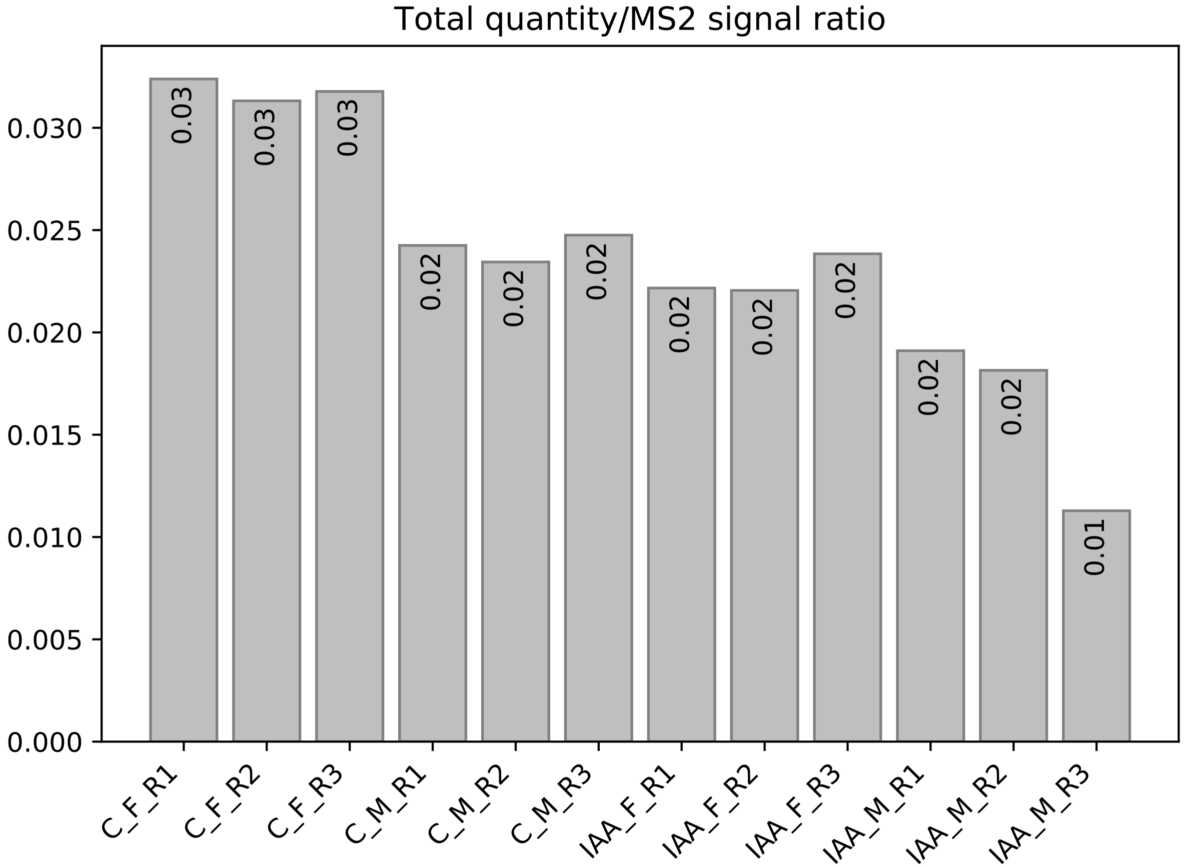

Total quantity/MS2 signal ratio
0.030
0.025
0.020
0.015
0.010
0.005
0.000
0.03
0.03
0.03
0.02
0.02
0.02
0.02
0.02
0.02
0.02
0.02
0.01
C_F_R1
C_F_R2
C_F_R3
C_M_R1
C_M_R2
C_M_R3
IAA_F_R1
IAA_F_R2
IAA_F_R3
IAA_M_R1
IAA_M_R2
IAA_M_R3

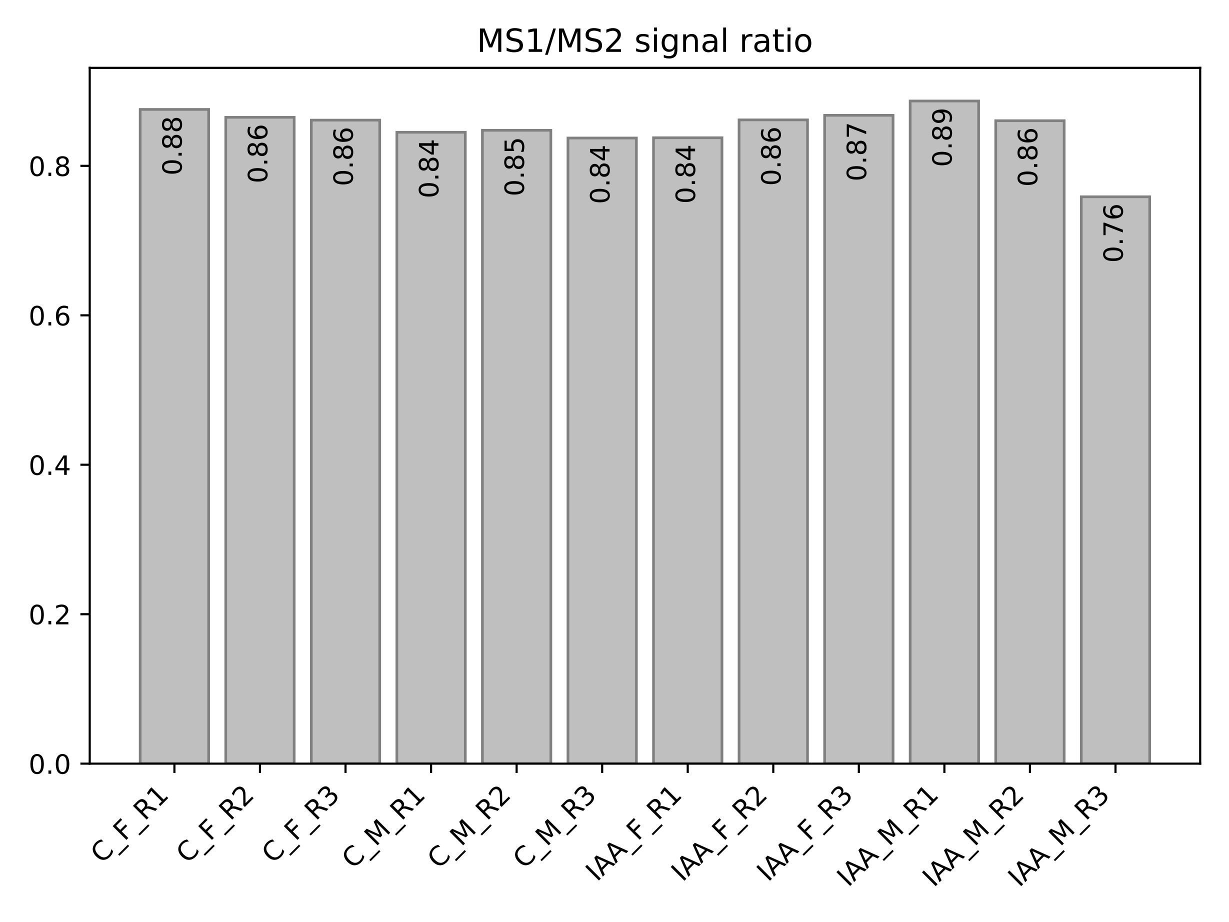

MS1/MS2 signal ratio
0.0
0.2
0.4
0.6
0.8
0.88
0.86
0.86
0.84
0.85
0.84
0.84
0.86
0.87
0.89
0.86
0.76
C_F_R1
C_F_R2
C_F_R3
C_M_R1
C_M_R2
C_M_R3
IAA_F_R1
IAA_F_R2
IAA_F_R3
IAA_M_R1
IAA_M_R2
IAA_M_R3

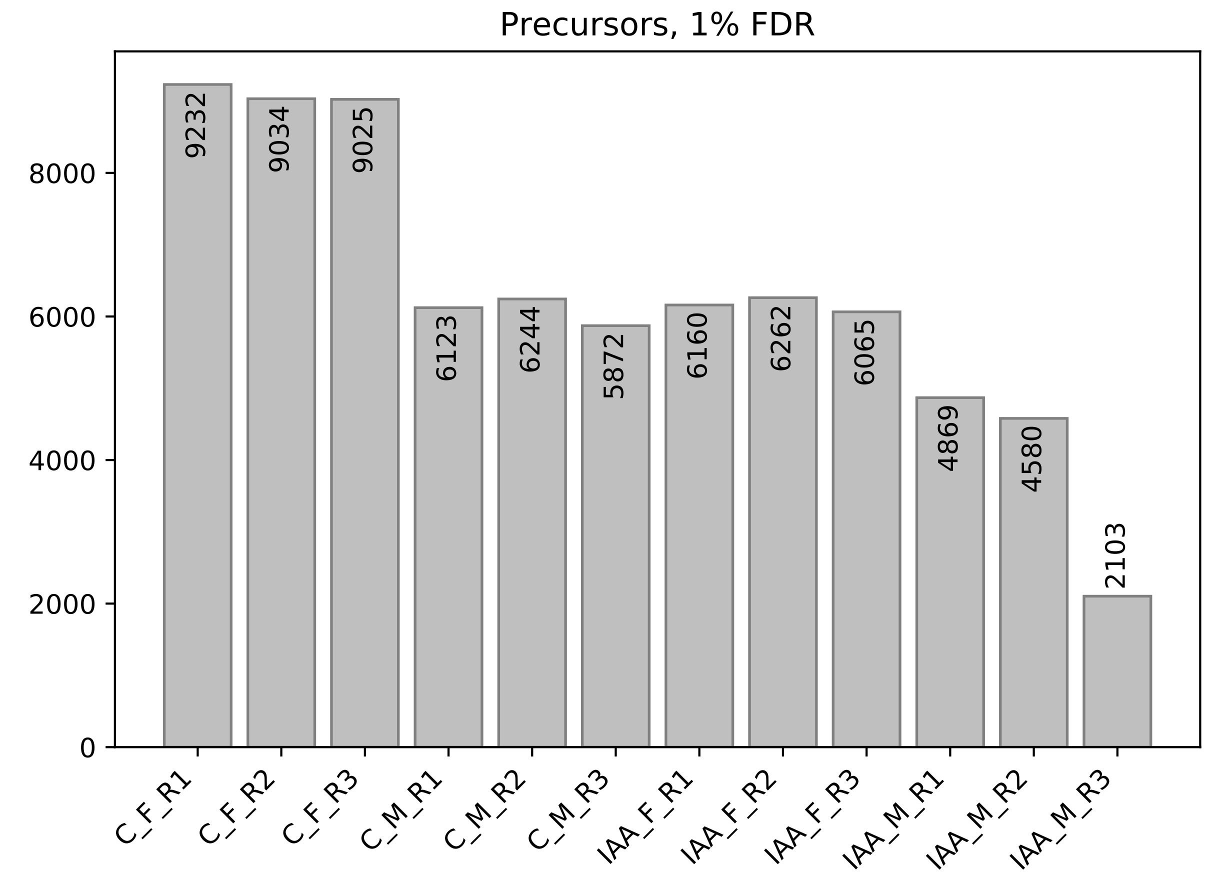
Precursors, 1% FDR
0
2000
4000
6000
8000
9232
9034
9025
6123
6244
5872
6160
6262
6065
4869
4580
2103
C_F_R1
C_F_R2
C_F_R3
C_M_R1
C_M_R2
C_M_R3
IAA_F_R1
IAA_F_R2
IAA_F_R3
IAA_M_R1
IAA_M_R2
IAA_M_R3

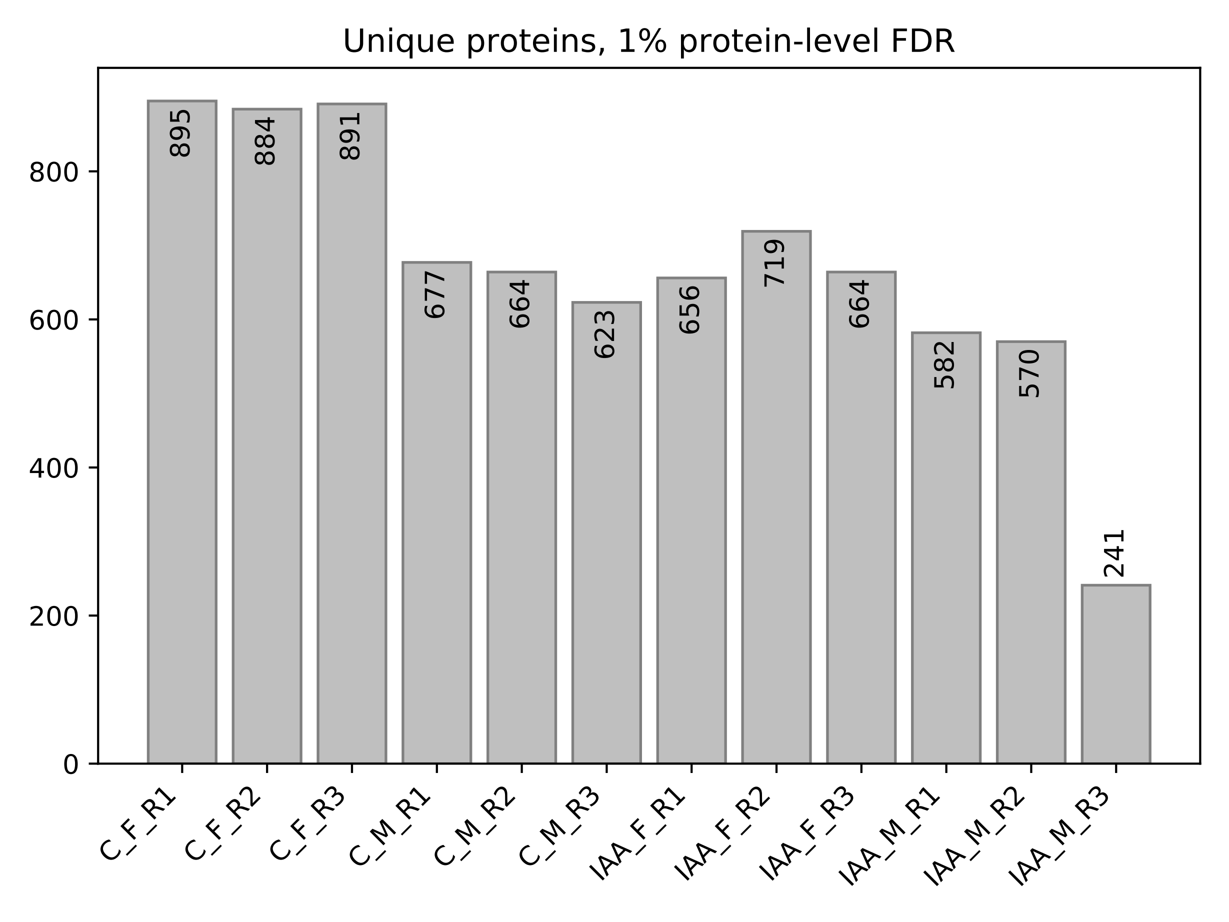
Unique proteins, 1% protein-level FDR
0
200
400
600
800
895
884
891
677
664
623
656
719
664
582
570
241
C_F_R1
C_F_R2
C_F_R3
C_M_R1
C_M_R2
C_M_R3
IAA_F_R1
IAA_F_R2
IAA_F_R3
IAA_M_R1
IAA_M_R2
IAA_M_R3

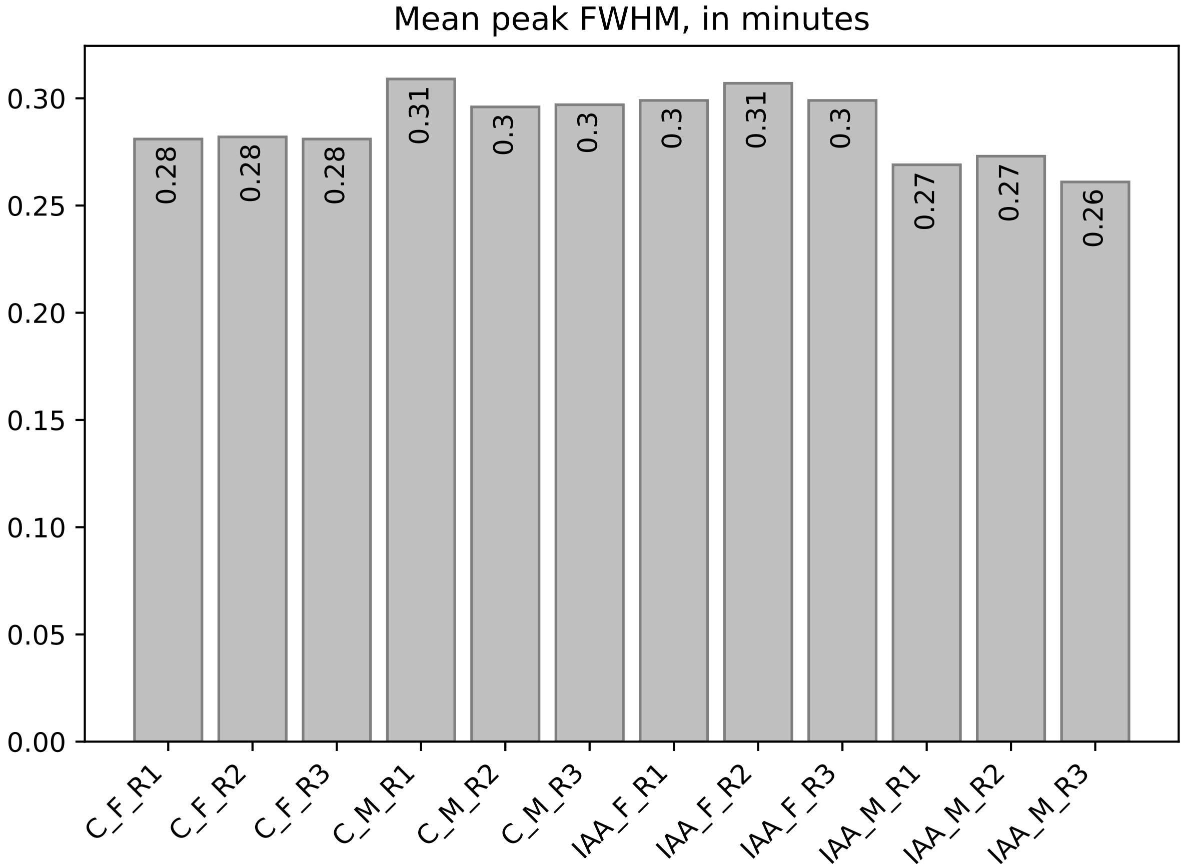

Mean peak FWHM, in minutes
0.30
0.25
0.20
0.15
0.10
0.05
0.00
0.28
0.28
0.28
0.31
0.3
0.3
0.3
0.31
0.3
0.27
0.27
0.26
C_F_R1
C_F_R2
C_F_R3
C_M_R1
C_M_R2
C_M_R3
IAA_F_R1
IAA_F_R2
IAA_F_R3
IAA_M_R1
IAA_M_R2
IAA_M_R3

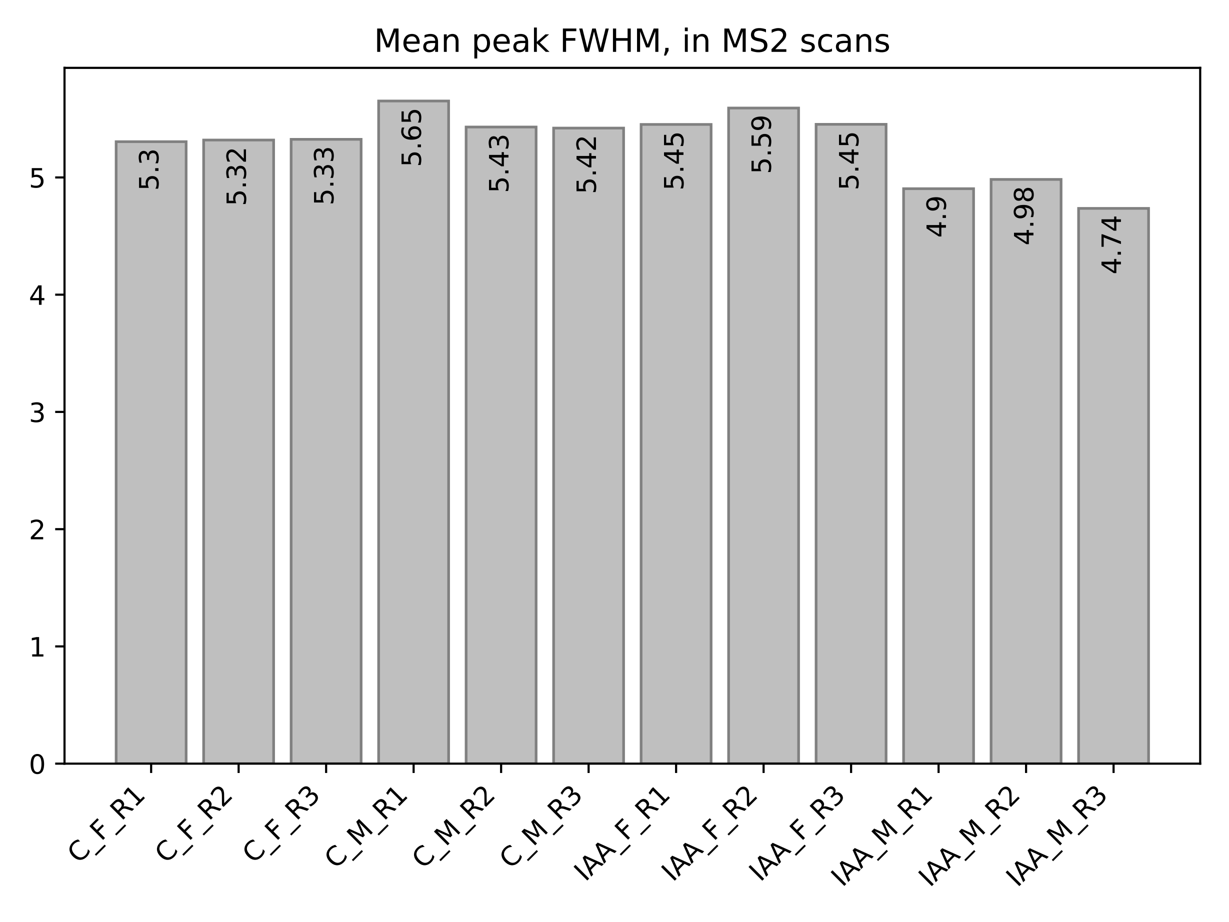

Mean peak FWHM, in MS2 scans
0
1
2
3
4
5
5.3
5.32
5.33
5.65
5.43
5.42
5.45
5.59
5.45
4.9
4.98
4.74
C_F_R1
C_F_R2
C_F_R3
C_M_R1
C_M_R2
C_M_R3
IAA_F_R1
IAA_F_R2
IAA_F_R3
IAA_M_R1
IAA_M_R2
IAA_M_R3

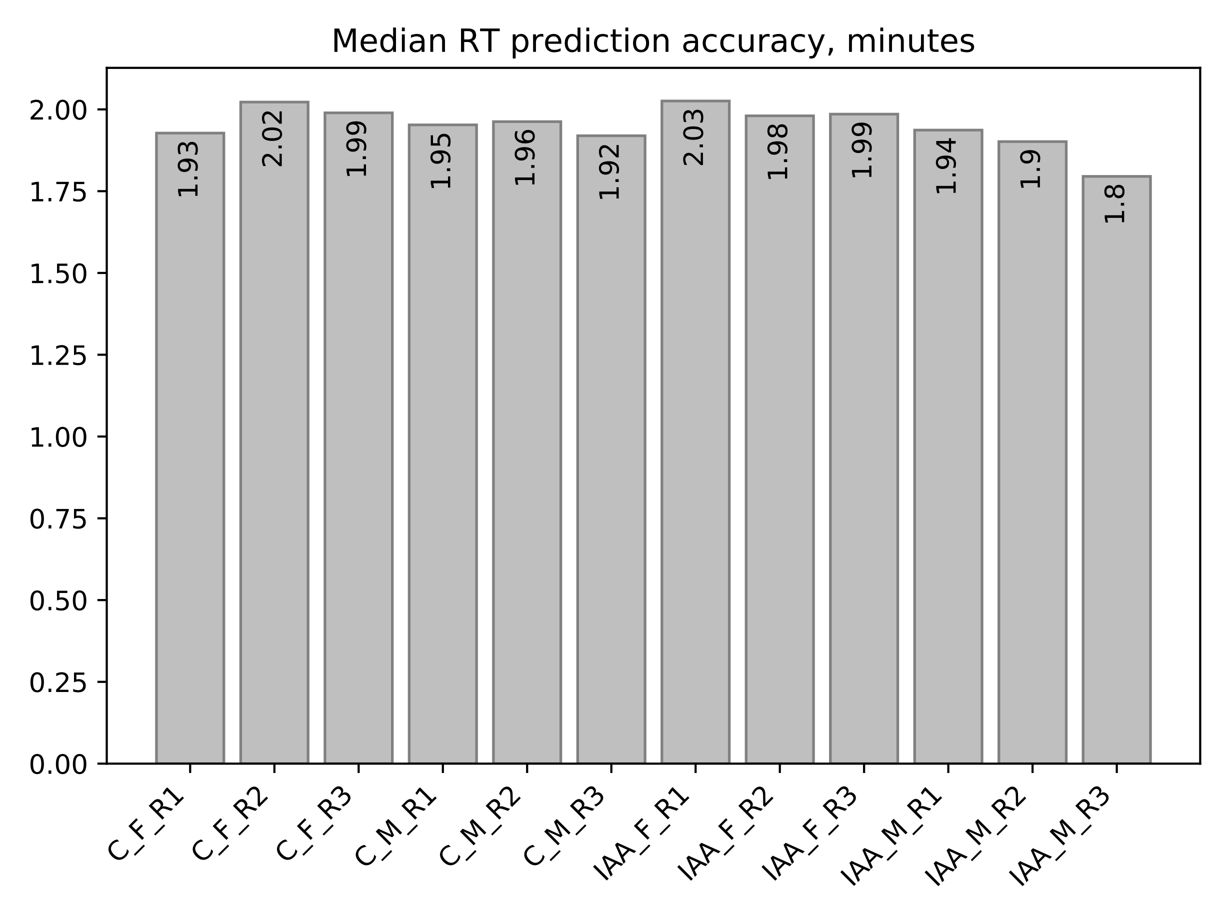
Median RT prediction accuracy, minutes
0.00
0.25
0.50
0.75
1.00
1.25
1.50
1.75
2.00
1.93
2.02
1.99
1.95
1.96
1.92
2.03
1.98
1.99
1.94
1.9
1.8
C_F_R1
C_F_R2
C_F_R3
C_M_R1
C_M_R2
C_M_R3
IAA_F_R1
IAA_F_R2
IAA_F_R3
IAA_M_R1
IAA_M_R2
IAA_M_R3

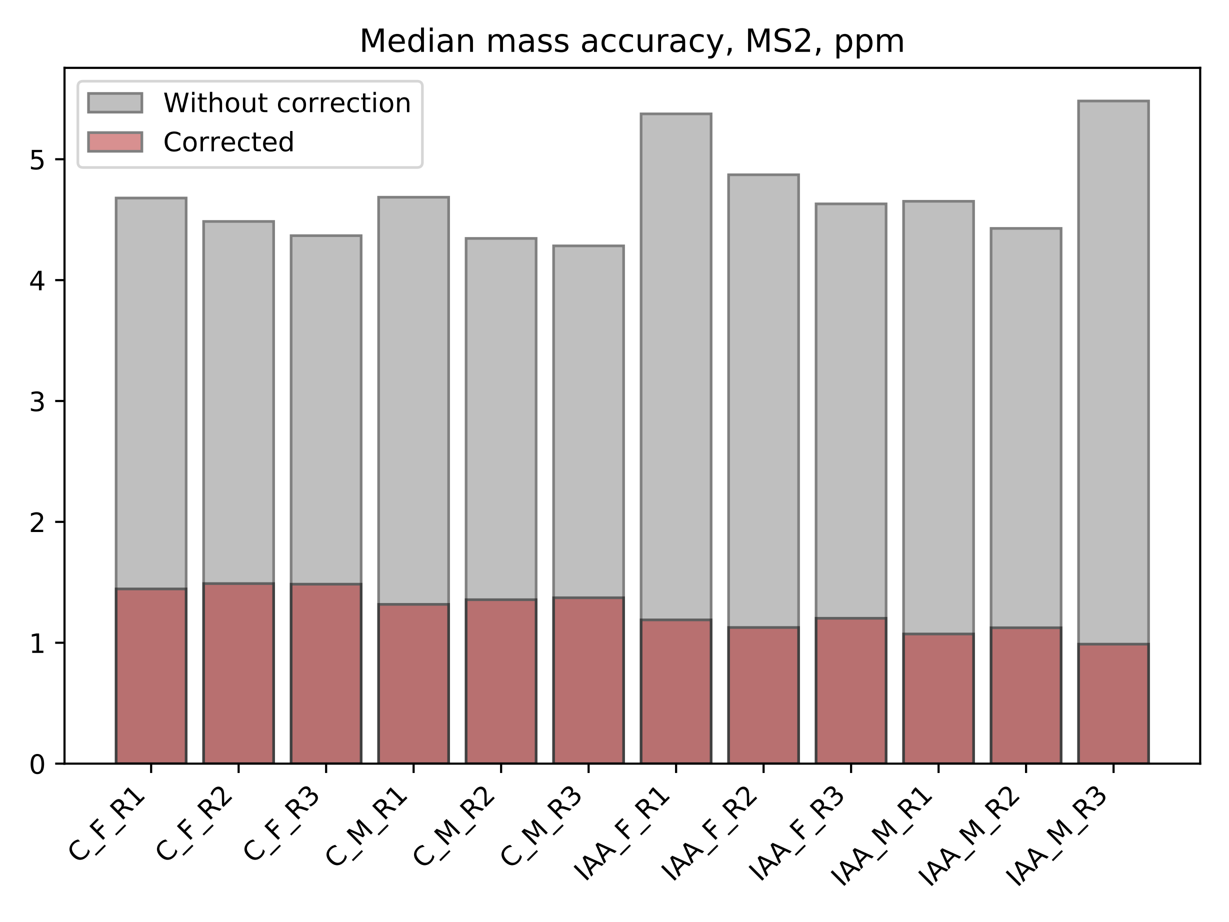

Median mass accuracy, MS2, ppm
Without correction
Corrected
0
1
2
3
4
5
C_F_R1
C_F_R2
C_F_R3
C_M_R1
C_M_R2
C_M_R3
IAA_F_R1
IAA_F_R2
IAA_F_R3
IAA_M_R1
IAA_M_R2
IAA_M_R3

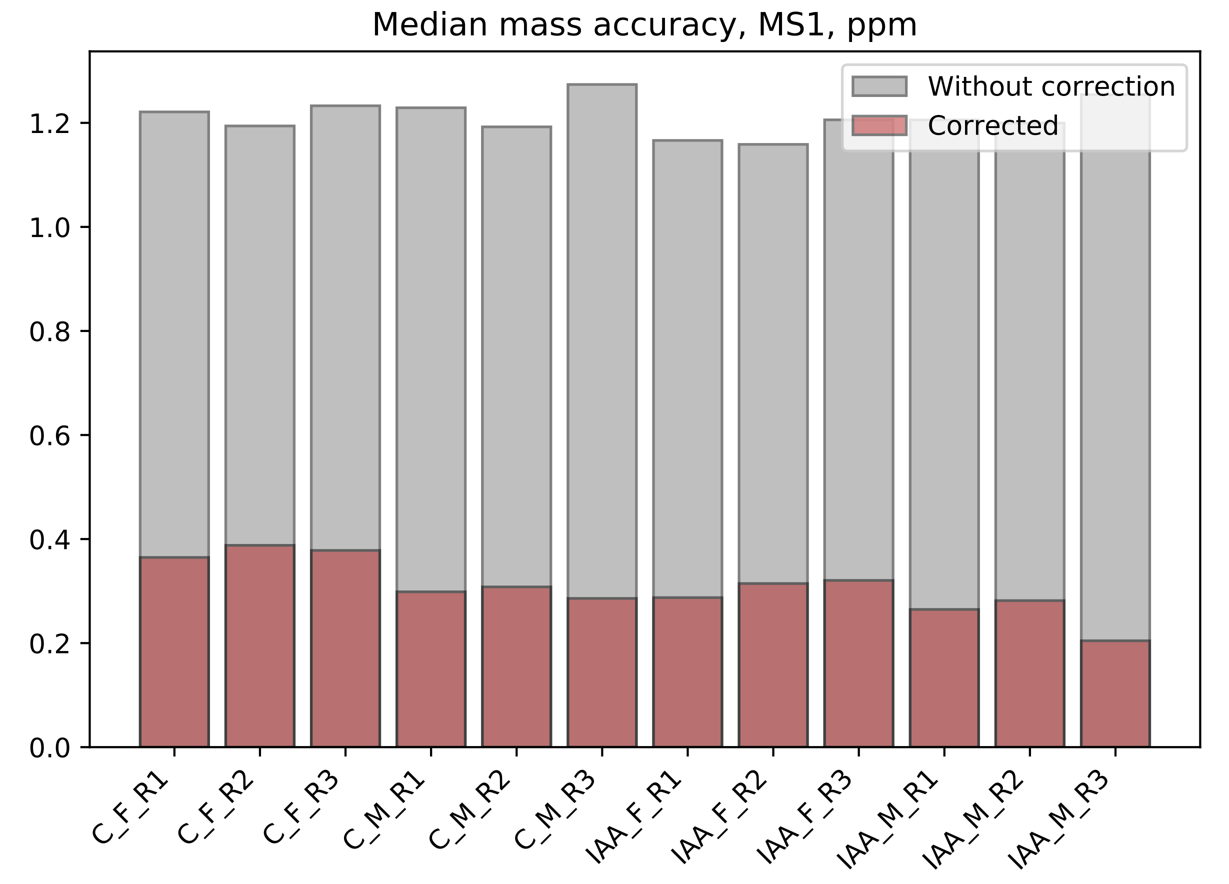
Median mass accuracy, MS1, ppm
Without correction
Corrected
0.0
0.2
0.4
0.6
0.8
1.0
1.2
C_F_R1
C_F_R2
C_F_R3
C_M_R1
C_M_R2
C_M_R3
IAA_F_R1
IAA_F_R2
IAA_F_R3
IAA_M_R1
IAA_M_R2
IAA_M_R3

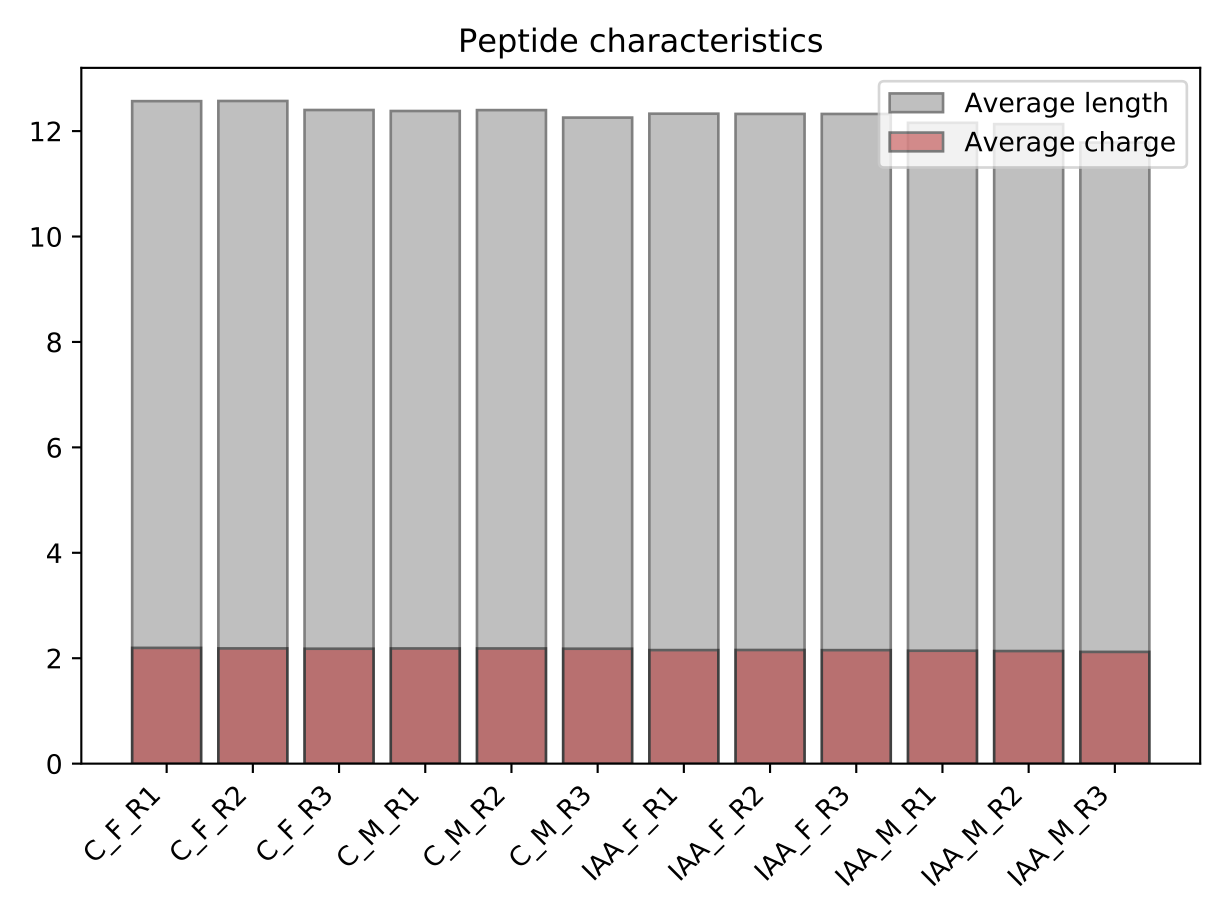
Peptide characteristics
Average length
Average charge
0
2
4
6
8
10
12
C_F_R1
C_F_R2
C_F_R3
C_M_R1
C_M_R2
C_M_R3
IAA_F_R1
IAA_F_R2
IAA_F_R3
IAA_M_R1
IAA_M_R2
IAA_M_R3

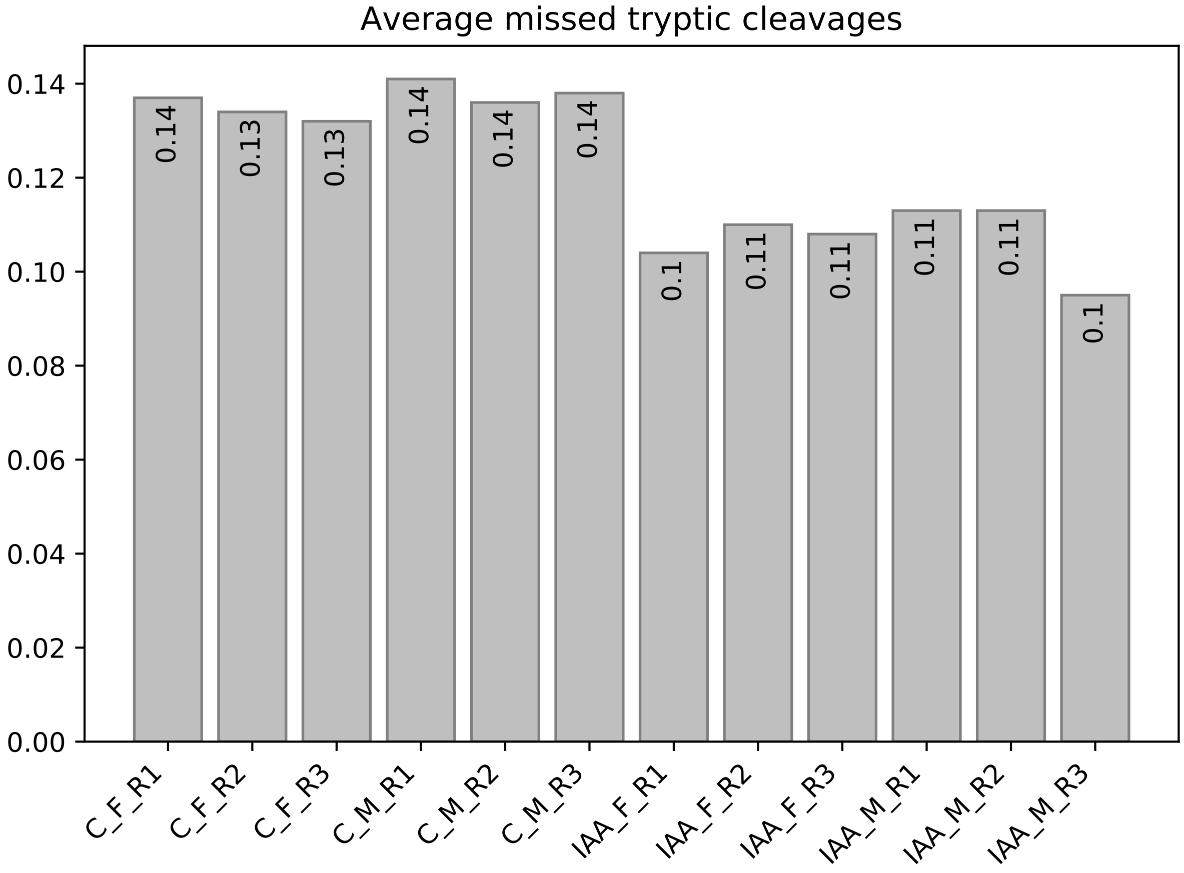
Average missed tryptic cleavages
0.14
0.12
0.10
0.08
0.06
0.04
0.02
0.00
0.14
0.13
0.13
0.14
0.14
0.14
0.1
0.11
0.11
0.11
0.11
0.1
C_F_R1
C_F_R2
C_F_R3
C_M_R1
C_M_R2
C_M_R3
IAA_F_R1
IAA_F_R2
IAA_F_R3
IAA_M_R1
IAA_M_R2
IAA_M_R3

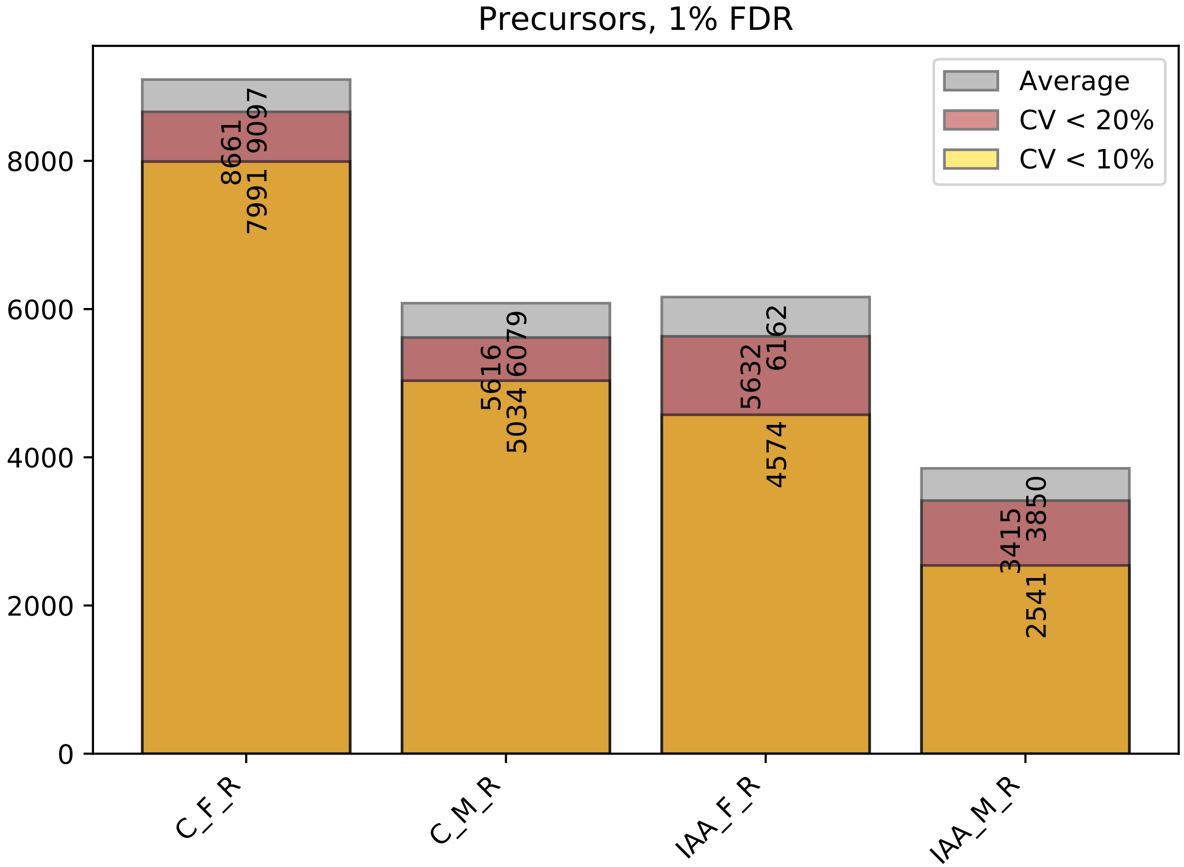

Precursors, 1% FDR
Average
CV < 20%
CV < 10%
0
2000
4000
6000
8000
9097
8661
7991
6079
5616
5034
6162
5632
4574
3850
3415
2541
C_F_R
C_M_R
IAA_F_R
IAA_M_R

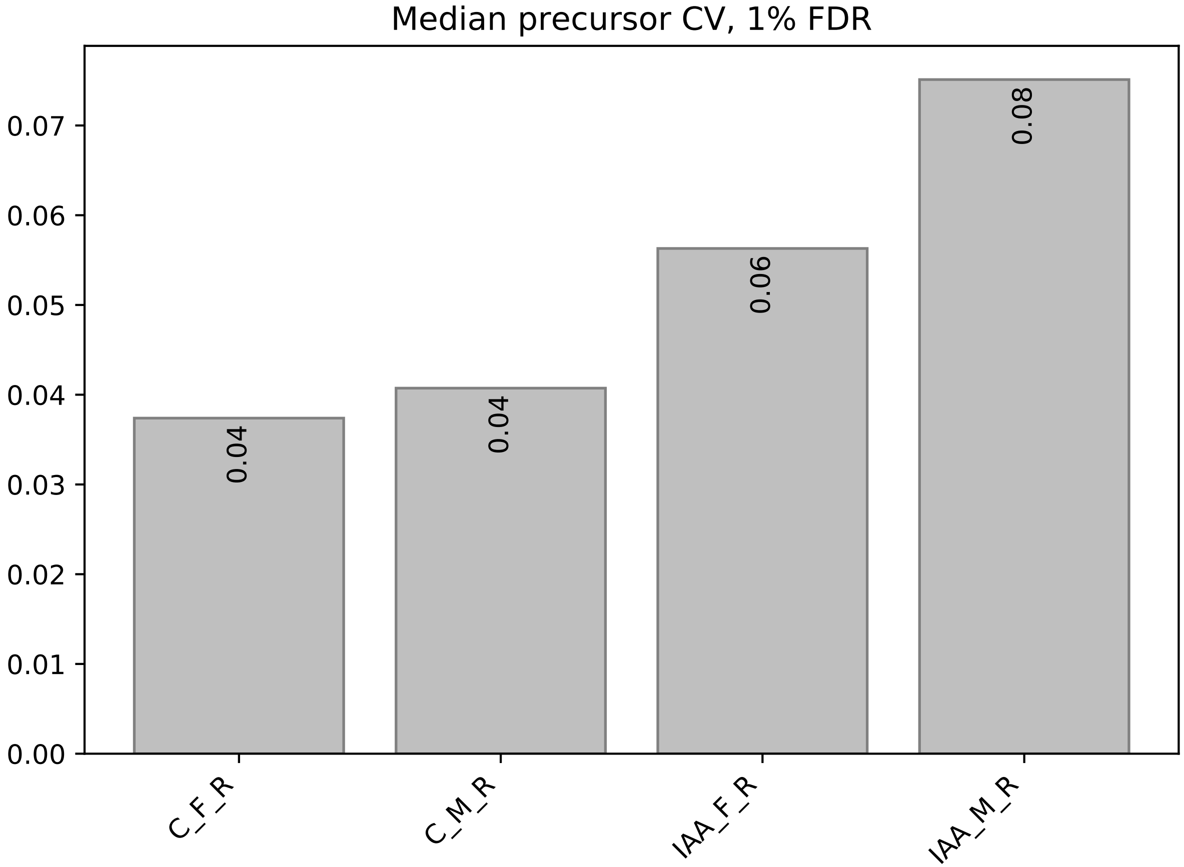

Median precursor CV, 1% FDR
0.00
0.01
0.02
0.03
0.04
0.05
0.06
0.07
0.04
0.04
0.06
0.08
C_F_R
C_M_R
IAA_F_R
IAA_M_R

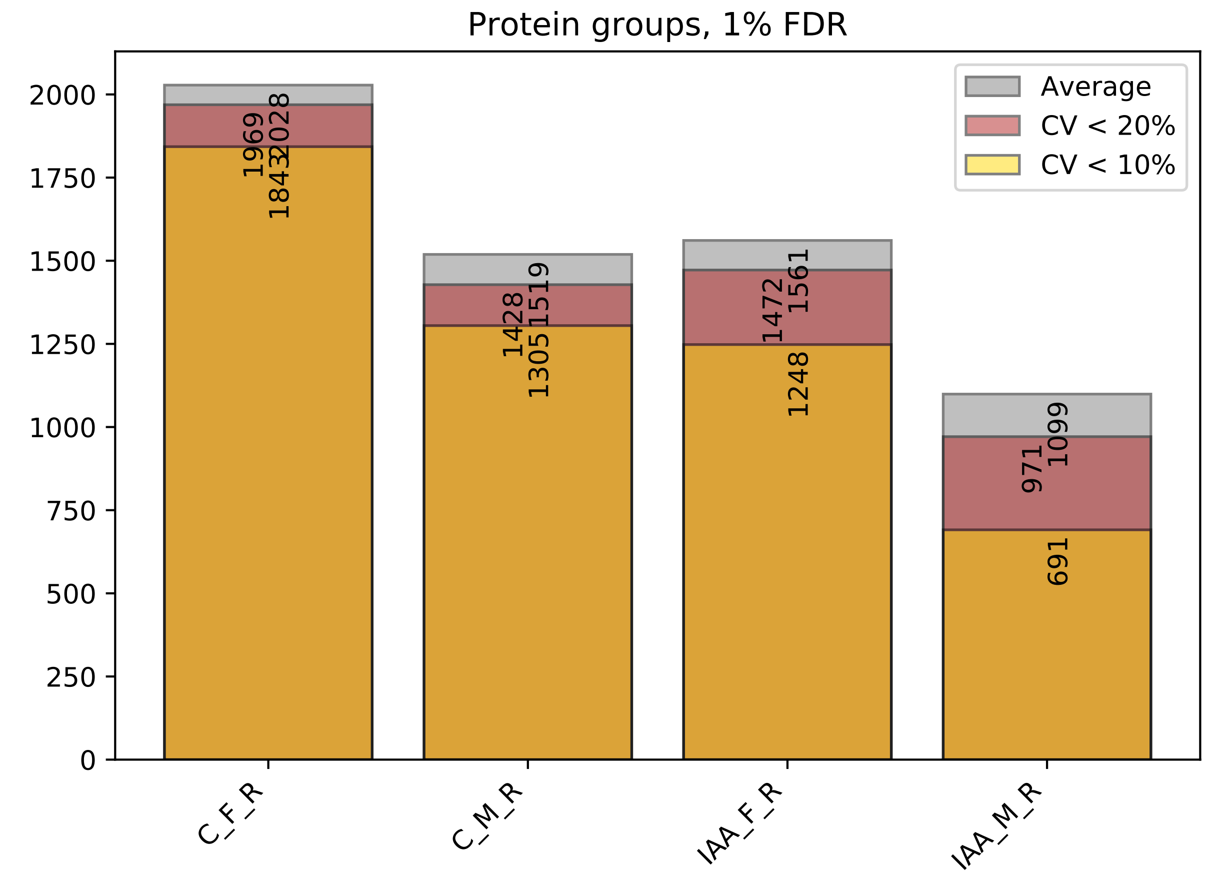

Protein groups, 1% FDR
Average
CV < 20%
CV < 10%
0
250
500
750
1000
1250
1500
1750
2000
2028
1969
1843
1519
1428
1305
1561
1472
1248
1099
971
691
C_F_R
C_M_R
IAA_F_R
IAA_M_R

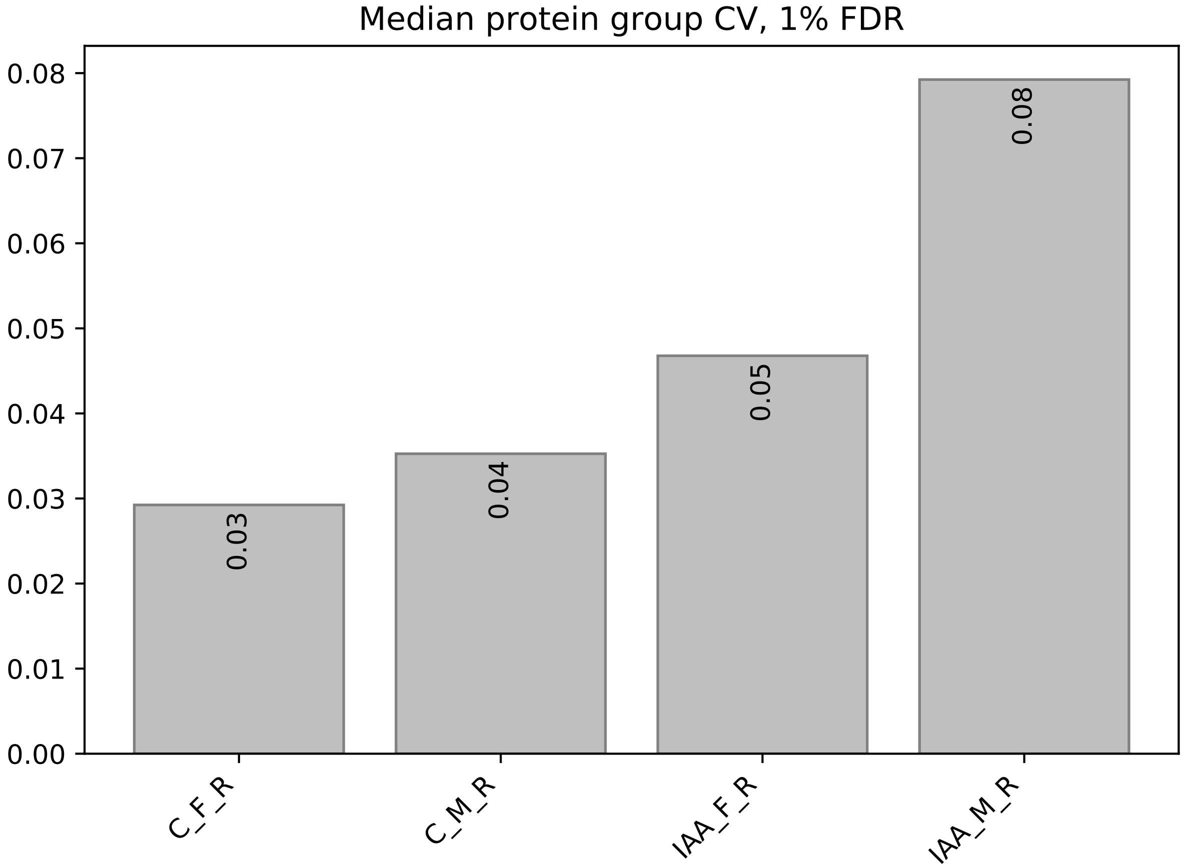

Median protein group CV, 1% FDR
0.00
0.01
0.02
0.03
0.04
0.05
0.06
0.07
0.08
0.03
0.04
0.05
0.08
C_F_R
C_M_R
IAA_F_R
IAA_M_R

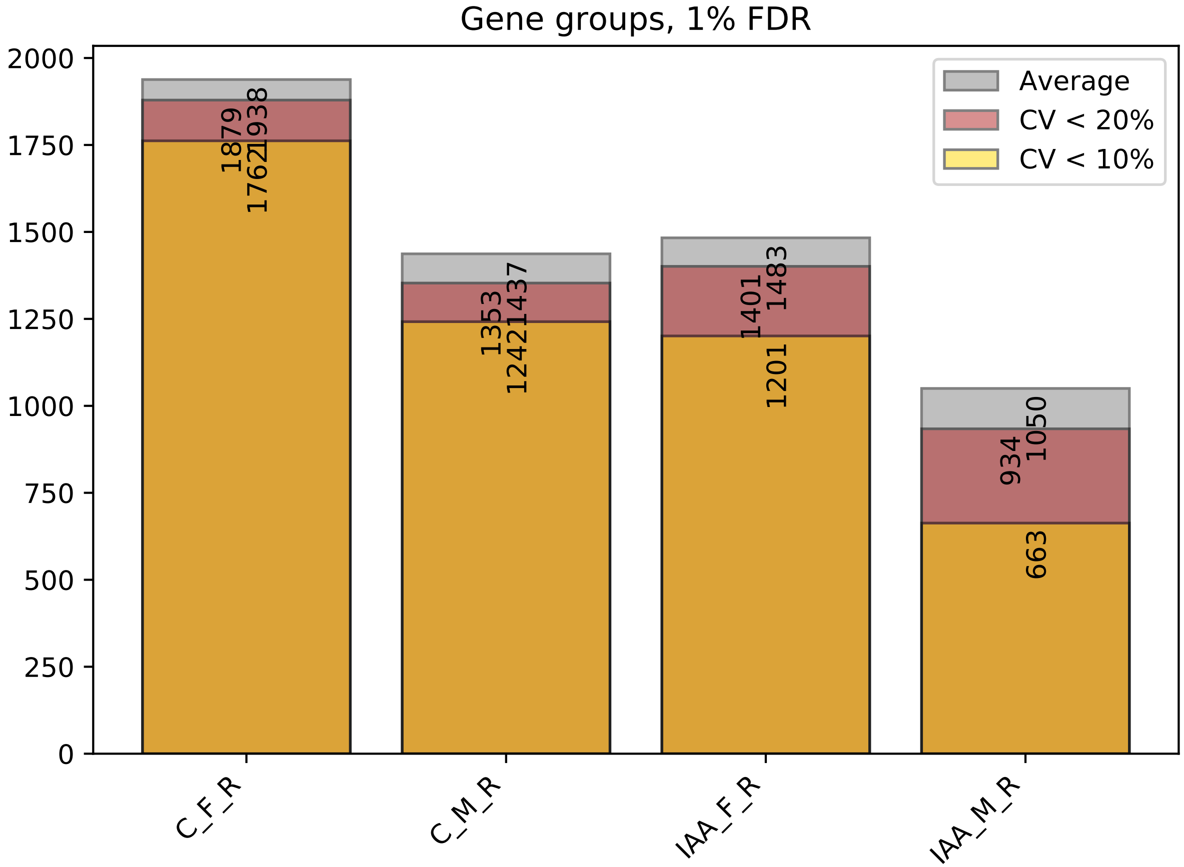

Gene groups, 1% FDR
Average
CV < 20%
CV < 10%
2000
1750
1500
1250
1000
750
500
250
0
1879
1938
1762
1353
1437
1242
1401
1483
1201
934
1050
663
C_F_R
C_M_R
IAA_F_R
IAA_M_R

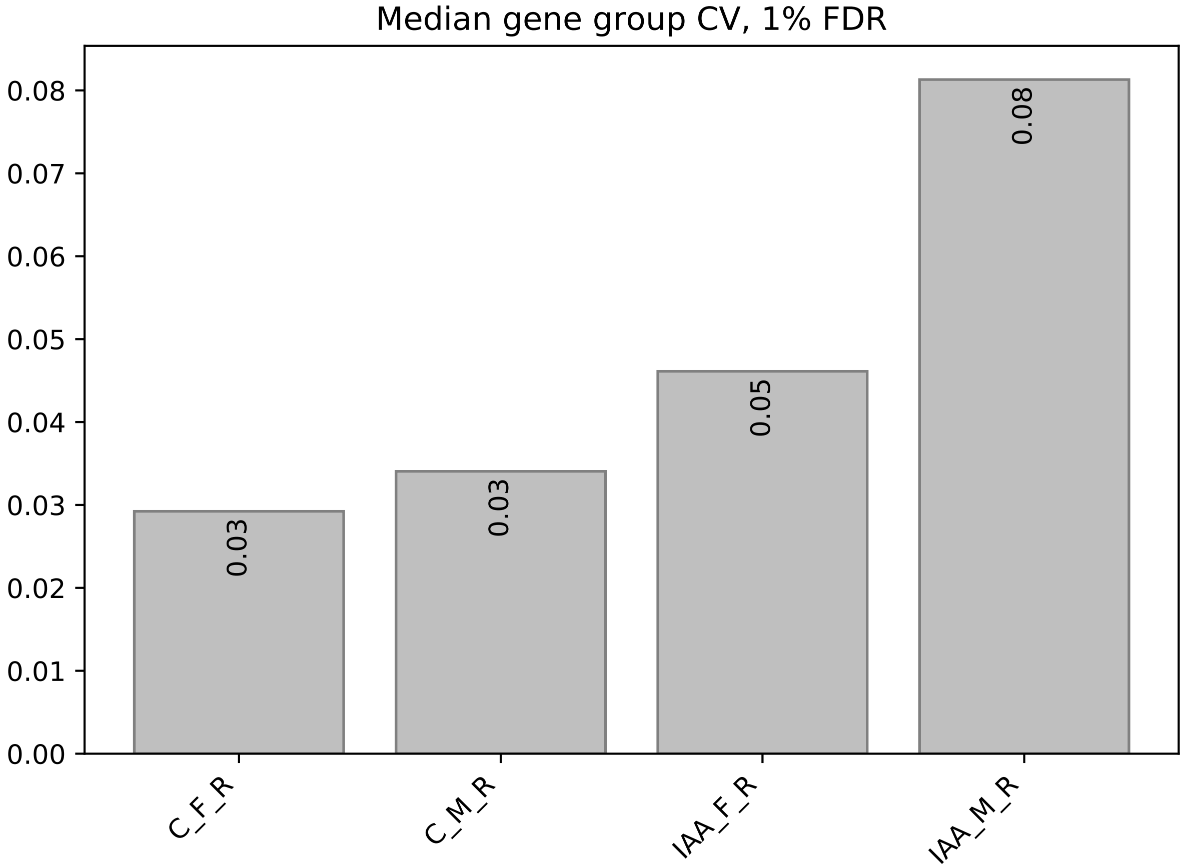
Median gene group CV, 1% FDR
0.00
0.01
0.02
0.03
0.04
0.05
0.06
0.07
0.08
0.03
0.03
0.05
0.08
C_F_R
C_M_R
IAA_F_R
IAA_M_R
